# Supplementary material for: A randomized, double-blind, placebo-controlled trial of niclosamide nanohybrid for the treatment of patients with mild to moderate COVID-19
Source: Nat Commun. 2025 Aug 1;16:7084. doi: 10.1038/s41467-025-62423-4 (PMC12317003; doi:10.1038/s41467-025-62423-4)
Supplement: Supplementary file 1 — Supplementary Information [file 41467_2025_62423_MOESM1_ESM.pdf]

Supplementary Information
A randomized, double-blind, placebo-controlled trial of niclosamide nanohybrid for the treatment of patients with mild to moderate COVID-19

Table of contents

Contents		Pages
**Study protocol**		**2–17**
	Definitions of Abbreviations and Terms	**3–4**
	Protocol summary	**5–14**
	Key milestones	**15–17**
**Adverse reactions**		**18–26**
	Table 1. Adverse events	**18**
	Table 2. Adverse events emerged after treatment	**19**
	List 1. List of adverse events emerged after treatment	**20–21**
	List 2. List of adverse events emerged after treatment by grade	**22–23**
	List 3. List of adverse events emerged after treatment – considered to be related to drug or the placebo	**24–26**
**Supplementary Methods**		**25-32**
**Supplementary Note 1**		**32-35**
**Supplementary Figures/Tables**		**36-52**
**Supplementary Note-2 Secondary Endpoints**		**53-137**
**Supplementary References**		**137-138**

임상시험계획서
[Clinical Study Protocol]

경증 또는 중등증 코로나바이러스감염증-19 환자를 대상으로 CP-COV03의 유효성 및 안전성을 비교 평가하기 위한 무작위배정, 이중눈가림, 위약대조 제 2 상 임상시험

A randomized, double-blind, placebo-controlled trial of niclosamide nanohybrid for the treatment of patients with mild to moderate COVID-19

**Protocol No.:**	**DTC22-IP006**
**Version:**	**8.0 (29 Dec 2022)**
**Phase of Study:**	**Phase II**
**Sponsor:**	**Hyundai Bioscience Co., Ltd.**

CONFIDENTIAL

All information related to this study protocol should be treated as confidential information of Hyundai Bioscience Co., Ltd., provided for the principal investigator and investigators, the Institutional Review Board, and the Ministry of Food and Drug Safety. It cannot be disclosed and rewritten, or copied/distributed to third parties without the approval of Hyundai Bioscience Co., Ltd., except for obtaining written consent from the subjects participating in this study to administer the investigational product.

Definitions of Abbreviations and Terms

Investigational Product	Investigational product, 임상시험에 사용되는 의약품
Treatment group	Treatment group, 투여한 임상시험용 의약품의 종류, 용량 등 투약방법에 따라 분류한 군
ADR	Adverse Drug Reaction, 약물이상반응
AE	Adverse Event, 이상반응
ALP	Alkaline Phosphatase, 알칼리성 인산가수분해효소
ALT	Alanine Transaminase, 알라닌 아미노기전달효소
ANOVA	Analysis of Variance, 분산분석, 두 개 이상의 집단의 평균이 동일한지 여부를 모수적으로 검정하는 분석방법
AST	Aspartate Transaminase, 아스파르트산 아미노기전달효소
AUC_{last}	Area under the plasma drug concentration-time curve over the time interval from 0 to the last quantifiable plasma concentration, 측정이 가능한 마지막 채혈시점까지 계산한 혈중 농도-시간 곡선하 면적
BUN	Blood Urea Nitrogen, 혈중 요소 질소
CK	Creatine Kinase, 크레아틴 키나제
CL/F	Apparent Clearance, 겉보기 청소율
C_{max}	Maximum concentration of drug 최고혈중농도
(e-)CRF	(electronic) Case Report Form, (전자)증례기록서
CRP	C-Reactive Protein, C 반응성 단백질
DSMB	Data Safety Monitoring Board, 데이터 안전성 모니터링 위원회
e-GFR	estimated Glomerular Filtration Rate, 사구체 여과율
GCP	Good Clinical Practice, 의약품 임상시험 관리기준
γ-GTP	gamma-Glutamyl Transpeptidase, 감마글루타밀 전이효소
h, hr	Hour, 시간
HBsAg	Hepatitis B Virus surface Antigen, B 형간염표면항원
hCG	Human chorionic Gonadotropin, 사람 융모성 성선자극호르몬
HCV Ab	anti-Hepatitis C Virus Antibody, C 형간염항체
HIV Ab	anti-Human Immunodeficiency Virus Antibody, 사람면역결핍바이러스 항체
ICH	International Council on Harmonization of Technical Requirements for Registration of Pharmaceuticals for Human Use, 국제의약품규제조화위원회
IRB	Institutional Review Board, 임상연구심사위원회
LDH	Lactate Dehydrogenase, 젖산탈수소효소
MRSD	Maximum Recommended Starting Dose, 임상시험에서 사람에게 투여를 허용할 수 있는 최대 허용량
NIH	National Institutes of Health, 미국 국립 보건원
NOAEL	No Observed Adverse Effect Level, 부작용이 관찰되지 않는 최대량
PSV	Post Study Visit, 종료 방문

RBC	Red Blood Cell, 적혈구
SOP	Standard Operating Procedure, 표준작업지침서
SAS®	Statistical Analysis System, 통계 분석을 위한 소프트웨어
$t_{1/2}$	Terminal half-life, 소실반감기
T_{max}	Time of maximum concentration, 약물 투여 후 최고혈중농도 도달시간
Vd/F	Volume of Distribution, 약물분포용적
WBC	White Blood Cell, 백혈구

PROTOCOL SUMMARY

<table>
<tr><td>Title</td><td>A randomized, double-blind, placebo-controlled design phase 2 clinical trial to comparatively evaluate the efficacy and safety of CP-COV03 in patients with mild or moderate COVID-19</td></tr>
<tr><td>Objective</td><td>To exploratorily evaluate the safety and efficacy of the investigational product in patients with mild and moderate COVID-19</td></tr>
<tr><td>Study Institution and Principal Investigator</td><td>Refer to [Appendix 5. Study Institution and Principal Investigator]</td></tr>
<tr><td>Sponsor</td><td>Hyundai Bioscience Co., Ltd.
106, Apogongdan-gil, Apo-eup, Gimcheon-si, Gyeongsangbuk-do, Republic of Korea</td></tr>
<tr><td>Investigational Product</td><td>1) Study drug: CP-COV03-T (Niclosamide 50 mg, Hyundai Bioscience Co., Ltd.)
2) Reference drug: CP-COV03-R (Niclosamide 50 mg the placebo, Hyundai Bioscience Co., Ltd.)</td></tr>
<tr><td>Study Phase</td><td>Phase 2 clinical trial (Phase II)</td></tr>
<tr><td>Indication</td><td>Coronavirus Disease-19 (COVID-19)</td></tr>
<tr><td>Study Design</td><td>Randomized, Double blinded, the placebo-controlled Trial
<table>
<tr><th>Group</th><th>Dose (mg/day)</th><th>Subject (persons)</th><th colspan="2">Administration Method
(3 times a day for 5 days)</th></tr>
<tr><td rowspan="3">Study group 1</td><td rowspan="3">900
(the low dose)</td><td rowspan="3">100</td><td>#1</td><td>CP-COV03-T 6 capsules + CP-COV03-R 3 capsules</td></tr>
<tr><td>#2</td><td>CP-COV03-T 6 capsules + CP-COV03-R 3 capsules</td></tr>
<tr><td>#3</td><td>CP-COV03-T 6 capsules + CP-COV03-R 3 capsules</td></tr>
<tr><td rowspan="3">Study group 2</td><td rowspan="3">1,350
(the high dose)</td><td rowspan="3">100</td><td>#1</td><td>CP-COV03-T 9 capsules</td></tr>
<tr><td>#2</td><td>CP-COV03-T 9 capsules</td></tr>
<tr><td>#3</td><td>CP-COV03-T 9 capsules</td></tr>
<tr><td rowspan="3">Control group</td><td rowspan="3">the placebo</td><td rowspan="3">100</td><td>#1</td><td>CP-COV03-R 9 capsules</td></tr>
<tr><td>#2</td><td>CP-COV03-R 9 capsules</td></tr>
<tr><td>#3</td><td>CP-COV03-R 9 capsules</td></tr>
</table></td></tr>
<tr><td>Number of subjects</td><td>300 subjects

Rationale for Sample Size Calculation
This study is exploratory and descriptive in nature as it is to evaluate the safety and efficacy of CP-COV03 by the dosage/dose. Therefore, it is different from the typical study for testing statistical hypothesis, and conducted in a total of 300 subjects (100 in each group), the number of subjects which is expected to suitable for comparison/assessment of the safety and efficacy of the study drug and the reference drug.</td></tr>
</table>

Inclusion/ Exclusion Criteria

Inclusion Criteria

1) Subjects who voluntarily determine to participate in this study after listening the detailed information and consent in writing (electronically) to comply with the study requirements.
2) Adult aged 19 and over at the time of screening
3) Subjects who are confirmed to have COVID-19 through RT-PCR test or rapid antigen kit test by a specialist within 3 days of randomization
4) Subjects who have developed one or more of the symptoms of COVID-19* within 5 days of randomization and have at least two symptoms with a score of 2 or higher among the symptom scores** determined on the day of randomization
   - * Symptoms of COVID-19: Fever (38.0°C or above), cough, sore throat, headache, myalgia, chills/tremor, nasal discharge/nasal obstruction, fatigue/malaise, dyspnea/shortness of breath, sickness/nausea, vomiting, diarrhea
   - ** Symptoms are assessed based on a total of 4 levels: 0 = Absent, 1 = Mild, 2 = Moderate, and 3 = Severe.
5) Subjects who are diagnosed as mild or moderate according to the NIH severity criteria at screening and randomization.

<NIH COVID-19 Severity Criteria>

Severity	Comments
Asymptomatic	Individuals who test positive for COVID-19 but have no symptoms that are consistent with it.
Mild	Individuals who have any of the various signs and symptoms of COVID-19 but do not have shortness of breath, dyspnea, or abnormal chest imaging.
Moderate	Individuals who show evidence of lower respiratory disease during clinical assessment or imaging and who have an oxygen saturation (SpO_2) ≥94% on room air at sea level.
Severe	Individuals who have SpO_2 <94% on room air at sea level, PaO_2/FiO_2 <300 mmHg, a respiratory rate >30 breaths/min, or lung infiltrates >50%.
Critical	Individuals who have respiratory failure, septic shock, and/or multiple organ dysfunction.

Exclusion Criteria

1) Subjects who have one or more of the following severe findings at screening:
   - Oxygen saturation(SpO_2) less than 94% on room air at sea level
   - Respiratory rate greater than 30 breaths per minute
   - PaO_2/FiO_2 <300 mmHg (Test can be omitted if it is medically unnecessary, when considering oxygen saturation, respiratory rate, etc.)
   - Lung infiltrates >50% on chest imaging
2) Subjects who require oxygen therapy (e.g., supplemental oxygen, non-invasive ventilation, invasive ventilation, extracorporeal membrane oxygenation, etc.) or have serious conditions such as respiratory failure, shock, multiple organ failure, etc.

	3) Subjects who have hypersensitivity to the main active ingredient (Niclosamide) and components of the investigational product<br>4) Subjects who have genetic problems such as lactose intolerance, galactose intolerance, Lapp lactase deficiency, and glucose-galactose malabsorption<br>5) Subjects with renal impairment (eGFR < 30 mL/min/1.73 m^2)<br>6) Subjects with hepatic impairment (ALT or AST >5 times the upper limit of normal organ)<br>7) Cirrhotic patients with a Child-Pugh score of B or C

	A	B	C
Ascites	None	Easily controlled	Poorly controlled
Bilirubin	< 2 mg/dL	2~3 mg/dL	> 3 mg/dL
Albumin	> 3.5 g/dL	3.0~3.5 g/dL	< 3.0 g/dL
Encephalopathy	None	Mild	Advanced
Nutritional status	Excellent	Good	Poor

	8) Subjects who have gastrointestinal disorders or undergone surgeries that may affect drug absorption, distribution, metabolism, and excretion (except for simple appendicectomy or hernia surgery)<br>9) Subjects who have received drugs such as antiviral drugs, neutralizing antibody therapy, plasma fractionation therapy, and immune modulators (cytokine therapy, etc.) for the treatment of COVID-19 infection within 28 days of screening. However, after a sufficient drug-free interval, the patient may be eligible for participating in the study.<br>10) Subjects who have or had one or more of the following infectious diseases:<br>- Viral diseases other than COVID-19 that require administration of other antiviral drugs (e.g., HIV, HBV, HCV, etc.)<br>- Serious infections that require systemic anti-infective treatment other than COVID-19 infection within 30 days prior to administration of the investigational product<br>11) Pregnant or lactating women<br>12) Fertile women and men who plan to become pregnant or do not intend to use the following appropriate contraceptive methods* during the clinical study and for 3 months after the study termination<br>* Hormonal contraception, intrauterine device, double barrier method (both male (condom) and female (contraceptive diaphragm, contraceptive sponge or cervical cap) should use), sterilization (vasectomy, tubal ligation, etc.)<br>13) Subjects who have participated in other clinical trials of a drug/medical device within 28 days of the consent form sign date and have been administered/treated with that investigational product/medical device<br>14) Subjects who have chronic underlying diseases that are unsuitable for participation in the study in the opinion of the investigator, and who are determined by the investigator to be inappropriate to participate in this clinical study for other reasons
**Study Methods**	This clinical trial is conducted as a randomized, double-blind, the placebo-controlled study. In patients with COVID-19 who voluntarily agreed to participate in this study, screening tests such as history taking, physical examination, chest imaging, electrocardiogram, and clinical laboratory tests are performed by Day -1 to select subjects determined to be eligible. Among the selected subjects, the first 18 enrolled subjects are assigned in the ratio of 2:1 to the study group 1 (low dose) and control group in order to confirm the safety. If the safety is confirmed in the low-dose sentinel group, 18 subjects who are enrolled subsequently will be assigned in the ratio of 2:1 to the study group 2 (high dose) and control group. If the safety is also confirmed in the high-dose sentinel group, additional subjects are subsequently enrolled.

	The enrolled subjects will be administered with the investigational product from Day 1 to Day 6, and the safety and efficacy assessments are conducted according to the study schedule. Investigators will interview all subjects in person or on the phone every day during hospitalization. Subjects who are discharged during the study will visit the study site on Day 8, Day 14, and Day 28 for the efficacy and safety assessments, etc.<br>Other drugs used before participation in this study are acceptable only as specified in this study protocol, and the subjects receiving antiviral drugs or immunosuppressants due to infections other than COVID-19 are excluded according to the exclusion criteria.<br><br>**Screening (Day -2 ~ Day -1)**<br>After providing an explanation of the clinical study to patients within 5 days after onset of COVID-19 symptoms and within 3 days after confirmation and obtaining voluntary consent in writing (electronically) from the patients, screening tests, including collection of demographic information, chest imaging tests, electrocardiogram, and clinical laboratory tests, and procedures such as severity determination are conducted.<br><br>**Baseline (Day 0)**<br>Subjects who met the inclusion/exclusion criteria are randomized in a ratio of 1:1:1 to the study group 1, study group 2, or the placebo group. However, the subjects are stratified according to age, severity of COVID-19, and whether they agree to pharmacokinetic blood sampling. Randomization will be conducted with 18 subjects in the low-dose sentinel group, 18 in the high-dose sentinel group, and a follow-up group for the safety assessment.<br><br>**Treatment (Day 1 ~ Day 6)**<br>To confirm the safety according to the dosage/dose, the first 18 subjects are assigned to the study group 1 and control group in a ratio of 2:1 and then administered with the investigational drug. From Day 1 to Day 8, if no adverse events corresponding to the suspension criteria are identified by observing adverse events in all subjects, the next 18 subjects will be assigned to the study group 2 and control group in a 2:1 ratio. If an adverse event corresponding to the suspension criteria occurs within 8 days from the first dose of the investigational product in the low-dose sentinel group, the DSMB will be held to evaluate the safety information collected up to 8 days after administration of the investigational product and determine whether to proceed with enrollment of the high-dose sentinel group. If an adverse event corresponding to the suspension criteria occurs in the subjects of high-dose group up to 8 days from the first dose of the investigational product, the DSMB will be held to evaluate safety and determine whether to continue the study. The DSMB will review the safety data of the first 36 subjects to confirm whether the study may continue or not, even if no adverse events corresponding to the suspension criteria occur in the low-dose and high-dose groups.<br><Adverse events corresponding to the suspension criteria><br>①. If, by the investigator's judgment, the same or similar gastrointestinal adverse events of Grade 3 or higher related to the investigational product are observed in 4 or more subjects in the study group (Grade classification is based on the following criteria).<br><Grade Classification>

Grade / Item	Grade 1	Grade 2	Grade 3	Grade 4
Vomiting	Does not interfere with daily activities, and occurs 1-2 times in 24 hours	Slightly interferes with daily activities, and occurs more than 2 times in 24 hours	Seriously interferes with daily activities or requires fluid therapy	Emergency visit or hospitalizations due to hypotensive shock

Diarrhea	2-3 times of loose stools or less than 400 g in 24 hours	4-5 times of loose stools or 400~800 g in 24 hours	6 or more times of loose stools in 24 hours, 800 g or more, or requiring fluid therapy	Emergency visit or hospitalization
Abdominal pain	Mild	Moderate (does not require treatment)	Moderate (requires treatment)	Severe (hospitalization)
Other	Temporary or brief discomfort (within 48 hours); does not require medical intervention/treatment and not affect daily activities	Mild to moderate restrictions on daily activities (capable of performing more than 50% of usual activities), some assistance may be needed (does not or minimally require medical intervention/treatment)	Significant restrictions on daily activities (less than 50% of usual activities), generally some assistance is required (requires medical intervention/treatment, possibly hospitalization)	Requires significant assistance due to life threatening and severe restrictions on daily activities (requires medical intervention/treatment, highly likely to be hospitalized), serious or life-threatening adverse events

②. When serious adverse drug reactions related to the investigational product are observed in 2 or more subjects by the investigator's judgment

③. Other clinically significant medical conditions that make it difficult for the subject to participate in the study at the judgment of the investigator.

If the DSMB determines to continue the study, then 264 subjects will be assigned to the study group 1, study group 2, and control group in a 1:1:1 ratio. However, only 60 applicants are assigned to the study group 1, study group 2, and control group in a 1:1:1 ratio to whom additional pharmacokinetic blood sampling is conducted (60 applicants who agreed to PK blood sampling will be assigned without stratification, and the remaining 204 applicants who did not agree to PK blood sampling will be assigned stratified according to age and severity).

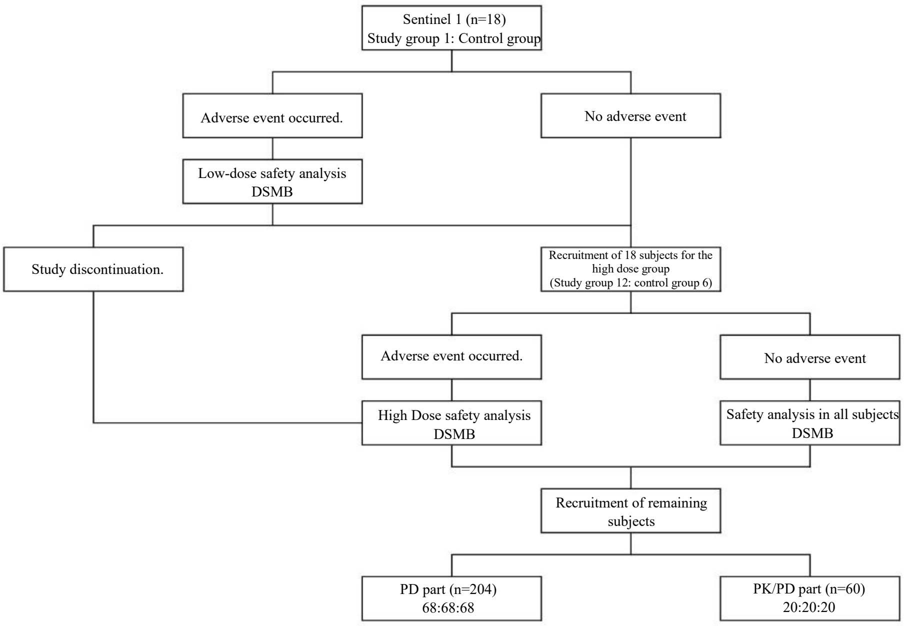

The investigational product should be administered within 1 day from the date of randomization. Randomized subjects will take orally 9 capsules 3 times a day for 5 days from the evening of Day 1 to the lunch of Day 6 and proceed with the study according to the study schedule. The investigational product is administered usually at about 8-hour intervals before breakfast, about 2-4 hours after lunch, and about 2-4 hours after dinner, on an empty stomach if possible.

After the first two doses, the subject starts the symptom assessment from the morning of Day 2 and evaluates the symptoms every morning and evening. The assessment is carried out at similar times as possible during the study. Symptom scores are determined based on the severity of the worst symptoms in the past 12 hours by referring to the symptom scoring criteria.
In case of worsening of symptoms requiring oxygen treatment, the administration of the investigational product shall be discontinued by the investigator's judgment, and the study procedure other than administration of the investigational product may be performed. If the investigator determines that it is impossible or risky for the subject to continue the study procedure, the subject will be withdrawn and provided with standard of care.

Follow up (Day 7 ~ Day 28)
Subjects who have completed the planned study follow-up period schedule for Day 6 can be discharged from Day 6 if they meet the conditions of the COVID-19 Response Guidelines.

Outpatients visits will be made on Days 8, 14, and 28, and safety and efficacy are investigated through prepared patient diary on the other days. In the patient diary, adverse events, concomitant medications (or rescue medications), and status of symptoms are recorded. In particular, the subject must record in detail the occurrence of gastrointestinal adverse events (vomiting, diarrhea, heartburn, upper abdominal pain, abdominal distention, dehydration, hematochezia, etc.) in the diary and notify the investigator of vomiting, diarrhea, and abdominal pain of Grade 3 or higher immediately, if any. The investigator should take appropriate medical measures such as outpatient treatment or hospitalization for the subject.

For subjects who have withdrawn consent or dropped out of the study, safety investigation corresponding to Day 28 shall be conducted within 7 days from the time when the withdrawal

	is determined.
**Endpoints**	**Primary Efficacy Endpoints**

Time (days) taken for the symptoms of COVID-19* to improve** and continue for more than 48 hours by Day 14***.

* The scoring criteria for COVID-19 symptoms are as follows (the scoring criteria are as below, and the detailed criteria provided separately to study sites and subjects are found in Appendix 7).

Score / Symptom	0 (none)	1 (mild)	2 (moderate)	3 (severe)
Fever	Less than 38.0℃	38.0~38.4℃	38.5~38.9℃	39.0℃ or higher
Cough	None	Mild	Moderate	Severe
Sore throat	None	Mild	Moderate	Severe
Headache	None	Mild	Moderate	Severe
Myalgia	None	Mild	Moderate	Severe
Chills/tremor	None	Mild	Moderate	Severe
Nasal discharge/ obstruction	None	Mild	Moderate	Severe
Fatigue/malaise	None	Mild	Moderate	Severe
Dyspnea/ shortness of breath	None	Mild	Moderate	Severe
Sickness/Nausea	None	Mild	Moderate	Severe
Vomiting	None	1–2 times in 24 hours	3–4 times in 24 hours	5 times or more in 24 hours
Diarrhea	None	1–2 times in 24 hours	3–4 times in 24 hours	5 times or more in 24 hours

** If the symptoms of COVID-19 have been determined to be improved

- If the symptom observed at baseline as a score of 2 or more has been improved to less than or equal to 1
- If the symptom observed at baseline as a score of 1 has been improved to 0
- If the symptom, which was not observed at baseline but newly developed during the study, has been improved to 0 again

*** Duration: Day of observation of symptom improvement over 48 hours – day of the first dose + 0.5 days

Secondary Efficacy Endpoints

1) Time (days) taken for each COVID-19 symptom to improve and continue for more than 48 hours by Day 14
2) Time (days) taken for each COVID-19 symptom to improve by Day 14
3) Time (days) taken for the scores of each COVID-19 symptom to decrease by 1 point or more by Day 14
4) Changes in the total score and mean of all COVID-19 symptoms by Day 14

	(asymptomatic items are excluded from the mean calculation)<br>5) Time (days) taken for the total score of COVID-19 symptoms to decrease by 25% or more and 50% or more by Day 14<br>6) Presence and number of the appearance of new symptoms derived from COVID-19 other than pre-defined symptoms by Day 14<br>7) Changes in SARS-CoV-2 viral load on Day 2, Day 4, Day 6 and Day 8 compared to before administration of the investigational product<br>8) Among the rescue medications used on Day 1 to Day 28, the dose and dosing frequency of Acetaminophen, Ibuprofen, and antidiarrheal drugs<br>9) Proportion of subjects who worsened to severe from Day 1 to Day 28<br>10) Pharmacokinetic characteristics of niclosamide and correlation between pharmacokinetic parameters and viral load<br><br>**Safety Assessment Items**<br>1) Adverse event<br>2) Vital signs (temperature, blood pressure, heart rate, breathing rate, oxygen saturation)<br>3) Laboratory tests<br>4) Thoracic(Chest) imaging test<br>5) Electrocardiogram
**Statistical Analysis**	**General Statistics**<br>The safety assessment is conducted on all subjects who have received at least one dose of the investigational product. The main analysis set of the efficacy assessment is defined as ITT (Intend to Treat), while PPS (Per Protocol Set) is also analyzed.<br>- ITT: All subjects who were randomized and received the investigational product<br>- PPS: Subjects who completed the planned study follow-up period without serious protocol violations<br>Statistical analysis is performed using SAS®, and other programs can be used as needed.<br><br>**Analysis of Demographic Data**<br>For the demographic information and other pre-treatment characteristics of all randomized subjects, descriptive statistical analysis such as mean and standard deviation is performed for continuous variables, while frequency etc. are summarized and analyzed for categorical variables.<br><br>**Efficacy Analysis**<br>In the primary efficacy endpoints, the comparison between administration groups is performed using the Cox Proportional Hazards Regression Model with the administration group as a factor and the stratification factor (age, severity) as a covariate, and a 95% confidence interval and p-value corresponding to the hazard ratio of the administration group are presented.<br>In the secondary efficacy endpoints, for the comparison between before and after administration of the investigational product, the frequency and percentage for each administration group are presented in the case of a proportion (categorical variable). The difference between the administration groups is analyzed using the Logistic Regression Model with the administration group as a factor and the stratification factor (age, severity) as a covariate.<br>For the amount of change (continuous variable), descriptive statistics of the change at each time point for each administration group are presented. Comparison between administration groups is analyzed using the ANCOVA model with the administration group as a factor and the baseline value and stratification factor (age, severity) as covariates. Other variables, including period, doses, self-assessment scores, etc., are analyzed in the same way.

	**Safety Analysis**<br>All adverse events that occur in this study are standardized to System Organ Class (SOC) and Preferred Term (PT) based on the MedDRA dictionary (24.1 or later). In addition, for adverse events and drug adverse reactions that occur in this study, the frequency (number of subjects), percentage, and number of occurrences are presented based on SOC and PT by the administration group, and for adverse events and drug adverse reactions occurred by severity, the frequency (number of subjects), percentage, and number of occurrences are presented based on SOC and PT as well. They are presented in a table, but summarized by administration group, severity, and causal relationship with the investigational product. The Chi-square test or Fisher's exact test is used to analyze whether there is a difference in the incidence of adverse events, adverse drug reactions, and serious adverse events between administration groups.<br><br>For vital signs, electrocardiograms, clinical laboratory tests, and physical examinations, continuous data are analyzed using paired t-test or Wilcoxon's signed rank test depending on whether the normality assumption is satisfied, while categorical variables are analyzed using McNemar's test in order to determine changes within the administration group. To confirm the changes between administration groups, continuous variables are analyzed using ANOVA or Kruskal–Wallis test depending on whether the normality assumption is satisfied, and categorical variables are analyzed using Chi-square test or Fisher's exact test.<br><br>Concomitant medications are standardized using the WHO ATC classification system, and subjects who have received concomitant medications during the study are listed. In other observations and test items of the subjects such as vital signs diagnostic tests, etc., continuous data are analyzed descriptively, whereas categorical data are presented as frequency and percentage.

KEY MILESTONES

Schedule	Screening	Baseline*	Treatment						Follow up					
	Day -2 ~ Day -1	Day 0	Day 1	Day 2	Day 3	Day 4	Day 5	Day 6	Day 7	Day 8	Day 9~13	Day 14±2	Day 15~27	Day 28±4
Written (electronic) ICF acquisition	●													
Confirmation of demographic information	●													
Check the date of symptom onset/confirmation	●													
Check severity	●	●	●	●	●	●	●	●		●		●		●
Medical/surgical history check[1)]	●													
Preceding/concomitant medication check[2)]	●	●	●	●	●	●	●	●	●	●	●	●	●	●
Physical examination	●													
Vital signs[3)]	●	●	●	●	●	●	●	●		●		●		●
Symptom assessment[4)]	●	●		●	●	●	●	●	●	●	●	●	●	●
Electrocardiogram	●							●						◐
Pregnancy test[5)]	●													●
Clinical laboratory test[6)]	●	●	◐	◐	◐	◐	◐	●				◐		◐
Thoracic(Chest) imaging test[7)]	●	●	◐	◐	◐	◐	◐	●				◐		◐
Inclusion/exclusion criteria assessment[8)]	●	●												
Randomization		●												
Hospitalization		●												
Discharge[9)]								●						
Outpatient visit										●		●		●

Patient diary** dispense			◐					●						
Patient diary recover														●
Viral load measurement[10)]		●		●		●		●		●				
Investigational product administration[11)]			●	●	●	●	●	●						
Medication compliance check			●	●	●	●	●	●						
Rescue medication check[12)]			●	●	●	●	●	●	●	●	●	●	●	●
Pharmacokinetic blood sampling[13)]			●	●	●	●	●	●						
Adverse event check[14)]			●	●	●	●	●	●	●	●	●	●	●	●

* Screening, Baseline, and Day 1 can be performed on the same day, and the administration from Screening to Day 1 shall be completed within 48 hours.

** Application can be used alternatively, and if the application is used, symptom assessment, adverse events, and concomitant medications during hospitalization can also be collected through the application.

1) Medical/surgical history check	Medical history (including surgical history) within 4 weeks prior to screening is collected.
2) Preceding/concomitant medications	For preceding medications, those administered from 4 weeks before screening until administration of the investigational product are collected.<br>For concomitant medications, those administered after administration of the investigational product are collected.<br>Acetaminophen, Ibuprofen, and antidiarrheals are collected as efficacy assessment items.<br>After patient's discharge, these are recorded in the patient diary.
3) Vital signs	Blood pressure, heart rate, respiration rate, body temperature, and oxygen saturation are measured. Except for body temperature and oxygen saturation, they are measured once a day.<br>When a subject is hospitalized/admitted, the body temperature and oxygen saturation are measured three times a day, where the highest result of the body temperature and the lowest of oxygen saturation measurement are used. If necessary, the body temperature and oxygen saturation can be measured more than 3 times a day.<br>If screening and baseline are performed on the same day, vital signs on Day 1 are performed before the first dose. If the baseline and Day 1 are performed on the same day, the vital signs of Day 1 can be replaced with those of the baseline. If Screening to Day 1 administration are performed on the same day, the vital signs measured at screening are substituted for those at baseline and on Day 1.<br>Vital signs from Day 2 to Day 6 are measured after the noon dosing.
4) Symptom assessment	Symptom assessment is conducted throughout the study.<br>The symptom assessment after administration starts from the morning of Day 2 and then performed every morning and evening (2 times a day). The assessment is carried out at similar times if possible during the study.<br>The morning symptom assessment is for the symptoms of previous night, while the evening symptom assessment is for the symptoms during the day.<br>After patient's discharge, these are recorded twice a day in the patient diary.
5) Pregnancy test	Only women of childbearing potential are subject to the Urine hCG test at screening and on Day 28.
6) Clinical laboratory tests	The following tests are performed at screening, baseline, and on Day 6. During the study period, only some of the tests can be performed when necessary according to the judgment of the investigator. The clinical laboratory test results at baseline can be replaced with the those performed at screening. However,

		immunoglobulin tests are performed only at screening.<br>• Hematology: WBC, RBC, Hemoglobin, Hematocrit, Platelet, WBC with differential count (Neutrophils, Lymphocytes, Monocytes, Eosinophils, Basophils)<br>• Blood chemistry: Glucose, BUN, Uric acid, Total protein, Albumin, Total bilirubin, Alkaline phosphatase, AST, ALT, γ-GT, LDH, Creatinine, CPK, Na, K, Cl, Calcium, Phosphorus, eGFR, CRP, Lipase, Amylase<br>• Blood coagulation test: PT, PT-INR (calculation formula), PT(%), APTT<br>• Urinalysis: Specific gravity, pH, Protein, Glucose, Ketone, Color, Leukocyte, Bilirubin, Occult blood, Urobilinogen, Nitrite<br>• Immunoglobulin test: HBsAg, anti-HCV Ab, HIV Ag/Ab, Syphilis Regain test
7)	Thoracic(Chest) imaging test	Thoracic imaging tests are performed at screening, baseline, and on Day 6 and can also be performed when necessary according to the judgment of the investigator.
8)	Assessment of inclusion/exclusion criteria	In the baseline, it is confirmed whether there are no changes in the evaluation conducted at the screening.<br>If there is a change in the COVID-19 symptom score, the baseline score will be used. Therefore, if it is confirmed that the baseline symptom score is lower than that confirmed at the screening, the subject may be dropped out according to the inclusion criteria.
9)	Discharge	Subjects who have completed the administration may be discharged if they meet the conditions of the COVID-19 response guidelines.<br>Hospitalization may be continued depending on the condition of the subject.
10)	Viral load measurement	The sample is collected after the lunch administration on Days 2, 4, and 6. On Day 8, the sample is collected at the outpatient visit.
11)	Administration of the drug	The administration starts at the evening of Day 1 and ends at lunch of Day 6. A total of 9 capsules are administered orally 3 times a day at intervals of about 8 hours.
12)	Check rescue medications	The dose and dosing frequency of administering Acetaminophen, Ibuprofen, and antidiarrheal are checked.<br>When discharged, it is recorded in the patient diary.
13)	Pharmacokinetic blood sampling	Day 1 - Before and 3 hours after the evening dosing (2 times/day)<br>Day 2 - Before and 3 hours after the morning and noon dosing (4 times/day)<br>Day 3 to Day 4 - Before and 3 hours after the noon dosing (2 times/day)<br>Day 5 - Before and 3 hours after the noon and evening dosing (4 times/day)<br>Day 6 - Before and 3 hours after the morning and noon dosing (4 times/day)
14)	Adverse event	When discharged, it is recorded in the patient diary.

Table 1. Adverse events

Adverse event category	The placebo N=98 n (%)[case]	The low dose group N=99 n (%)[case]	The high dose group N=96 n (%)[case]
Patients without adverse events	50(51.0)	53(53.5)	49(51.0)
Adverse events emerged prior to treatment	30(30.6)[38]	30(30.3)[35]	23(24.0)[31]
Adverse Events emerged after treatment	26(26.5)[37]	21(21.2)[32]	33(34.4)[47]

Table 2. Adverse events emerged after treatment

Category of adverse events emerged after treatment	The placebo N=37 case(%)	The low dose group N=32 case(%)	The high dose group N=47 case(%)	p-value
Grade of adverse event				0.6559†
Mild	32(86.5)	25(72.1)	39(83.0)	
Moderate	5(13.5)	7(21.9)	8(17.0)	
Severe	-	-`	-	
Serious adverse event	0	0	0	-
Events considered to be related to drug or the placebo	19(51.4)	12(37.5)	22(46.8)	0.5049†
Drug/IP dose because of events				0.6633†
Drug Interrupted/Reduced	-	-	-	
Dose Maintained	17(45.9)	13(40.6)	17(36.2)	
Not Applicable	20(54.1)	19(59.4)	30(63.8)	

† Chi-square test

List 1. List of adverse events emerged after treatment

Adverse events emerged after treatment	The placebo N=37 case(%)	The low dose group N=32 case(%)	The high dose group N=47 case(%)
Cardiac disorders			
Cardiomegaly	5(13.5)	2(6.3)	7(14.9)
Ear and labyrinth disorders			
Ear pain	1(2.7)	-	-
Eye disorders			
Vision blurred	-	-	1(2.1)
Visual impairment	-	-	1(2.1)
Gastrointestinal disorders			
Abdominal discomfort	-	1(3.1)	-
Abdominal pain	-	-	1(2.1)
Abdominal pain upper	-	-	2(4.3)
Dyspepsia	-	2(6.3)	-
Gastroesophageal reflux disease	-	1(3.1)	-
Stomatitis	1(2.7)	-	-
General disorders and administration site conditions			
Chest discomfort	-	1(3.1)	-
Chest pain	-	-	1(2.1)
Infections and infestations			
Bronchitis	1(2.7)	-	-
Ear infection	-	-	1(2.1)
Lip infection	-	1(3.1)	-
Pneumonia	8(21.6)	9(28.1)	7(14.9)
Investigations			
Alanine aminotransferase increased	1(2.7)	-	-
Aspartate aminotransferase increased	1(2.7)	-	-
Blood calcium decreased	1(2.7)	-	1(2.1)
Blood glucose increased	5(13.5)	1(3.1)	3(6.4)
Blood potassium decreased	1(2.7)	-	-
Blood potassium increased	-	-	1(2.1)
Blood sodium decreased	-	-	1(2.1)
Eosinophil count increased	-	-	2(4.3)
Gamma-glutamyltransferase increased	2(5.4)	-	1(2.1)
Helicobacter test positive	1(2.7)	-	-
Lipase increased	1(2.7)	2(6.3)	2(4.3)
Platelet count decreased	-	-	1(2.1)
Urine ketone body present	-	1(3.1)	-
White blood cell count decreased	2(5.4)	1(3.1)	1(2.1)

Adverse events emerged after treatment	The placebo N=37 case(%)	The low dose group N=32 case(%)	The high dose group N=47 case(%)
White blood cell count increased	-	1(3.1)	1(2.1)
Musculoskeletal and connective tissue disorders			
Arthralgia	-	1(3.1)	-
Back pain	-	1(3.1)	-
Neck pain	-	-	1(2.1)
Pain in extremity	1(2.7)	-	-
Nervous system disorders			
Ageusia	-	1(3.1)	2(4.3)
Anosmia	-	1(3.1)	-
Dizziness	-	1(3.1)	-
Olfactory dysfunction	-	1(3.1)	1(2.1)
Reproductive system and breast disorders			
Dysmenorrhea	-	-	2(4.3)
Vaginal hemorrhage	1(2.7)	-	-
Respiratory, thoracic and mediastinal disorders			
Dysphonia	-	-	1(2.1)
Emphysema	-	-	1(2.1)
Productive cough	1(2.7)	-	-
Pulmonary edema	1(2.7)	-	-
Skin and subcutaneous tissue disorders			
Rash	-	1(3.1)	-
Urticaria	1(2.7)	1(3.1)	3(6.4)

List 2. List of adverse events emerged after treatment by grade

Grade	Adverse events emerged after treatment	The placebo N=37 case(%)	The low dose group N=32 case(%)	The high dose group N=47 case(%)
Mild	Cardiac disorders			
	Cardiomegaly	5(13.5)	2(6.3)	7(14.9)
	Eye disorders			
	Vision blurred	-	-	1(2.1)
	Visual impairment	-	-	1(2.1)
	Gastrointestinal disorders			
	Abdominal pain	-	-	1(2.1)
	Dyspepsia	-	1(3.1)	-
	General disorders and administration site conditions			
	Chest discomfort	-	1(3.1)	-
	Chest pain	-	-	1(2.1)
	Infections and infestations			
	Bronchitis	1(2.7)	-	-
	Pneumonia	8(21.6)	9(28.1)	7(14.9)
	Investigations			
	Alanine aminotransferase increased	1(2.7)	-	-
	Blood calcium decreased	1(2.7)	-	1(2.1)
	Blood glucose increased	5(13.5)	1(3.1)	3(6.4)
	Blood potassium decreased	1(2.7)	-	-
	Blood potassium increased	-	-	1(2.1)
	Blood sodium decreased	-	-	1(2.1)
	Eosinophil count increased	-	-	2(4.3)
	Gamma-glutamyl transferase increased	2(5.4)	-	1(2.1)
	Helicobacter test positive	1(2.7)	-	-
	Lipase increased	1(2.7)	2(6.3)	2(4.3)
	Platelet count decreased	-	-	1(2.1)
	Urine ketone body present	-	1(3.1)	-
	White blood cell count decreased	2(5.4)	1(3.1)	1(2.1)
	White blood cell count increased	.	1(3.1)	1(2.1)
	Nervous system disorders			
	Ageusia	-	1(3.1)	2(4.3)
	Anosmia	-	1(3.1)	-
	Dizziness	-	1(3.1)	-
	Olfactory dysfunction	-	1(3.1)	1(2.1)
	Reproductive system and breast disorders			
	Dysmenorrhea	-	-	1(2.1)

	Vaginal hemorrhage	1(2.7)	-	-
	Respiratory, thoracic and mediastinal disorders			
	Dysphonia	-	-	1(2.1)
	Emphysema	-	-	1(2.1)
	Productive cough	1(2.7)	-	-
	Pulmonary edema	1(2.7)	-	-
	Skin and subcutaneous tissue disorders			
	Rash	-	1(3.1)	-
	Urticaria	1(2.7)	1(3.1)	1(2.1)
Moderate	Ear and labyrinth disorders			
	Ear pain	1(2.7)	-	-
	Gastrointestinal disorders			
	Abdominal discomfort	-	1(3.1)	-
	Abdominal pain upper	-	-	2(4.3)
	Dyspepsia	-	1(3.1)	-
	Gastroesophageal reflux disease	-	1(3.1)	-
	Infections and infestations			
	Ear infection	-	-	1(2.1)
	Lip infection	-	1(3.1)	-
	Investigations			
	Aspartate aminotransferase increased	1(2.7)	-	-
	Musculoskeletal and connective tissue disorders			
	Arthralgia	-	1(3.1)	-
	Back pain	-	1(3.1)	-
	Neck pain	-	-	1(2.1)
	Pain in extremity	1(2.7)	-	-
	Reproductive system and breast disorders			
	Dysmenorrhea	-	-	1(2.1)
	Skin and subcutaneous tissue disorders			
	Urticaria	-	-	2(4.3)
	Vascular disorders			
	Hypertension	1(2.7)	1(3.1)	1(2.1)

List 3. List of adverse events emerged after treatment – considered to be related to drug or the placebo

Related to drug or the placebo	List of adverse events emerged after treatment	The placebo N=37 case(%)	The low dose group N=32 case(%)	The high dose group N=47 case(%)
Considered to be related to drug or the placebo	Ear and labyrinth disorders			
	Ear pain	1(2.7)	-	-
	Eye disorders			
	Vision blurred	-	-	1(2.1)
	Gastrointestinal disorders			
	Gastroesophageal reflux disease	-	1(3.1)	-
	Stomatitis	1(2.7)	-	-
	General disorders and administration site conditions			
	Chest pain	-	-	1(2.1)
	Infections and infestations			
	Pneumonia	1(2.7)	1(3.1)	-
	Investigations			
	Alanine aminotransferase increased	1(2.7)	-	-
	Aspartate aminotransferase increased	1(2.7)	-	-
	Blood calcium decreased	1(2.7)	-	1(2.1)
	Blood glucose increased	5(13.5)	1(3.1)	3(6.4)
	Blood potassium decreased	1(2.7)	-	-
	Blood potassium increased	-	-	1(2.1)
	Blood sodium decreased	-	-	1(2.1)
	Eosinophil count increased	-	-	2(4.3)
	Gamma-glutamyl transferase increased	1(2.7)	-	1(2.1)
	Lipase increased	1(2.7)	2(6.3)	2(4.3)
	Platelet count decreased	-	-	1(2.1)
	Urine ketone body present	-	1(3.1)	-
	White blood cell count decreased	2(5.4)	1(3.1)	1(2.1)
	White blood cell count increased	.	1(3.1)	1(2.1)
	Musculoskeletal and connective tissue disorders			
	Arthralgia	-	1(3.1)	-
	Pain in extremity	1(2.7)	-	-
	Nervous system disorders			
	Ageusia	-	-	1(2.1)
	Dizziness	-	1(3.1)	.
	Reproductive system and breast disorders			
	Vaginal hemorrhage	1(2.7)	-	-
	Respiratory, thoracic and mediastinal disorders			

Related to drug or the placebo	List of adverse events emerged after treatment	The placebo N=37 case(%)	The low dose group N=32 case(%)	The high dose group N=47 case(%)
	Dysphonia	-	-	1(2.1)
	Skin and subcutaneous tissue disorders			
	Rash	-	1(3.1)	-
	Urticaria	1(2.7)	1(3.1)	3(6.4)
	Vascular disorders			
	Hypertension	-	-	1(2.1)
Not considered to be related to drug or the placebo	Cardiac disorders			
	Cardiomegaly	5(13.5)	2(6.3)	7(14.9)
	Eye disorders			
	Visual impairment	-	-	1(2.1)
	Gastrointestinal disorders			
	Abdominal discomfort	-	1(3.1)	-
	Abdominal pain	-	-	1(2.1)
	Abdominal pain upper	-	-	2(4.3)
	Dyspepsia	-	2(6.3)	-
	General disorders and administration site conditions			
	Chest discomfort	-	1(3.1)	-
	Infections and infestations			
	Bronchitis	1(2.7)	-	-
	Ear infection	-	-	1(2.1)
	Lip infection	-	1(3.1)	-
	Pneumonia	7(18.9)	8(25.0)	7(14.9)
	Investigations			
	Gamma-glutamyl transferase increased	1(2.7)	-	-
	Helicobacter test positive	1(2.7)	-	-
	Musculoskeletal and connective tissue disorders			
	Back pain	-	1(3.1)	-
	Neck pain	-	-	1(2.1)
	Nervous system disorders			
	Ageusia	-	1(3.1)	1(2.1)
	Anosmia	-	1(3.1)	-
	Olfactory dysfunction	-	1(3.1)	1(2.1)
	Reproductive system and breast disorders			
	Dysmenorrhea	-	-	2(4.3)
	Respiratory, thoracic and mediastinal disorders			
	Emphysema	-	-	1(2.1)

Related to drug or the placebo	List of adverse events emerged after treatment	The placebo N=37 case(%)	The low dose group N=32 case(%)	The high dose group N=47 case(%)
	Productive cough	1(2.7)	-	-
	Pulmonary edema	1(2.7)	-	-
	Vascular disorders			
	Hypertension	1(2.7)	1(3.1)	-

Supplementary Methods

CP-COV03 synthesis by nanohybrid technology via solid-liquid interface activating method for clinical trials

The CP-COV03 capsule was manufactured in a GMP facility (YOOYOUNG Pharm.). Niclosamide (2,400 g), magnesium oxide (MgO) (1,680 g), and hydroxy propyl methyl cellulose (HPMC) (600 g) were weighed and mixed manually. Next, 195 g of the mixture was weighed and mixed using a ball mill machine (Retsch, PM100) at 170 rpm for 10 minutes. The prepared mixture solution (20.8 g) containing 419.95 g ethanol (95.0%) and 277.76 g water was added to the mixture, and five rounds of milling were conducted using a ball mill machine at 170 rpm for 2 minutes each. Thereafter, the materials were milled at 170 rpm for 10 minutes, repeating the milling process four times. The resulting nanohybrid product was dried in vacuum dryer at 45 °C for at least 1 hour, and the dried nanohybrid was capsuled to get the CP-COV03 (quantity of niclosamide is 50 mg per capsule).

PXRD, NMR study for niclosamide and FT-IR analysis on the deuterated one w.r.t pH

Powder X-ray diffraction characterizations (PXRD) were performed with a Bruker D2 phase diffractometer (Bruker, Karlsruhe, Germany) equipped with Cu Kα radiation (λ = 1.5418 Å) to examine phase formation. The data were recorded with a voltage of 30 kV and a current of 10 mA.

For pH dependent deprotonation studies, a total of 360 mg of niclosamide was added into a 100 mL 3-neck flask, and 72 mL of 70% ethanol (EtOH) (70% EtOH + 30% H2O) (5 mg/mL) was added; 0.5 M NaOH was added to increase pH until niclosamide dissolved fully at pH 13.5 ± 0.1 and was stirred for 1 h. The solution was separated and dried under vacuum RT for 17 h. Next, 0.5 M HCl was added to decrease the pH to neutral (pH 7.0 ± 0.1) with 1-h stirring; the solution was separated and dried under

vacuum RT for 17 h. Further, 0.5 M HCl was added to acidify the pH (pH 3.0 ± 0.1) with 1-h stirring; the solution was then separated and dried under vacuum RT for 17 h. The pH adjustment process was divided into three cycles with the same dispersed solution; the basic, neutral, and acidic solutions were separated at each step and dried under vacuum RT for 17 h. The proton NMR measurements were analyzed using the Bruker/Magnet System500'54 Ascend at Dankook University, Cheonan, South Korea, and D6-DMSO (Duetero.de, Sejong-si, 30128, South Korea). The FT-IR studies were performed using a Jasco FT-IR-6100 spectrometer (JASCO, Tokyo, Japan) via the standard KBr disk method in transmission mode (spectral range 4000–400 cm $^{-1}$, resolution 1 cm $^{-1}$, 40 scans per spectrum).

Deuteration reaction of niclosamide for FT-IR

The pH-dependent deprotonation of niclosamide was conducted as follows. The O-D stretching vibration mode of niclosamide was analyzed with respect to pH. First, a niclosamide solution was prepared above pH 10; D2O was added to this solution and titrated with acid to bring it below pH 7 for crystallization. Dissolved crystals of niclosamide were prepared via base titration. The O-D and O-H stretching vibrations in each step were detected to enhance the solubility related to the deprotonation of niclosamide, resulting in bonding between positively charged MgO particles and negatively charged niclosamide molecules.

For FT-IR, approximately 12 mg of the sample was mixed with 100 mg of KBr pellets and thoroughly ground using a mortar and pestle. The obtained samples were pelletized using a hydraulic press machine at 15–20 MPa for 1 min and then used for FT-IR characterization as described above.

BET analysis

The porous parameters were calculated using the Brunauer–Emmett–Teller (BET) technique at a relative pressure (P/P_0) range of 0.05–0.25, and the total pore volume was measured at a relative pressure of 0.99. N2 adsorption-desorption measurements were conducted on a BELSORP II mini (Japan) machine, at 77 K. The porous parameters were calculated using the Brunauer-Emmett-Teller (BET) technique at relative pressure ranges of 0.05–0.25, and the total pore volume was measured at

elative pressure of 0.99. All samples were pretreated by degassing at 373 K for 4.5 h in vacuum (10^{-2} kPa) to remove all moisture and gaseous elements remaining in the pores.

SEM, HAADF-STEM, HR-TEM, and EDS analyses

Field emission scanning electron microscopy (Hitachi SU8220) analyses used to identify morphological transformations of MgO and their composite forms. For sample preparation, MgO, NIC-MgO, and NIC-MgO-HPMC powder were placed on the carbon tape, and Pt coating (20 mA, 1 minute) was done. HR-TEM, HAADF-STEM and EDS mapping were recorded on JEM-F200 with a Schottky field emission gun operated with an accelerating voltage of 200 kV. This mapping was performed on various samples such as MgO, NIC-MgO, and NIC-MgO-HPMC to analyze the crystal lattice structure. The samples were prepared by drying a drop of samples in D.W. on the surface of a carbon-coated copper grid and dried for 30 min. During measurement, the vacuum was maintained at an average 1×10^{6} Pa with slight changes depending on the samples, with an electron beam current of 123 μA. For HAADF-STEM image acquisition, the camera length on STEM was 120 mm and the acquisition time of HAADF images was 19 μs per pixel. The resultant images were analyzed using Gatan software.

Zeta potential

MgO powder was ground in 70% EtOH to mimic the synthetic conditions for niclosamide-MgO and niclosamide-MgO-HPMC. The ground MgO powder was then used to measure the surface charge of MgO. For this, 70% EtOH was added to a vial containing ground MgO powder (0.5 mg/mL), stirred for 24 h, and centrifuged at 3600 rpm for 10 min. The supernatant was then separated from the precipitate, and a new 70% EtOH solvent (pH = 9.4) was added. The final sample was measured in a disposable zeta cell, and the average value from five independent experiments was used as the final surface charge (Table S6). Zeta potential analyses were performed using DLS/Zeta EL-SZ-2000 instrument (Otsuka Electronics, Japan) with disposable square cuvettes in distilled water.

X-ray photoelectron spectroscopy (XPS)

XPS was performed with a monochromatic Al X-ray source (K-alpha+, Thermo scientific, UK), employing K-alpha+ monochromated radiation (1486.7 eV) at 50 eV analyzer pass energy. Ar etching (1,000 eV, 10 sec) was done to remove dust or surface contaminants. The pressure in the analysis chamber was maintained at 5.0×10^{-9} Torr. Spectral fitting was performed using Origin 2018 and binding energies were referenced to adventitious carbon at 284.6 eV, with surface atomic compositions calculated via correction for the appropriate instrument response factors. FWHM and peak positions were fixed across each adsorption series, and the minimum number of peaks required to achieve good fit was used in all cases. Samples were measured as solid powder form attached to the tape.

PARTICIPANTS

A screening test was conducted for those who provided written consent to participate, and a baseline test was conducted for those who met the eligibility criteria. The enrolled participants received study medications and completed the trial schedule. They were randomly assigned to the CP-COV03 with the low dose group, the high dose group, and the placebo groups in a 1:1:1 ratio. Details of the trial schedule are provided in the Supplementary Appendix (Appendix pp. 14–16).

Three of the seventeen screening failures and six of the nine early cessation of participations declined to participate in the study, withdrawing their consent prior to randomization. Although the screening failure and early cessation of participation documentation did not specify the reasons for the consent withdrawals, it is suspected that most were likely related to hospitalization. 97.0% (291 out of 300) of participants completed the three additional follow-up visits.

For reference, not all participants were discharged on day 6 regardless of their symptoms. According to the protocol, the hospital discharge date could be extended based on symptoms or in accordance with the Korea Disease Control and Prevention Agency (KDCA) guidelines for COVID-19.

Briefly, each participant took nine capsules three times daily for 5 days, beginning from the evening of Day 1 to the afternoon of Day 6 (5 days). The investigational products were administered at

approximately 8-hour intervals while on an empty stomach. Body temperature and oxygen saturation were measured three times per day or more, if necessary. Blood pressure, heart rate, and respiratory rate were measured daily. After the first two doses, symptoms were evaluated using a 4-point system (0=absent, 1=mild, 2=moderate, 3=severe) for 12 symptoms (fever, cough, sore throat, headache, muscle pain, chills/shivering, runny nose/nasal congestion, fatigue/malaise, shortness of breath, nausea/vomiting, diarrhea) from the morning of Day 2; symptoms were evaluated by researchers in person or over the phone every morning and evening. The symptoms were scored based on the time when they were the worst in the past 12 hours by referring to the criteria. Laboratory tests (hematologic tests including WBC, RBC, hemoglobin, hematocrit, platelet count; blood chemistry tests including glucose, BUN, uric acid, total protein, albumin, total bilirubin, alkaline phosphatase, AST, ALT, γ-GT, LDH, creatinine, CPK, Na, K, Cl, calcium, phosphorus, eGFR, CRP, lipase, amylase; blood coagulation tests including PT, PT-INR (calculated), PT (%), and APTT; urine tests including specific gravity, pH, protein, glucose, ketones, color, leukocytes, bilirubin, occult blood, urobilinogen, and nitrite; and serologic tests including HBsAg, anti-HCV Ab, HIV Ag/Ab or HIV Ab, and Syphilis Reagin test) and chest radiography were performed at baseline and on Day 6 and were additionally performed if necessary. Samples for viral load measurements were collected on days 0, 2, 4, 6, and 8.

Blood samples for pharmacokinetic analysis were collected from 60 volunteers. It involved sampling two times on Day 1 (before and 3 hours after evening administration), four times on Days 2 and 6 (before and 3 hours after morning and noon administration), two times on Days 3 and 4 (before and 3 hours after noon administration), and four times on Day 5 (before and 3 hours after noon and evening administration).

ASSESSMENT

The primary endpoint of the trial was to assess the efficacy of CP-COV03 as compared with the placebo. Efficacy was assessed based on the number of days required for all 12 COVID-19 symptoms to improve and be sustained for more than 48 hours until Day 14. Cases in which symptoms of COVID-19 were assessed were defined as improved according to the following conditions: 1) if symptoms observed at

baseline as 2 points or more improved to 1 point or less; 2) if a symptom observed as 1 point at baseline improved to 0 points; and 3) if symptoms that were not observed at baseline but newly occurred during the clinical trial improved to 0 again. For participants whose symptoms did not improve until Day 14.5 or those whose condition was not sustained for more than 48 hours after symptom improvement until Day 14.5, the number of days required for symptom improvement was calculated as 13, i.e., the maximum value that could appear in the analysis, and classified as censored.

The secondary outcomes were the safety and tolerability of CP-COV03. A safety analysis was performed on the safety analysis set. For all patients who received the study medication, all adverse reactions were tabulated, comprehensively reviewed, and graded by the study clinician for severity (mild, moderate, or severe) and relatedness to the study medication.

Additional analyses were performed to evaluate the change in the SARS-CoV-2 viral load, which was measured using qPCR on days 0, 2, 4, 6, and 8. The pharmacokinetics of CP-COV03 at steady state after repeated administration were assessed, and the correlation between the blood concentration of CP-COV03 and the SARS-CoV-2 viral load was also evaluated.

STATISTICAL ANALYSIS

This trial was conducted with 300 participants (100 in each group), which was the number of participants required to evaluate the efficacy and safety of the study medication. At the time of planning this clinical trial, data on the progression to severe COVID-19 infection was available, but there was no reference data on symptom improvement. Therefore, we internally calculated the sample size for the clinical trial by referring to the symptom improvement effect of oseltamivir in influenza infection. For reference, Paxlovid's sample size calculation was also based on the clinical trial (BLAZE-1) of the antibody treatment Bamlanivimab (Pfizer, EPIC-HR protocol, protocol number: C4671005). According to Table 2 from Tamiflu studies[1], the duration of symptoms in the placebo group (129 participants) was a mean of 103.3 hours, with a 95% confidence interval of 92.6–118.7, while in the treatment group (121 participants), the mean was 69.9 hours, with a 95% confidence interval of 60.0–87.9. Based on these results, the standard deviations were calculated as approximately 75.62 hours for the placebo group

and 78.29 hours for the treatment group. The effect size (Cohen's d) was approximately 0.434. Considering a significance level of 5% and a power of 90%, the required sample size for the clinical trial was calculated to be 92 participants per group. Therefore, setting the sample size for this clinical trial at 100 participants per group is appropriate.

All participants who received at least one dose of the study medication were included in the ITT population. The PPS population comprised participants in the ITT population, except those with protocol violations that could have affected the assessment of antiviral activity and symptom improvement. To evaluate CP-COV03's effectiveness in a similar method to existing COVID-19 antivirals, ad-hoc analyses for symptom improvement were performed which included the application of Cox proportional hazards regression model with Efron method referencing Molnupiravir's MOVe-OUT trial protocol. Additionally, for accurate symptom evaluation among individuals receiving concomitant medications, symptom evaluation was censored the day before concomitant drug administration. Thus, additional analyses were performed for individuals who received medication within 3 days of symptom onset like the efficacy evaluation performed for Nirmatrelvir and Ensitrelvir, to further evaluate efficacy. Participants treated within 3 days after symptom onset were defined as the mITT-1[2]. Among the mITT population, additional efficacy analyses were performed on the participants after excluding those who tested negative on or missed the RT-PCR baseline test (mITT-2).

In this clinical trial, the primary endpoint of efficacy was assessed in the ITT, mITT (mITT-1 and mITT-2), and PPS populations. A safety analysis was performed for all participants who received at least one dose of the study medication.

For the primary efficacy endpoint, the number of days required for the COVID-19 symptoms to improve and be sustained for more than 48 hours is presented in descriptive statistics for each group. Using the Cox proportional hazards regression model with the administration group as a factor and age and disease severity as covariates, the hazard ratios (HRs) of the administration groups, 95% confidence intervals (CIs), and *P*-values were calculated. As CP-COV03 contains magnesium oxide[3], we performed a separate efficacy analysis on the improvement of three COVID-19 symptoms (fever, headache, and sore throat), excluding gastrointestinal symptoms such as diarrhea. Descriptive statistics were presented for

the change in SARS-CoV-2 viral load by administration group, and comparisons between administration groups were analyzed using an analysis of covariance (ANCOVA) model with the administration group as a factor and baseline value and stratification factors (age and disease severity) as covariates. According to the pre-specified secondary endpoint in the clinical trial protocol, the number of days required for each targeted COVID-19 symptom to improve and be maintained for more than 48 hours until Day 14 was analyzed using an ANCOVA model, with the treatment group as a factor and baseline values and stratification factors (age, severity) as covariates.

The pharmacokinetics of CP-COV03 at a steady state after repeated administration were presented descriptively by the administration group. The correlation between the area under the curve (AUC) for time-blood concentration and viral load was evaluated using Pearson's correlation coefficient.

For the safety assessment, all adverse reactions in this trial were standardized using the System Organ Class (SOC) and Preferred Term (PT) classifications based on the latest version of MedDRA[4]. The number of participants, number of cases, and percentage of adverse reactions were presented based on the SOC and PT according to the administration group and were also presented separately by severity. Differences in the incidence of adverse and serious adverse events between the administration groups were analyzed using the chi-square test or Fisher's exact test. Statistical significance was set at $P<0.05$. All statistical analyses were performed using SAS v.9.4 (SAS Institute Inc., Cary, NC, USA).

Supplementary Note-1

CP-COV03 based on inorganic base nanohybrid technology.

For clinical trials, the CP-COV03 samples were prepared by the solid-liquid interface activating method, namely ball-milling niclosamide with MgO solid-base in a solution of 70% ethanol and 30% water in GMP facility.

MgO is a solid base with a positive surface charge of +35.04 ± 2.34 mV (Figure S2.B, Table S6), which can induce the deprotonation of niclosamide on the MgO surface under basic conditions, generatingan overall negative charge on the niclosamide (Figure S2C, D) and eventually enhancing its solubility. Thus, generated anionic niclosamide can bind on the cationic MgO surface via a preferential

cation-anion bonding as evidenced by NMR analyses. The pH-dependent deprotonation behavior of niclosamide was analyzed by nuclear magnetic resonance (NMR) and Fourier transform infrared (FT-IR) spectroscopy (Figure S2D, E and Figure S3, S4, Table S7). Both amide and phenolic protons at 11.3 ppm and 12.7 ppm disappeared in niclosamide as pH reached approximately 13.5 at which point it became completely soluble (Figure S4). The NMR analysis was further validated using FT-IR analysis. Consequently, deuterated niclosamide was formed, and characteristic –OD stretching at ~ 2400 cm^{-1} [5] was observed under basic conditions (pH ~13.5) (Figure S2D). Additionally, the pH-dependent deprotonation of niclosamide was determined to be reversible, as evidenced by the pH variations from basic (pH 13.5) to acidic (pH 3) under several cycles (Figure S2C, D). Deprotonated niclosamide was recovered in cycles 2 and 3. Upon gradual reduction of the pH to acidic conditions, the protons of phenol and amide disappeared. We aimed to confirm this phenomenon using infrared spectroscopy under the synthetic condition with 70% EtOH. However, perfect differences in -OH and $-NH_2$ infrared frequencies were limited because the peaks were broad and overlapped with other complicated peaks (Figure S5B). This is the main reason for analyzing the pH-dependent deprotonation of deuterated niclosamide under the conditions with cycles 1–3 to observe the O-D stretching, which is distinguishable (Figure S2D, Figure S4). The bands at 3580 and 3484 cm^{-1} in the intact niclosamide were due to the -OH and N–H stretching vibrations in the niclosamide[3]. Also, niclosamide shows their characteristic peaks in aromatic =C-H at 3092 cm^{-1}, C=O at 1681 cm^{-1}, aromatic -C-C- at 1607 cm^{-1}, amide -NH bending and C-N at 1563 cm^{-1}, and -OH bending and -N-O at 1339 cm^{-1} in Figure S6 and Table S7[6-9]. Figure S2D represents a magnified view (2700–2300 cm^{-1}) of the FT-IR spectra of the deuterated niclosamide in 70% EtOH solution (with 30% D_2O), NaOD, and DCl for adjusting pH. As shown in Figure S2D, the peaks at 2633, 2570, and 2534 cm^{-1} correspond to the O-D stretching observed in EtOD and D_2O solvent[10-13]. The transition of pH from basic to acidic resulted in an increase of peak intensity at 2400 and 2354 cm^{-1}, due to the formation of O-D and N-D resulting from the transformation of niclosamide's phenolic -OH and –NH moieties, respectively[9,14,15]. When the pH becomes basic, the hydroxyl group (–OH) undergoes deprotonation, resulting in a negative charge. If the pH subsequently decreases, deuterium is introduced into the site previously occupied by hydrogen. This deuteration process enabled the distinct observation

of the above-mentioned O-D and N-D peaks.

This validates the nano-hybridization via cation-anion interaction between MgO and niclosamide in CP-COV03. The spherical morphology (~200 nm size) of MgO was demonstrated by high-angle annular dark field-scanning transmission analysis (HAADF-STEM) and corresponding HR-TEM were analyzed (Figure S7). In addition, the crystal structure of the MgO NPs was determined using high-resolution transmission electron microscopy (HRTEM) and high-angle annular dark-field imaging (Figure S7). This observation is consistent with the results obtained from the XRD analysis (Figure S8.A). The observed interplanar distances between the lattice fringes were 0.211 and 0.153 nm, corresponding to the (200) and (220) planes of MgO, respectively[16]. The MgO(200) surface has the lowest surface energy in ionic rock salt structure, while other facets such as MgO(111) and MgO(220) are unstable polar surfaces[17]. The (111) and (220) polar facets were predominant in hybrid samples such as NIC-MgO and NIC-MgO-HPMC as evidenced through FFT analysis. Thus, it is possible to have a polar-polar interaction among niclosamide and MgO (220) facets in addition to their ionic bonding in nanohybrid samples.

As observed through HR-TEM imaging and HAADF-STEM, the edge behavior of MgO changed after hybridization with niclosamide and HPMC with more amorphous nature (Figure S7C,E) owing to the predominant ionic or polar-polar bound niclosamide and hydrogen-bonded HPMC, which are consistent with the NMR, FTIR, and XPS results (Figure S2, S8.B).

The specific surface area measured by the BET analysis (Figure S9) confirms that the present MgO had neither micropores nor mesopores (Figure S8). Further, the niclosamide-MgO formulation was coated with hydroxy propyl methyl cellulose (HPMC) (Figure S2A) to form niclosamide-MgO-HPMC (CP-COV03). The HPMC coating ensures the controlled release of niclosamide in the gastrointestinal system[3] along with improved mucosal adhesion and intestinal permeability, thereby enhancing the overall bioavailability of CP-COV03.

Additional EDX experiments (Figure S10) confirmed the presence of the major elements expected in the hybrid niclosamide-MgO (Figure S10) and niclosamide-MgO-HPMC, indicating that hybridization was successful.

XPS was utilized to understand the changes in binding energy of Mg, O, C and N within the hybrids compared to pristine MgO (Figure S8.B). Compared to that for pristine MgO, the O1s scan indicated differences in binding energy for MgO with its hybrid forms. The O1s scan confirmed that MgO has two distinct peaks at 531.5 (OH/CO_3^{2-}) and 529.6 eV for O^{2-} [18] and that for NIC-MgO are 531.5 and 529.8 eV, respectively, with significant reduction in intensity and are almost similar with NIC-MgO-HPMC as well. Such a drastic XPS O*1s* intensity reduction of MgO upon hybridization indicates that the bonding could be due to the ionic interaction between positive surface charge of MgO and negative charge of deprotonated NIC. The deprotonated NIC showed an XPS O*1s* scan at ~532.6 eV, which was maintained even after coating with HPMC (NIC-MgO-HPMC).

The atomic % for N and C for NIC-MgO-HPMC further validated the proper coating on NIC-MgO as shown in Table S8. In pristine MgO, the atomic % for N*1s* is negligible (3.12%), where it is presented in NIC-MgO having 6.50 and 5.80 % for NIC-MgO-HPMC samples. Similarly, C*1s* atomic % was enhanced after coating HPMC on NIC-MgO (Table S8).

Through our formulation studies, we have discovered that modulating the quantity of HPMC allows fine-tuning of the PK profile, including Cmax and the duration of niclosamide plasma concentration. Due to the mucoadhesive property of HPMC, its quantity increases lead to the prolongation of the duration of niclosamide plasma concentration while reducing Cmax. Based on this finding, we have designed a formulation to be used in clinical trials aimed at maintaining appropriate blood concentrations when administered (Figure S11). The PK profile in Figure 2b shows the modulated PK profile for COVID-19 treatment by the designed formulation of CP-COV03. Within the initial 24 hours after the first CP-COV03 administration, the high NIC plasma concentration was observed. In the later phase, stable NIC plasma concentration was observed.

Supplementary Figures

A

Subject	Baseline	Day 2	2.5	3	3.5	4	4.5	5	5.5	6	6.5	7	7.5	8	8.5	9	9.5	10	10.5	11	11.5	12	12.5	13	13.5	14	14.5	Symptom improvement date
S0XXXX	1	1	1	1	1	0	0	0	0	0	0	0	0	0	0	0	0	0	0	0	0	0	0	0	0	0	0	6

Symptom improvement (Day 4)

48hr after symptom improvement (Day 6)

B

Subject	Baseline	Day 2	2.5	3	3.5	4	4.5	5	5.5	6	6.5	7	7.5	8	8.5	9	9.5	10	10.5	11	11.5	12	12.5	13	13.5	14	14.5	Symptom improvement date
S0XXXX	1	1	1	0	0	0	0	0	0	0	0	1	0	0	0	0	0	0	0	0	0	0	0	0	0	0	0	9.5

Symptom improvement (Day 3)

Symptom recurrence (Day 7)

Symptom improvement (Day 7.5)

48hr after symptom improvement (Day 9.5)

Figure S1. Example of symptom improvement date evaluation. A) On Day 4, the symptom score decreased from 1 to 0 and was sustained for 48 hours, indicating sustained symptom improvement. The Day 6 was recorded as the symptom improvement date. B) On Day 3, the symptom score decreased from 1 to 0 and was sustained for 48 hours, indicating sustained symptom improvement; however, symptom recurrence was observed on Day 7. The last date the recurred symptom improved was Day 7.5 and was sustained for 48 hours, resulting in Day 9.5 being recorded as the symptom improvement date.

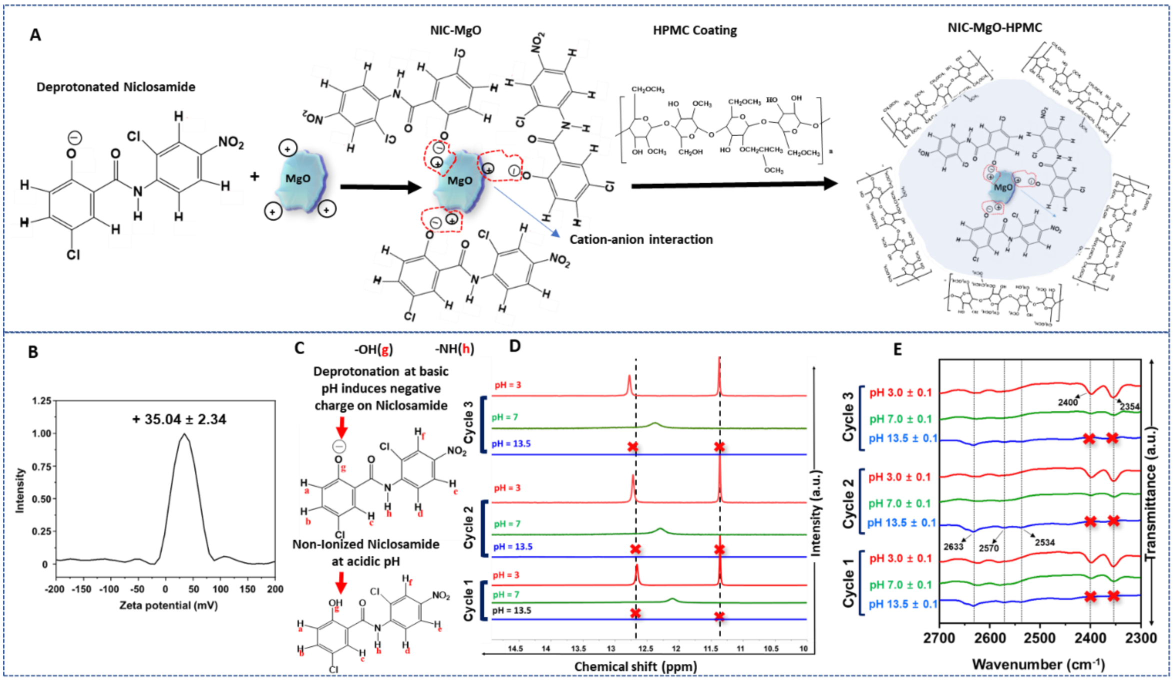

Fig. S2. Nano-hybrid formulation to enhance the bioavailability of niclosamide. A) Nanohybridization technology for CP-COV03 formulation via niclosamide, magnesium oxide (MgO) and hydroxy propyl methyl cellulose (HPMC); B) zeta potential for pristine MgO (grinded in 70% ethanol); C) pH-dependent deprotonation behavior of niclosamide and evidenced by proton NMR spectroscopy D) under various conditions from cycles 1 to 3 (cycle 1, basic to acidic; cycle 2, first cycle solution was directly adjusted to basic (pH 13.5), neutral (pH 7), and acidic (pH 3) conditions; and cycle 3, second cycle 2 solution was directly adjusted to basic, neutral, and acidic pH variations); E) corresponding Fourier transform infrared (FT-IR) spectra with deuterated niclosamide

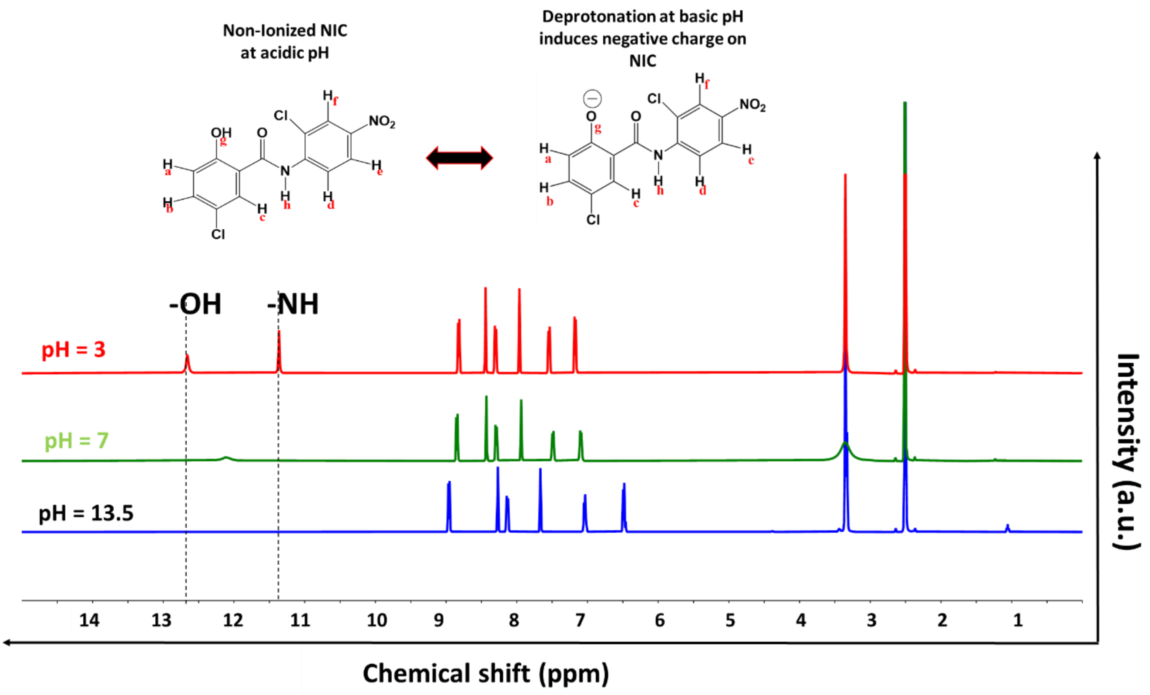

Figure S3. Full proton NMR spectra for the pH dependency of niclosamide evidenced by the disappearance of phenolic proton by NMR spectroscopy under various pH conditions (pH 13.5, 7, and 3).

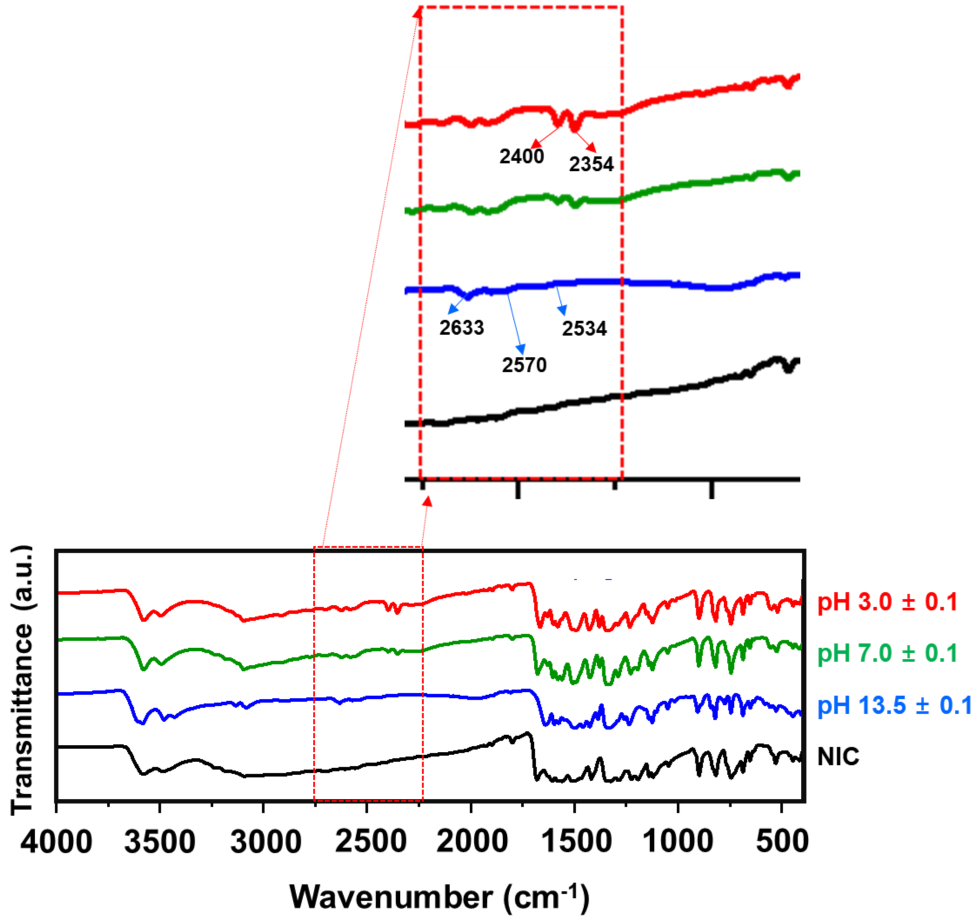

Figure S4. Full-spectra for Fourier transform infrared analysis on deprotonation behavior of deuterated niclosamide under various pH conditions, such as acidic, neutral, and basic (pH 3, 7, 13.5, respectively).

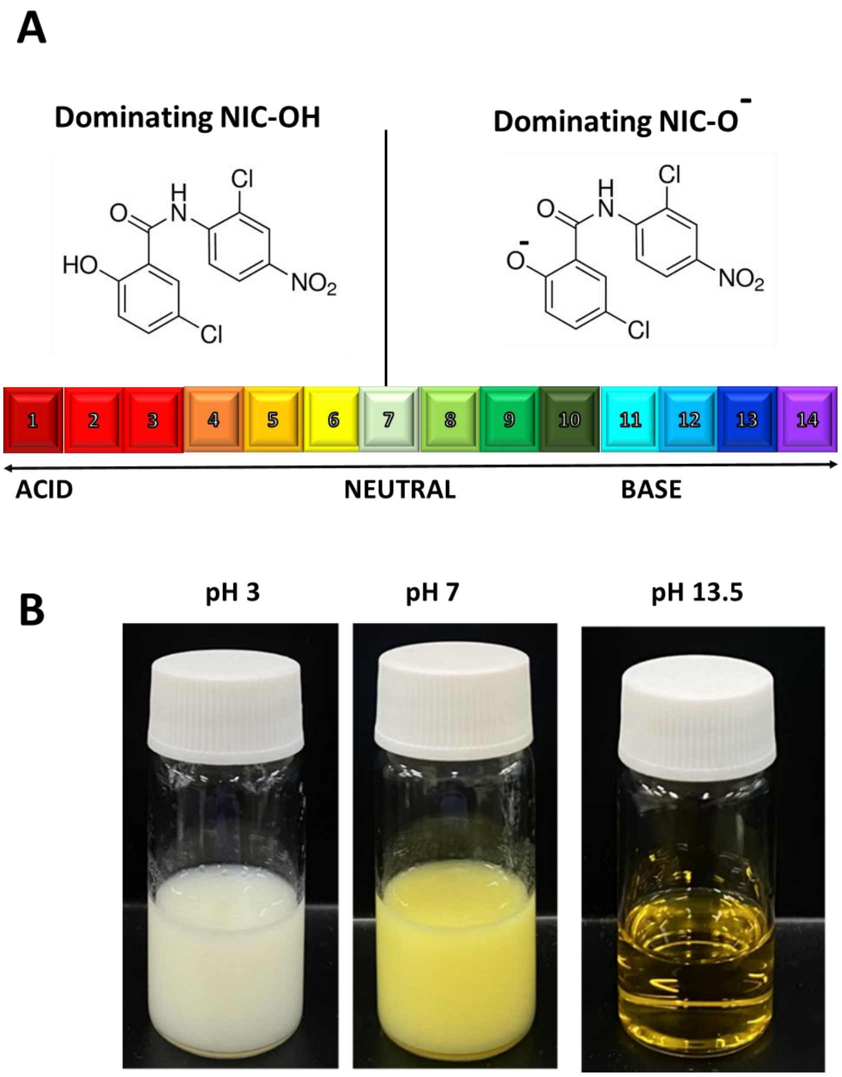

Figure S5. pH dependence of niclosamide. A) Below neutral pH, niclosamide could be in its insoluble crystal form, whereas under basic conditions, its deprotonated form is dominant. B) pH-dependent solubility behavior of niclosamide under various pH conditions such as acidic (**pH 3),** neutral (pH **7),** and basic **(pH 13.5)**.

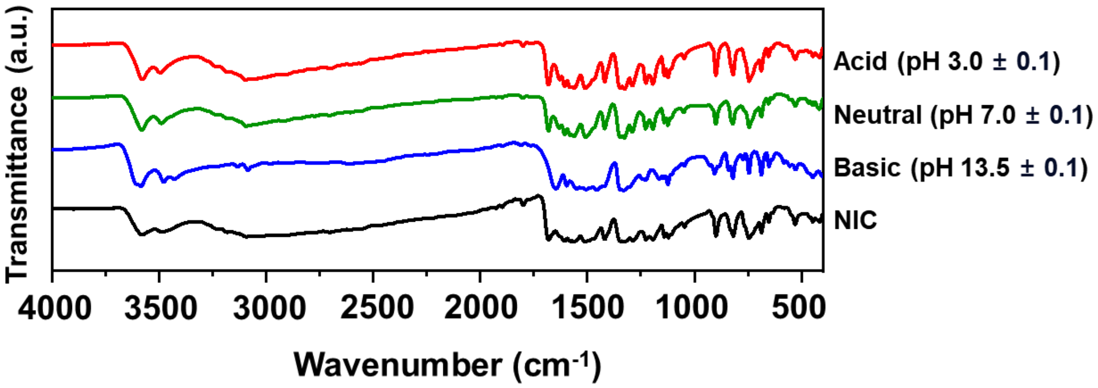

Figure S6. Fourier transform infrared spectra for niclosamide under various pH conditions, such as acidic, neutral, and basic (pH 3, 7, 13.5, respectively).

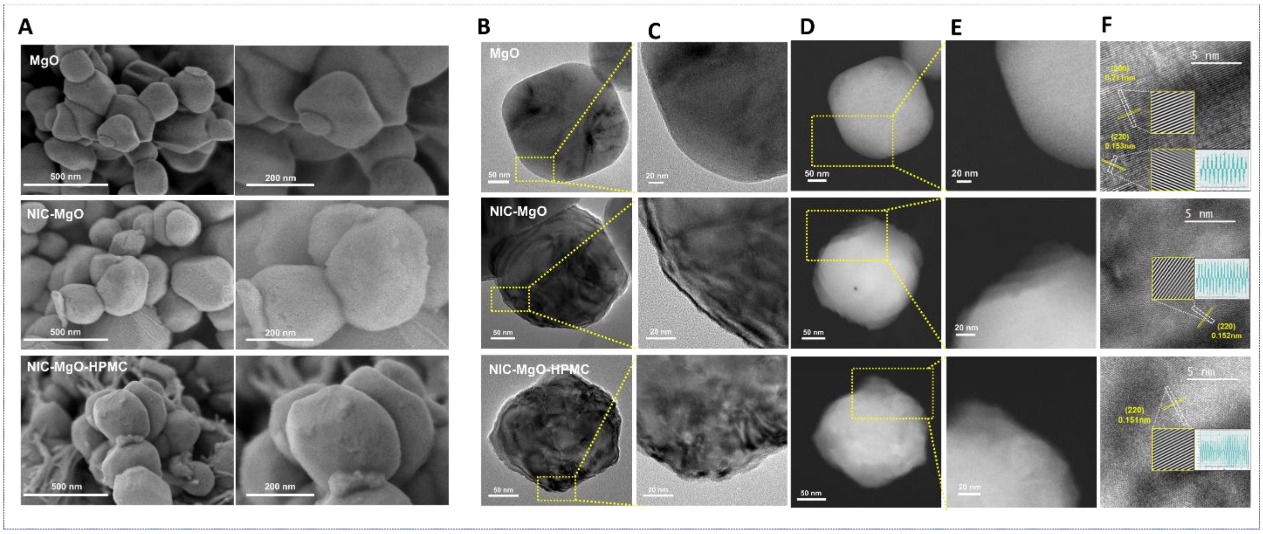

Figure S7. A) Scanning electron microscope (SEM) analysis B) high resolution tunneling electron microscopy (HR-TEM); C) corresponding magnified version showing edge behavior for MgO, NIC-MgO and NIC-MgO-HPMC samples; D-E) High-angle annular dark field-scanning transmission (HAADF-STEM) analysis and their corresponding magnified version for edge behavior for the same samples; F) inverse fast-Fourier-transform (FFT); magnified inverse FFT showing the selected region (white color box) for line profile, and line-profile; image for the selected box in its inverse FFT image indicating its d spacing of MgO. Photometric intensity profiles are indicated along the yellow dashed lines.

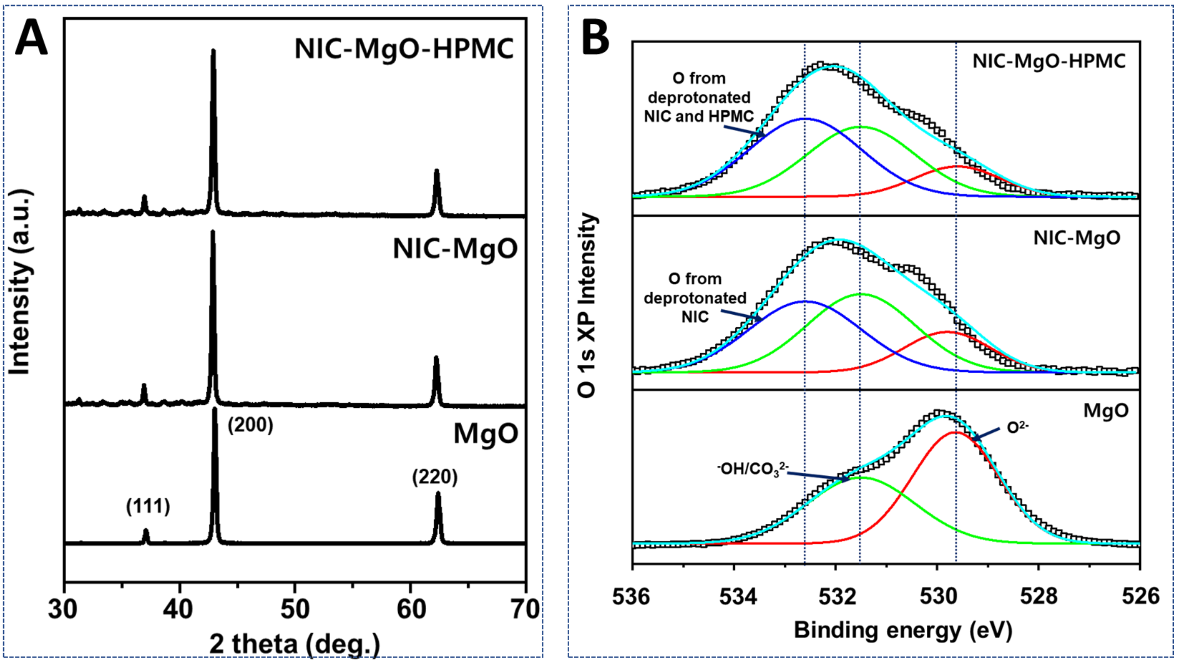

Figure S8. A) PXRD and B) XPS analyses for pristine MgO, NIC-MgO, and NIC-MgO-HPMC.

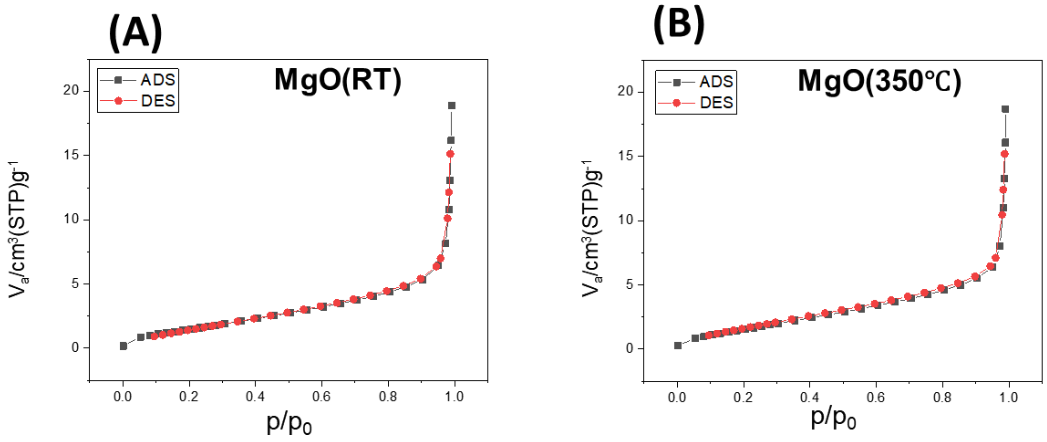

S_{BET} [m^2 g^{-1}]	6.64
V_p (cm^{3}g^{-1})	0.028

S_{BET} [m^2 g^{-1}]	7.23
V_p (cm^{3}g^{-1})	0.029

Figure S9. Surface properties by BET analysis for A) MgO (Heavy) and B) MgO at 350 °C.

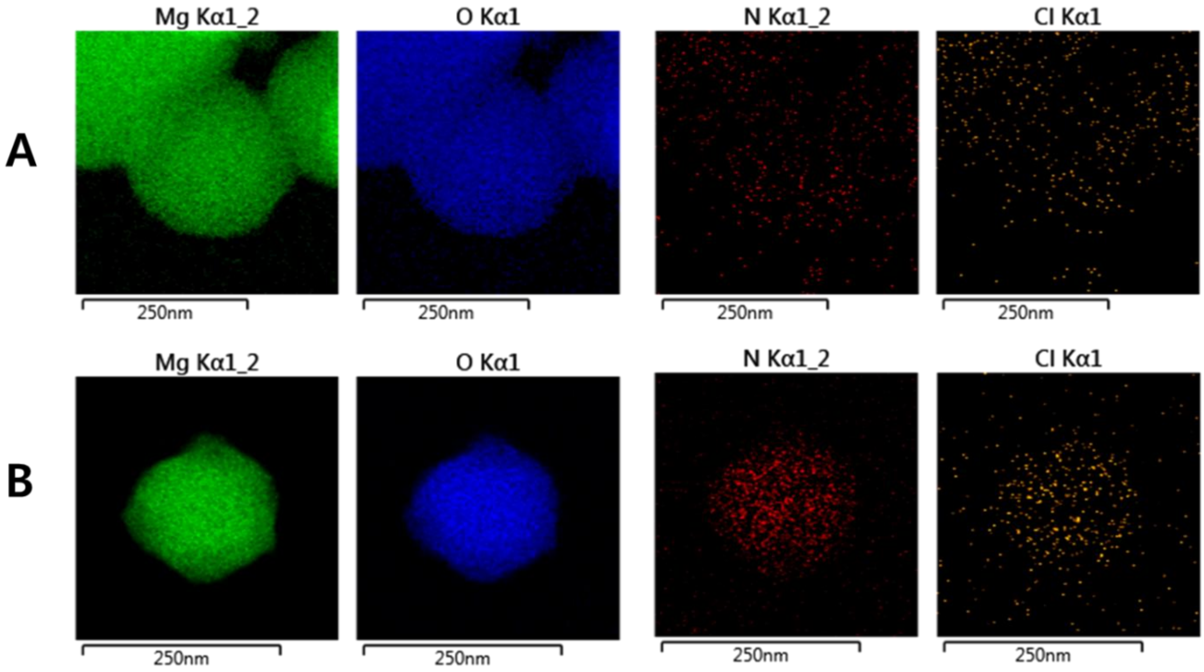

Figure S10. EDX for A) NIC-MgO and B) NIC-MgO-HPMC showing major elements, such as Mg, O, N and Cl. HPMC, hydroxy propyl methyl cellulose; MgO, magnesium oxide; NIC, niclosamide.

A

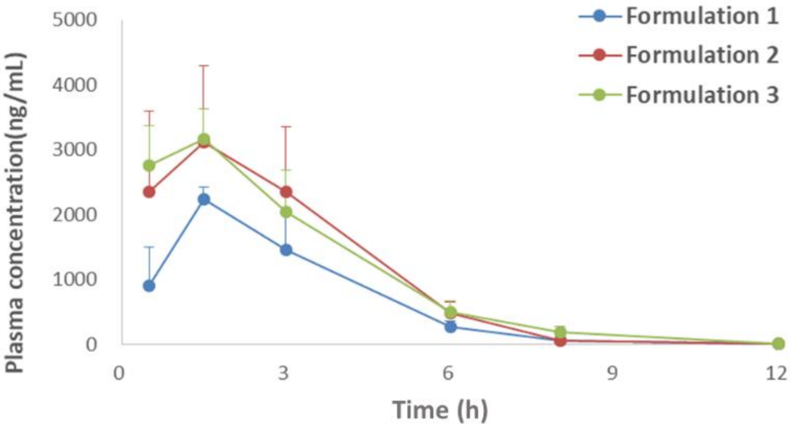

B

	Weight ratio			NIC (mg/kg)
	NIC	MgO	HPMC	
Formulation 1	4	2.8	1	300
Formulation 2	4	2.8	0.53	300
Formulation 3	4	2.8	0.27	300

Figure S11. (A) PK profiles of various NIC-MgO-HPMC formulation and (B) the formulation information. The PK studies were conducted using Balb/c nude mice through oral administration (n=3)

Table S1. Broad spectrum antiviral activity of niclosamide against various viral families

Family	Virus species	References
Coronaviridae	MERS-CoV,<br>SARS-CoV,<br>SARS-CoV-2,<br>Alpha (B. 1.1. 7),<br>Beta (B. 1.351) and<br>Delta variant (B. 1.617. 2)	Gassen et. al., Nat. Commun. (2019) 10: 5770;<br>Wu et. al., Antimicrob. Agents Chemother. (2004) 48: 2693;<br>Jeon et. al, Antimicrob Agents Chemother. (2020) 64(7):e00819;<br>Weis et.al., PLoS one. (2021) 16: e0260958.
Adenoviridae	Human adenovirus	Marrugal-Lorenzo et. al., (2019) Sci Rep 9: 17
Orthomyxoviridae	Influenza virus (IV)	Jurgeit et. al., PLoS Pathog. (2012) 8(10):e1002976;<br>Mazzon et. al., Viruses (2019) 11:176
Picornaviridae	Human rhinovirus (HRV),<br>Coxsackieviruses (CV)	Jurgeit et. al., PLoS Pathog. (2012) 8(10):e1002976
Herpesviridae	Herpes virus (HSV),<br>Epstein-Barr virus (EBV),<br>Kaposi's sarcoma-associated herpesvirus (KSHV)	Jurgeit et. al., PLoS Pathog. (2012) 8(10):e1002976;<br>Anderson et. al., (2019) Viruses 11:964;<br>Huang et. al., (2017) Antiviral Res. 38: 68−78
Pneumoviridae	Respiratory syncytial virus (RSV)	Niyomdecha N et. al., Virus Res. (2021) 295:198277
Flaviviridae	Zika virus (ZIKV),<br>Dengue virus (DENV),<br>West nile virus (WNV),<br>Yellow fever virus (YFV),<br>Japanese encephalitis virus (JEV),<br>Hepatitis C virus (HCV)	Simeonov et. al., (2016) Nat. Med. 22: 1101–7;<br>Li et. al., Cell Res. (2017) 27: 1046–64;<br>Edwards et. al., J. Med. Chem. (2011) 54: 8670−80
Togaviridae	Chikungunya virus (CHIKV),<br>Sindbis virus (SINV),<br>Semliki forest virus (SFV),<br>Ross river virus (RRV)	Wang et. al., Antiviral Res. (2016) 135:81-90;<br>Mazzon et. al., Viruses (2019) 11(10):176

Table S2. Breakdowns of protocol deviations leading to exclusion from the PPS

	**The placebo group n (%)**	**The low dose group n (%)**	**The high dose group n (%)**	**Total n (%)**
Randomized	100 (100.0)	100 (100.0)	100 (100.0)	100 (100.0)
Completed the planned study follow-up period	98 (98.0)	98(98.0)	95(95.0)	291 (97.0)
Early cessation of participations	2 (2.0)	2 (2.0)	5 (5.0)	9 (3.0)
Withdrawal of consent	2 (2.0)	1 (1.0)	4(4.0)	7 (2.3)
Assessment by the principal investigator	0 (0.0)	1 (1.0)	1 (1.0)	2 (0.7)
Early cessation of participations before treatment	2 (2.0)	1 (1.0)	4 (4.0)	7 (2.3)
Treatment within symptom onset > 3 days	5 (5.0)	9 (9.0)	7 (7.0)	21 (7.0)
Treatment within symptom onset ≤ Day3	93 (93.0)	90 (90.0)	89 (89.0)	272 (93.7)
PPS	77 (77.0)	70 (70.0)	80 (80.0)	227 (75.7)
PPS exclusion*	23 (23.0)	30 (30.0)	20 (20.0)	73 (24.3)
Major protocol deviations	14 (14.0)	19 (19.0)	10 (10.0)	43 (14.3)
Concomitant drugs affecting COVID-19 symptoms	9 (9.0)	14 (14.0)	5 (5.0)	28 (9.3)
Early cessation of participations	2 (2.0)	2 (2.0)	5 (5.0)	9 (3.0)
PCR missing or <0 in baseline	1 (1.0)	2 (2.0)	2 (2.0)	5 (1.7)
Major protocol deviations*	14 (14.0)	19 (19.0)	10 (10.0)	43 (14.3)
Viral load test omission	3 (3.0)	1 (1.0)	0 (0.0)	4 (1.3)
Randomized after screening test omission	1 (1.0)	0 (0.0)	1 (1.0)	2 (0.7)
Randomization error – PK group allocation	0 (0.0)	0 (0.0)	1 (1.0)	1 (0.3)
Randomization error – Severity allocation	9 (9.0)	9 (9.0)	3 (3.0)	21 (7.0)
Prohibited concomitant medication	0 (0.0)	1 (1.0)	3 (3.0)	4 (1.3)
Re-consent with updated consent form not obtained	3 (3.0)	7 (7.0)	2 (2.0)	12 (4.0)
IP treatment error	1 (1.0)	1 (1.0)	0 (0.0)	2 (0.7)
Consent form error	0 (0.0)	1 (1.0)	0 (0.0)	1 (0.3)

*There is overlap in the number of participants across the sub-categories

Table S3. Summary of adverse events (Safety analysis set)

	The placebo (n=98)	CP-COV03 The low dose group dose (n=99)	CP-COV03 450mg dose (n=96)
Patients with any TEAE, n (%)	26 (26.5)	21 (21.2)	33 (34.4)
Patients with any serious TEAE or death, n	0	0	0
Patients with TEAEs leading to treatment discontinuation, n	0	0	0
Patients with sequelae of TEAE, n	0	0	0
Cases of any TEAE, n	37	32	47
TEAEs occurring in ≥2% in either group			
Cardiomegaly, n	5	2	7
Abdominal pain upper, n	0	0	2
Dyspepsia, n	0	2	0
Pneumonia, n	8	9	7
Blood glucose increased, n	5	1	3
White blood cell count decreased, n	2	1	1
Eosinophil count increased, n	0	0	2
Lipase increased, n	1	2	2
Gamma-glutamyl transferase increased, n	2	0	1
Ageusia, n	0	1	2
Dysmenorrhea, n	0	0	2
Urticaria, n	1	1	3
Cases of any treatment-related AE, n	19	12	22
Treatment-related AEs occurring in ≥2% in either group			
Blood glucose increased, n	5	1	3
White blood cell count decreased, n	2	1	1
Eosinophil count increased, n	0	0	2
Lipase increased, n	1	2	2
Urticaria, n	1	1	3

Table S4. The mean number of days required for each targeted COVID-19 symptom to improve and be maintained for more

than 48 hours as analyzed in the (a) ITT, (b) PPS, and (c) mITT groups.

(a)

Symptoms	The placebo group vs The low dose group		The placebo vs The high dose group	
	LS Mean [95% CI]		LS Mean [95% CI]	
	The placebo	The low dose	The placebo	The high dose
**Fever**	5.0555 [4.0721-6.0390]	4.9028 [3.9963-5.8093]	5.0688 [3.9424-6.1951]	5.2548 [4.1284-6.3811]
**Cough**	6.8921 [6.0420-7.7421]	7.3154 [6.4653-8.1654]	6.9225 [6.0637-7.7814]	7.6030 [6.7487-8.4573]
**Sore throat**	5.3359 [4.6876-5.9842]	4.5136 [3.8653-5.1619]	5.3171 [4.7124-5.9218]	4.3711 [3.7664-4.9758]
**Headache**	5.5485 [4.8478-6.2493]	4.8890 [4.2036-5.5745]	5.5161 [4.7887-6.2435]	5.0121 [4.2806-5.7437]
**Muscle aches**	3.9062 [3.4625-4.3498]	3.6714 [3.2303-4.1126]	3.9124 [3.4272-4.3976]	3.9122 [3.4125-4.4119]
**Chills or shivering**	3.2944 [2.9016-3.6872]	3.4925 [3.1048-3.8801]	3.2876 [2.8544-3.7208]	3.5062 [3.0544-3.9580]
**Stuffy or runny nose**	7.6231 [6.7744-8.4717]	7.6141 [6.7919-8.4363]	7.6503 [6.8112-8.4894]	7.7491 [6.8861-8.6121]
**Tiredness or low energy**	5.7485 [4.9780-6.5190]	5.0259 [4.2680-5.7837]	5.7468 [4.9465-6.5471]	6.1352 [5.3349-6.9355]
**Difficulty breathing or shortness of breath**	4.5112 [3.4181-5.6043]	5.3964 [4.2890-6.5039]	4.4733 [3.3394-5.6072]	5.4370 [4.3030-6.5709]
**Nausea**	4.8050 [3.7545-5.8555]	5.2442 [4.4015-6.0870]	4.7161 [3.8264-5.6059]	4.2752 [3.5741-4.9763]
**Vomiting**	4.9981 [3.4724-6.5238]	4.6576 [3.3956-5.9195]	4.8465 [2.6869-7.0062]	4.3991 [2.4239-6.3743]
**Diarrhea**	6.5094 [5.7014-7.3174]	6.7935 [6.1213-7.4656]	6.4949 [5.7007-7.2890]	7.2193 [6.5842-7.8545]

(b)

Symptoms	The placebo group vs The low dose group		The placebo vs High dose group	
	LS Mean [95% CI]		LS Mean [95% CI]	
	The placebo	The low dose	The placebo	The high dose
**Fever**	5.0193 [4.0239-6.0148]	4.0877 [3.0497-5.1257]	4.9977 [3.6604-6.3350]	5.0599 [3.7486-6.3712]
**Cough**	6.9660 [6.0129-7.9192]	6.7315 [5.7298-7.7333]	6.9995 [6.0132-7.9858]	7.3929 [6.4383-8.3474]
**Sore throat**	5.2315 [4.5326-5.9304]	3.9941 [3.2645-4.7237]	5.2114 [4.5401-5.8828]	4.1437 [3.4900-4.7974]
**Headache**	5.4773 [4.7158-6.2387]	4.2476 [3.4573-5.0378]	5.4934 [4.7059-6.2809]	4.5338 [3.7626-5.3049]
**Muscle aches**	3.9680 [3.4910-4.4451]	3.3206 [2.8052-3.8360]	3.9665 [3.4025-4.5305]	3.8729 [3.3168-4.4290]
**Chills or shivering**	3.2403 [2.8996-3.5810]	3.1174 [2.7552-3.4797]	3.2301 [2.7644-3.6958]	3.3724 [2.9142-3.8306]
**Stuffy or runny nose**	7.4439 [6.5178-8.3700]	6.5159 [5.5633-7.4685]	7.4576 [6.5322-8.3830]	7.5766 [6.6512-8.5020]
**Tiredness or low energy**	5.6167 [4.8147-6.4186]	4.0979 [3.2421-4.9537]	5.6568 [4.7768-6.5368]	5.9918 [5.1118-6.8718]
**Difficulty breathing or shortness of breath**	4.4220 [3.1491-5.6949]	5.4820 [4.1211-6.8429]	4.3433 [3.0380-5.6486]	5.4067 [4.1014-6.7120]
**Nausea**	4.3755 [3.3439-5.4072]	4.2529 [3.3091-5.1967]	4.3745 [3.4220-5.3269]	4.0734 [3.3466-4.8003]
**Vomiting**	5.2535 [3.8310-6.6759]	3.3465 [1.9241-4.7690]	5.1902 [2.7372-7.6433]	4.4634 [2.1331-6.7937]
**Diarrhea**	6.4991 [5.6138-7.3843]	6.1396 [5.3691-6.9101]	6.4719 [5.5609-7.3830]	7.0022 [6.2979-7.7065]

(c)

Symptoms	The placebo group vs The low dose group		The placebo vs The high dose group	
	LS Mean [95% CI]		LS Mean [95% CI]	
	The placebo	The low dose	The placebo	The high dose
**Fever**	5.2379 [4.2551- 6.2207]	3.9824	5.1584	5.0603
		[3.0428- 4.9221]	[4.0251- 6.2917]	[3.9270- 6.1936]
**Cough**	6.9391	6.305	6.959	7.3598
	[6.0581- 7.8201]	[5.4240- 7.1860]	[6.0504- 7.8676]	[6.4618- 8.2578]
**Sore throat**	5.3099	3.8306	5.2914	4.1919
	[4.6799- 5.9400]	[3.2044- 4.4569]	[4.6743- 5.9085]	[3.5822- 4.8017]
**Headache**	5.2847	4.3598	5.247	4.7859
	[4.5592- 6.0102]	[3.6518- 5.0678]	[4.4922- 6.0038]	[4.0349- 5.5370]
**Muscle aches**	3.8021	3.2479	3.7892	4.0049
	[3.3861- 4.2182]	[2.8318- 3.6639]	[3.2870- 4.2914]	[3.4861- 4.5236]
**Chills or shivering**	3.3689	3.0873	3.3573	3.3838
	[2.9798- 3.7580]	[2.7120- 3.4625]	[2.9059- 3.8087]	[2.9218- 3.8457]
**Stuffy or runny nose**	7.4409	6.2653	7.489	7.5308
	[6.5599- 8.3219]	[5.4149- 7.1157]	[6.6083- 8.3697]	[6.6276- 8.4340]
**Tiredness or low energy**	5.5291	4.3873	5.4992	6.0254
	[4.7457- 6.3126]	[3.6133- 5.1612]	[4.6757- 6.3227]	[5.2019- 6.8490]
**Difficulty breathing or shortness of breath**	4.5992	4.8738	4.5557	5.1554
	[3.4749- 5.7234]	[3.7495- 5.9980]	[3.4345- 5.6769]	[4.0012- 6.3096]
**Nausea**	4.5482	4.5814	4.5487	4.1536
	[3.4822- 5.6142]	[3.7151- 5.4477]	[3.6265- 5.4708]	[3.4128- 4.8945]
**Vomiting**	4.1218	2.9063	3.9454	4.6705
	[2.6014- 5.6422]	[1.5796- 4.2330]	[1.7725- 6.1182]	[2.7045. 6.6365]
**Diarrhea**	6.3712	5.8189	6.3233	7.0268
	[5.4960- 7.2464]	[5.0851- 6.5527]	[5.4792- 7.1674]	[6.3483- 7.7053]

Table S5. Hospitals involved in this study

**Bestian Hospital**	**• Screened: 147**<br>**• Randomized: 135**<br>**• Completed: 133**
Gimpo Woori Hospital	• Screened: 106<br>• Randomized: 104<br>• Completed: 101
Korea University Ansan Hospital	• Screened: 3<br>• Randomized: 3<br>• Completed: 3
Chungnam National University Sejong Hospital	• Screened: 3<br>• Randomized: 3<br>• Completed: 3
Kyungpook National University Hospital	• Screened: 2<br>• Randomized: 2<br>• Completed: 2
Keimyung University Dongsan Hospital	• Screened: 6<br>• Randomized: 6<br>• Completed: 6
Hyundae General Hospital	• Screened: 34<br>• Randomized: 31<br>• Completed: 29
Kyungpook National University Chilgok Hospital	• Screened: 14<br>• Randomized: 14<br>• Completed: 12
Chosun University Hospital	• Screened: 2<br>• Randomized: 2<br>• Completed: 2

Table S6. Zeta potential for magnesium oxide (MgO) sample in 70% ethanol (pH ~9.4)

**Zeta potential (mV)**
+32.73
+32.90
+34.84
+36.62
+38.12
Average value = + 35.04 ± 2.34

Table S7. Chemical bond in FT-IR spectrum of intact niclosamide and its corresponding wavenumber according to the literature survey

Chemical bond	Wavenumber (cm^{-1})	Reference
-OH	3580	[3-5]
-NH	3484	
=C-H (aromatic)	3092	
C=O	1681	
C=C	1632	
-C-C- (aromatic)	1607	
-NH, C-N	1563	[3-7]
-N-O	1509	[3-5]
-N-O, -OH	1339	[3-7]

Table S8. Atomic % of elements in MgO, NIC-MgO, and NIC-MgO-HPMC samples

Samples	Atom	Atomic %
MgO	C *1s*	23.97
	N *1s*	3.12
	O *1s*	52.00
	Mg *1s*	20.91
NIC-MgO	C *1s*	76.44
	N *1s*	6.50
	O 1s	14.74
	Mg *1s*	2.32
NIC-MgO-HPMC	C *1s*	80.22
	N *1s*	5.80
	O *1s*	12.46
	Mg *1s*	1.52

Supplementary Note-2 (Secondary endpoints)

1) The time (days) taken to sustain symptom improvement for more than 48 hours by Day 14
Descriptive statistics for the time taken to sustain symptom improvement for over 48 hours by Day 14 for COVID-19 symptoms are presented for the placebo, CP-COV03 900 mg/day (the low dose), and CP-COV03 1,350 mg/day (the high dose) groups in Tables 1–3.
Participants who did not improve or sustain symptom improvement for more than 48 hours by the evening of Day 14 were censored to have the maximum improvement time as 13.00 days (maximum value 13 days = 14.5 days - (Day 1 of initial dose + 0.5 days)).
ANCOVA analysis was conducted for each symptom, considering age and severity as covariates, with results presented in Tables 4-6.

Table 1. Secondary endpoint 1) descriptive statistics (ITT)

Symptom		The placebo group N=98	The low dose group N=99	The high dose group N=96
Fever	N	34	40	34
	Mean	5.09	4.88	5.24
	SD	2.97	2.88	3.53
	Median	3.50	3.50	3.50
	Min	2.50	2.50	2.50
	Max	13.00	13.00	13.00
Cough	N	94	94	95
	Mean	6.91	7.29	7.61
	SD	4.16	4.27	4.23
	Median	5.50	6.00	6.50
	Min	2.50	1.50	2.50
	Max	13.00	13.00	13.00
Sore throat	N	93	93	93
	Mean	5.32	4.53	4.37
	SD	3.32	2.98	2.49
	Median	4.00	3.50	3.50
	Min	2.50	2.50	2.50
	Max	13.00	13.00	13.00
Headache	N	89	93	88
	Mean	5.54	4.89	4.98
	SD	3.76	3.10	3.21
	Median	3.50	3.50	3.50
	Min	2.50	2.50	2.50
	Max	13.00	13.00	13.00
Muscle ache	N	88	89	83
	Mean	3.89	3.69	3.94
	SD	2.22	2.01	2.39

Symptom		The placebo group N=98	The low dose group N=99	The high dose group N=96
	Median	3.00	3.00	3.00
	Min	2.50	1.50	2.50
	Max	13.00	13.00	13.00
Chill	N	75	77	69
	Mean	3.28	3.51	3.51
	SD	1.61	1.94	2.13
	Median	2.50	2.50	2.50
	Min	2.50	2.50	2.50
	Max	12.00	13.00	13.00
Stuffy or runny nose	N	92	98	87
	Mean	7.61	7.62	7.79
	SD	4.23	4.01	3.87
	Median	6.75	6.25	7.00
	Min	2.50	2.50	2.50
	Max	13.00	13.00	13.00
Fatigue	N	89	92	89
	Mean	5.73	5.04	6.15
	SD	3.80	3.72	3.93
	Median	3.50	3.25	4.00
	Min	2.50	2.50	2.50
	Max	13.00	13.00	13.00
Difficulty of breathing	N	39	38	39
	Mean	4.54	5.37	5.37
	SD	2.86	3.97	3.99
	Median	3.50	3.00	3.50
	Min	2.50	2.50	2.50
	Max	13.00	13.00	13.00
Nausea	N	38	59	61
	Mean	4.76	5.27	4.25
	SD	3.15	3.43	2.46
	Median	3.00	4.00	3.50
	Min	2.50	2.50	2.50
	Max	13.00	13.00	13.00
Vomiting	N	11	16	13
	Mean	4.59	4.94	4.62
	SD	3.69	2.78	2.91

Symptom		The placebo group N=98	The low dose group N=99	The high dose group N=96
	Median	3.00	4.00	3.50
	Min	2.50	2.50	2.50
	Max	12.00	13.00	13.00
Diarrhea	N	52	75	81
	Mean	6.42	6.85	7.27
	SD	3.22	2.85	2.67
	Median	6.00	6.50	7.50
	Min	2.50	2.50	2.50
	Max	13.00	13.00	13.00

Table 2. Secondary endpoint 1) descriptive statistics (mITT1)

Symptom		The placebo group N=88	The low dose group N=89	The high dose group N=87
Fever	N	32	35	32
	Mean	5.23	3.99	4.98
	SD	3.00	2.65	3.39
	Median	3.50	3.50	3.50
	Min	2.50	1.00	2.00
	Max	13.00	13.00	13.00
Cough	N	84	84	86
	Mean	6.95	6.29	7.37
	SD	4.16	4.19	4.25
	Median	5.50	4.50	6.00
	Min	2.50	0.50	0.50
	Max	13.00	13.00	13.00
Sore throat	N	83	84	85
	Mean	5.28	3.86	4.20
	SD	3.27	2.50	2.35
	Median	4.00	3.00	3.50
	Min	2.50	0.50	0.50
	Max	13.00	13.00	13.00
Headache	N	80	84	81
	Mean	5.26	4.38	4.77
	SD	3.67	2.97	3.15
	Median	3.50	3.50	3.50
	Min	2.50	0.50	0.50
	Max	13.00	13.00	13.00

Symptom		The placebo group N=88	The low dose group N=89	The high dose group N=87
Muscle ache	N	80	80	75
	Mean	3.78	3.28	4.02
	SD	2.03	1.76	2.50
	Median	3.00	2.75	3.00
	Min	2.50	0.50	2.00
	Max	11.00	13.00	13.00
Chill	N	67	72	64
	Mean	3.35	3.10	3.39
	SD	1.68	1.59	2.00
	Median	3.00	2.50	2.50
	Min	2.50	0.50	2.00
	Max	12.00	12.50	13.00
Stuffy or runny nose	N	82	88	78
	Mean	7.41	6.29	7.61
	SD	4.23	3.83	3.86
	Median	6.50	5.00	7.00
	Min	2.50	0.50	2.00
	Max	13.00	13.00	13.00
Fatigue	N	81	83	81
	Mean	5.49	4.43	6.04
	SD	3.76	3.50	3.89
	Median	3.50	3.00	4.00
	Min	2.50	0.50	0.50
	Max	13.00	13.00	13.00
Difficulty of breathing	N	37	37	35
	Mean	4.65	4.82	5.06
	SD	2.90	3.93	3.78
	Median	3.50	3.00	3.00
	Min	2.50	0.50	2.50
	Max	13.00	13.00	13.00
Nausea	N	35	53	54
	Mean	4.54	4.58	4.16
	SD	2.90	3.32	2.56
	Median	3.00	3.50	3.50
	Min	2.50	0.50	0.50
	Max	13.00	13.00	13.00

Symptom		The placebo group N=88	The low dose group N=89	The high dose group N=87
Vomiting	N	10	13	12
	Mean	3.85	3.12	4.75
	SD	2.90	1.76	3.00
	Median	3.00	3.00	3.75
	Min	2.50	0.50	2.50
	Max	12.00	6.00	13.00
Diarrhea	N	48	68	74
	Mean	6.23	5.92	7.09
	SD	3.26	3.01	2.77
	Median	5.50	5.75	7.00
	Min	2.50	0.50	2.00
	Max	13.00	13.00	13.00

Table 3. Secondary endpoint 1) descriptive statistics (PPS)

Symptom		The placebo group N=77	The low dose group N=70	The high dose group N=80
Fever	N	25	23	26
	Mean	5.00	4.11	5.06
	SD	2.88	1.80	3.59
	Median	3.50	3.50	3.50
	Min	2.50	2.50	2.50
	Max	13.00	9.00	13.00
Cough	N	74	67	79
	Mean	6.99	6.71	7.41
	SD	4.25	4.09	4.29
	Median	5.25	5.00	6.00
	Min	2.50	2.50	2.50
	Max	13.00	13.00	13.00
Sore throat	N	73	67	77
	Mean	5.21	4.02	4.15
	SD	3.39	2.62	2.33
	Median	4.00	3.00	3.50
	Min	2.50	2.50	2.50
	Max	13.00	13.00	13.00
Headache	N	70	65	73
	Mean	5.49	4.24	4.54
	SD	3.74	2.59	2.88

Symptom		The placebo group N=77	The low dose group N=70	The high dose group N=80
	Median	3.50	3.50	3.50
	Min	2.50	2.50	2.50
	Max	13.00	13.00	13.00
Muscle ache	N	70	60	72
	Mean	3.96	3.33	3.88
	SD	2.36	1.53	2.39
	Median	3.00	3.00	3.00
	Min	2.50	2.50	2.50
	Max	13.00	13.00	13.00
Chill	N	61	54	63
	Mean	3.23	3.13	3.37
	SD	1.57	1.04	2.03
	Median	2.50	2.75	2.50
	Min	2.50	2.50	2.50
	Max	12.00	7.00	13.00
Stuffy or runny nose	N	73	69	73
	Mean	7.41	6.55	7.62
	SD	4.35	3.65	3.79
	Median	6.00	5.00	7.00
	Min	2.50	2.50	2.50
	Max	13.00	13.00	13.00
Fatigue	N	74	65	74
	Mean	5.59	4.13	6.06
	SD	3.89	3.05	3.96
	Median	3.50	3.00	4.00
	Min	2.50	2.50	2.50
	Max	13.00	13.00	13.00
Difficulty of breathing	N	32	28	32
	Mean	4.39	5.52	5.36
	SD	3.00	4.18	4.12
	Median	3.00	3.25	3.00
	Min	2.50	2.50	2.50
	Max	13.00	13.00	13.00
Nausea	N	31	37	53
	Mean	4.34	4.28	4.09
	SD	2.90	2.79	2.48

Symptom		The placebo group N=77	The low dose group N=70	The high dose group N=80
	Median	3.00	3.00	3.50
	Min	2.50	2.50	2.50
	Max	13.00	13.00	13.00
Vomiting	N	10	10	11
	Mean	4.80	3.80	4.82
	SD	3.82	1.57	3.13
	Median	3.00	3.25	3.50
	Min	2.50	2.50	2.50
	Max	12.00	7.00	13.00
Diarrhea	N	41	54	68
	Mean	6.38	6.23	7.06
	SD	3.31	2.61	2.67
	Median	5.50	6.25	7.00
	Min	2.50	2.50	2.50
	Max	13.00	13.00	13.00

Table 4. Secondary endpoint 1) ANCOVA results (ITT)

Symptom	The placebo group vs The low dose group			The placebo group vs The high dose group		
	LS Mean [95% CI]		p-value	LS Mean [95% CI]		p-value
	The placebo	The low dose		The placebo	The high dose	
Fever	34	40		34	34	
	5.0555 [4.0721, 6.0390]	4.9028 [3.9963, 5.8093]	0.8208	5.0688 [3.9424, 6.1951]	5.2548 [4.1284, 6.3811]	0.8164
Cough	94	94		94	95	
	6.8921 [6.0420, 7.7421]	7.3154 [6.4653, 8.1654]	0.4883	6.9225 [6.0637, 7.7814]	7.6030 [6.7487, 8.4573]	0.2699
Sore throat	93	93		93	93	
	5.3359 [4.6876, 5.9842]	4.5136 [3.8653, 5.1619]	0.0789	5.3171 [4.7124, 5.9218]	4.3711 [3.7664, 4.9758]	0.0305*
Headache	89	93		89	88	
	5.5485 [4.8478, 6.2493]	4.8890 [4.2036, 5.5745]	0.1863	5.5161 [4.7887, 6.2435]	5.0121 [4.2806, 5.7437]	0.3370
Muscle ache	88	89		88	83	
	3.9062 [3.4625, 4.3498]	3.6714 [3.2303, 4.1126]	0.4605	3.9124 [3.4272, 4.3976]	3.9122 [3.4125, 4.4119]	0.9995
Chill	75	77		75	69	
	3.2944 [2.9016, 3.6872]	3.4925 [3.1048, 3.8801]	0.4799	3.2876 [2.8544, 3.7208]	3.5062 [3.0544, 3.9580]	0.4931

Symptom	The placebo group vs The low dose group			The placebo group vs The high dose group		
	LS Mean [95% CI]		p-value	LS Mean [95% CI]		p-value
	The placebo	The low dose		The placebo	The high dose	
Stuffy or runny nose	92	98		92	87	
	7.6231 [6.7744, 8.4717]	7.6141 [6.7919, 8.4363]	0.9881	7.6503 [6.8112, 8.4894]	7.7491 [6.8861, 8.6121]	0.8719
Fatigue	89	92		89	89	
	5.7485 [4.9780, 6.5190]	5.0259 [4.2680, 5.7837]	0.1888	5.7468 [4.9465, 6.5471]	6.1352 [5.3349, 6.9355]	0.4994
Difficulty of breathing	39	38		39	39	
	4.5112 [3.4181, 5.6043]	5.3964 [4.2890, 6.5039]	0.2610	4.4733 [3.3394, 5.6072]	5.4370 [4.3030, 6.5709]	0.2413
Nausea	38	59		38	61	
	4.8050 [3.7545, 5.8555]	5.2442 [4.4015, 6.0870]	0.5191	4.7161 [3.8264, 5.6059]	4.2752 [3.5741, 4.9763]	0.4431
Vomiting	11	16		11	13	
	4.9981 [3.4724, 6.5238]	4.6576 [3.3956, 5.9195]	0.7269	4.8465 [2.6869, 7.0062]	4.3991 [2.4239, 6.3743]	0.7609
Diarrhea	52	75		52	81	
	6.5094 [5.7014, 7.3174]	6.7935 [6.1213, 7.4656]	0.5945	6.4949 [5.7007, 7.2890]	7.2193 [6.5842, 7.8545]	0.1627

* Statistically significant difference was observed at the 5% significance level

Table 5. Secondary endpoint 1) ANCOVA results (mITT1)

Symptom	The placebo group vs The low dose group			The placebo group vs The high dose group		
	LS Mean [95% CI]		p-value	LS Mean [95% CI]		p-value
	The placebo	The low dose		The placebo	The high dose	
Fever	32	35		32	32	
	5.2379 [4.2551, 6.2207]	3.9824 [3.0428, 4.9221]	0.0699	5.1584 [4.0251, 6.2917]	5.0603 [3.9270, 6.1936]	0.9033
Cough	84	84		84	86	
	6.9391 [6.0581, 7.8201]	6.3050 [5.4240, 7.1860]	0.3167	6.9590 [6.0504, 7.8676]	7.3598 [6.4618, 8.2578]	0.5369
Sore throat	83	84		83	85	
	5.3099 [4.6799, 5.9400]	3.8306 [3.2044, 4.4569]	0.0013*	5.2914 [4.6743, 5.9085]	4.1919 [3.5822, 4.8017]	0.0134*
Headache	80	84		80	81	
	5.2847 [4.5592, 6.0102]	4.3598 [3.6518, 5.0678]	0.0738	5.2470 [4.4922, 6.0038]	4.7859 [4.0349, 5.5370]	0.3934
Muscle ache	80	80		80	75	
	3.8021 [3.3861,	3.2479 [2.8318,	0.0652	3.7892 [3.2870,	4.0049 [3.4861,	0.5573

Symptom	The placebo group vs The low dose group			The placebo group vs The high dose group		
	LS Mean [95% CI]		p-value	LS Mean [95% CI]		p-value
	The placebo	The low dose		The placebo	The high dose	
	4.2182]	3.6639]		4.2914]	4.5236]	
Chill	67	72		67	64	
	3.3689 [2.9798, 3.7580]	3.0873 [2.7120, 3.4625]	0.3056	3.3573 [2.9059, 3.8087]	3.3838 [2.9218, 3.8457]	0.9358
Stuffy or runny nose	82	88		82	78	
	7.4409 [6.5599, 8.3219]	6.2653 [5.4149, 7.1157]	0.0600	7.4890 [6.6083, 8.3697]	7.5308 [6.6276, 8.4340]	0.9481
Fatigue	81	83		81	81	
	5.5291 [4.7457, 6.3126]	4.3873 [3.6133, 5.1612]	0.0424*	5.4992 [4.6757, 6.3227]	6.0254 [5.2019, 6.8490]	0.3741
Difficulty of breathing	37	37		37	35	
	4.5992 [3.4749, 5.7234]	4.8738 [3.7495, 5.9980]	0.7318	4.5557 [3.4345, 5.6769]	5.1554 [4.0012, 6.3096]	0.4689
Nausea	35	53		35	54	
	4.5482 [3.4822, 5.6142]	4.5814 [3.7151, 5.4477]	0.9617	4.5487 [3.6265, 5.4708]	4.1536 [3.4128, 4.8945]	0.5105
Vomiting	10	13		10	12	
	4.1218 [2.6014, 5.6422]	2.9063 [1.5796, 4.2330]	0.2310	3.9454 [1.7725, 6.1182]	4.6705 [2.7045. 6.6365]	0.6256
Diarrhea	48	68		48	74	
	6.3712 [5.4960, 7.2464]	5.8189 [5.0851, 6.5527]	0.3428	6.3233 [5.4792, 7.1674]	7.0268 [6.3483, 7.7053]	0.2028

* Statistically significant difference was observed at the 5% significance level

Table 6. Secondary endpoint 1) ANCOVA results (PPS)

Symptom	The placebo group vs The low dose group			The placebo group vs The high dose group		
	LS Mean [95% CI]		p-value	LS Mean [95% CI]		p-value
	The placebo	The low dose		The placebo	The high dose	
Fever	25	23		25	26	
	5.0193 [4.0239, 6.0148]	4.0877 [3.0497, 5.1257]	0.1993	4.9977 [3.6604, 6.3350]	5.0599 [3.7486, 6.3712]	0.9470
Cough	74	67		74	79	
	6.9660 [6.0129, 7.9192]	6.7315 [5.7298, 7.7333]	0.7381	6.9995 [6.0132, 7.9858]	7.3929 [6.4383, 8.3474]	0.5723
Sore throat	73	67		73	77	
	5.2315 [4.5326, 5.9304]	3.9941 [3.2645, 4.7237]	0.0168*	5.2114 [4.5401, 5.8828]	4.1437 [3.4900, 4.7974]	0.0259*

Symptom	The placebo group vs The low dose group			The placebo group vs The high dose group		
	LS Mean [95% CI]		p-value	LS Mean [95% CI]		p-value
	The placebo	The low dose		The placebo	The high dose	
Headache	70	65		70	73	
	5.4773 [4.7158, 6.2387]	4.2476 [3.4573, 5.0378]	0.0285*	5.4934 [4.7059, 6.2809]	4.5338 [3.7626, 5.3049]	0.0875
Muscle ache	70	60		70	72	
	3.9680 [3.4910, 4.4451]	3.3206 [2.8052, 3.8360]	0.0708	3.9665 [3.4025, 4.5305]	3.8729 [3.3168, 4.4290]	0.8158
Chill	61	54		61	63	
	3.2403 [2.8996, 3.5810]	3.1174 [2.7552, 3.4797]	0.6263	3.2301 [2.7644, 3.6958]	3.3724 [2.9142, 3.8306]	0.6672
Stuffy or runny nose	73	69		73	73	
	7.4439 [6.5178, 8.3700]	6.5159 [5.5633, 7.4685]	0.1699	7.4576 [6.5322, 8.3830]	7.5766 [6.6512, 8.5020]	0.8579
Fatigue	74	65		74	73	
	5.6167 [4.8147, 6.4186]	4.0979 [3.2421, 4.9537]	0.0116*	5.6568 [4.7768, 6.5368]	5.9918 [5.1118, 6.8718]	0.5960
Difficulty of breathing	32	28		32	32	
	4.4220 [3.1491, 5.6949]	5.4820 [4.1211, 6.8429]	0.2596	4.3433 [3.0380, 5.6486]	5.4067 [4.1014, 6.7120]	0.2596
Nausea	31	37		31	53	
	4.3755 [3.3439, 5.4072]	4.2529 [3.3091, 5.1967]	0.8619	4.3745 [3.4220, 5.3269]	4.0734 [3.3466, 4.8003]	0.6197
Vomiting	10	10		10	11	
	5.2535 [3.8310, 6.6759]	3.3465 [1.9241, 4.7690]	0.0665	5.1902 [2.7372, 7.6433]	4.4634 [2.1331, 6.7937]	0.6678
Diarrhea	41	54		41	68	
	6.4991 [5.6138, 7.3843]	6.1396 [5.3691, 6.9101]	0.5462	6.4719 [5.5609, 7.3830]	7.0022 [6.2979, 7.7065]	0.3672

* Statistically significant difference was observed at the 5% significance level

In the ITT analysis, ANCOVA results indicated a significant difference at the 5% significance level in the time of sustained symptom improvement for more than 48 hours in the sore throat symptom between the placebo and the high dose group (p=0.0305). The LSMEAN [95% confidence interval] was 5.32 [4.71, 5.92] for the placebo group and 4.37 [3.77, 4.98] for the high dose group, indicating that symptom improvement for sore throat occurred 0.95 days faster in the latter compared to the former.

In the mITT1 analysis, ANCOVA results showed significant differences at the 5% significance level in sore throat and fatigue symptoms between the placebo and the low dose group. The latter indicated faster symptom improvement by 1.48 days for sore throat and 1.14 days for fatigue compared to the former. The time of sustained symptom improvement for more than 48 hours [95% confidence interval] for significant differences were as follows:

Sore throat (p=0.0013): The placebo group 5.31 [4.68, 5.94], The low dose group 3.83 [3.20, 4.46]
Fatigue (p=0.0424): The placebo group 5.53 [4.75, 6.31], The low dose group 4.39 [3.61, 5.16]
A significant difference (p=0.0134) was observed in the time of sustained symptom improvement for more than 48 hours in sore throat between the placebo and the high dose group, with LSMEAN [95% confidence interval] of 5.29 [4.67, 5.91] for the placebo group and 4.19 [3.58, 4.80] for the high dose group, indicating that the latter showed a faster symptom improvement by 1.10 days than the former.
In the PPS analysis, ANCOVA results at the 5% significance level showed significant differences in sore throat, headache, and fatigue symptoms between the placebo group and the low dose group, indicating that the latter showed faster symptom improvement by 1.24 days for sore throat, 1.23 days for headache, and 1.52 days for fatigue than the former. The time of sustained symptom improvement for more than 48 hours [95% confidence interval] for significant differences were as follows:
Sore throat (p=0.0168): The placebo group 5.23 [4.53, 5.93], The low dose group 3.99 [3.26, 4.72]
Headache (p=0.0285): The placebo group 5.48 [4.72, 6.24], The low dose group 4.25 [3.46, 5.04]
Fatigue (p=0.0116): The placebo group 5.62 [4.81, 6.42], The low dose group 4.10 [3.24, 4.95]
Additionally, significant difference (p=0.0259) was observed in the time of sustained symptom improvement for more than 48 hours in sore throat between the placebo and the high dose group, with LSMEAN [95% confidence interval] of 5.21 [4.54, 5.88] for the placebo group and 4.14 [3.49, 4.80] for the high dose group, indicating faster symptom improvement by 1.07 days in the high dose group.

2) Time (days) taken improvement of each COVID-19 symptom by Day 14
Descriptive statistics for the time (days) taken improvement of each COVID-19 symptom by Day 14 for the placebo, the low dose, and the high dose groups were presented in Tables 7–9 by symptom. Participants not improved symptoms by the evening of Day 14.5 were censored to have a maximum improvement time as 13.00 days (maximum value 13 days = 14.5 days - (first day of administration + 0.5 day)). ANCOVA analysis was conducted for each symptom, considering age and Severity as covariates, and the results were presented in Tables 10–12.

Table 7. Secondary endpoint 2) descriptive statistics (ITT)

Symptom		The placebo group N=98	The low dose group N=99	The high dose group N=96
Fever	N	34	40	34
	Mean	3.18	2.93	3.40
	SD	3.22	3.04	3.91
	Median	1.50	1.50	1.50
	Min	0.50	0.50	0.50
	Max	13.00	13.00	13.00
Cough	N	94	94	95
	Mean	5.30	5.80	6.10
	SD	4.76	4.98	4.89
	Median	3.50	4.00	4.50
	Min	0.50	-0.50*	0.50
	Max	13.00	13.00	13.00
Sore throat	N	93	93	93
	Mean	3.45	2.67	2.43
	SD	3.64	3.38	2.71
	Median	2.00	1.50	1.50

Symptom		The placebo group N=98	The low dose group N=99	The high dose group N=96
	Min	0.50	0.50	0.50
	Max	13.00	13.00	13.00
Headache	N	89	93	88
	Mean	3.73	3.03	3.10
	SD	4.15	3.48	3.53
	Median	1.50	1.50	1.50
	Min	0.50	0.50	0.50
	Max	13.00	13.00	13.00
Muscle ache	N	88	89	83
	Mean	1.91	1.71	1.96
	SD	2.32	2.12	2.46
	Median	1.00	1.00	1.00
	Min	0.50	-0.50*	0.50
	Max	13.00	13.00	12.50
Chill	N	75	77	69
	Mean	1.28	1.53	1.57
	SD	1.61	2.08	2.37
	Median	0.50	0.50	0.50
	Min	0.50	0.50	0.50
	Max	10.00	13.00	13.00
Stuffy or runny nose	N	92	98	87
	Mean	6.01	6.00	6.17
	SD	4.78	4.56	4.42
	Median	4.75	4.25	5.00
	Min	0.50	0.50	0.50
	Max	13.00	13.00	13.00
Fatigue	N	89	92	89
	Mean	4.02	3.28	4.40
	SD	4.39	4.24	4.39
	Median	1.50	1.25	2.00
	Min	0.50	0.50	0.50
	Max	13.00	13.00	13.00
Difficulty of breathing	N	39	38	39
	Mean	2.59	3.64	3.63
	SD	3.03	4.53	4.52
	Median	1.50	1.00	1.50

Symptom		The placebo group N=98	The low dose group N=99	The high dose group N=96
	Min	0.50	0.50	0.50
	Max	13.00	13.00	13.00
Nausea	N	38	59	61
	Mean	2.82	3.41	2.28
	SD	3.30	3.76	2.59
	Median	1.00	2.00	1.50
	Min	0.50	0.50	0.50
	Max	12.50	13.00	13.00
Vomiting	N	11	16	13
	Mean	2.59	3.03	2.73
	SD	3.69	3.08	3.28
	Median	1.00	2.00	1.50
	Min	0.50	0.50	0.50
	Max	10.00	12.50	12.50
Diarrhea	N	52	75	81
	Mean	4.46	5.01	5.41
	SD	3.31	3.20	3.01
	Median	4.00	4.50	5.50
	Min	0.50	0.50	0.50
	Max	13.00	13.00	13.00

* In the low dose group, one participant (S02008) experienced an improvement in cough and muscle ache in the morning of Day 2. However, as the first IP administration did not occur until the noon of Day 2 (resulting in a missed Day 1 night and Day 2 morning dose), the formula for calculating the time (days) required for symptom improvement resulted in a negative number. The calculation was based on the formula: (Day of symptom improvement observation - (First day of administration + 0.5 days)) = 2 (morning of Day 2) - (2 (Day 2 of first administration) + 0.5) = -0.5.

Table 8. Secondary endpoint 2) descriptive statistics (mITT1)

Symptom		The placebo group N=88	The low dose group N=89	The high dose group N=87
Fever	N	32	35	32
	Mean	3.33	2.36	3.30
	SD	3.26	2.48	3.68
	Median	1.50	1.50	1.50
	Min	0.50	0.50	0.50
	Max	13.00	11.00	13.00
Cough	N	84	84	86
	Mean	5.39	4.88	5.91

Symptom		The placebo group N=88	The low dose group N=89	The high dose group N=87
	SD	4.76	4.68	4.80
	Median	3.50	2.50	4.25
	Min	0.50	0.50	0.50
	Max	13.00	13.00	13.00
Sore throat	N	83	84	85
	Mean	3.44	2.17	2.34
	SD	3.56	2.78	2.50
	Median	2.00	1.00	1.50
	Min	0.50	0.50	0.50
	Max	13.00	13.00	13.00
Headache	N	80	84	81
	Mean	3.46	2.70	2.93
	SD	3.99	3.16	3.41
	Median	1.50	1.50	1.50
	Min	0.50	0.50	0.50
	Max	13.00	13.00	13.00
Muscle ache	N	80	80	75
	Mean	1.80	1.50	2.08
	SD	2.03	1.85	2.56
	Median	1.00	1.00	1.00
	Min	0.50	0.50	0.50
	Max	9.00	13.00	12.50
Chill	N	67	72	64
	Mean	1.36	1.31	1.52
	SD	1.68	1.55	2.32
	Median	1.00	0.50	0.50
	Min	0.50	0.50	0.50
	Max	10.00	10.50	13.00
Stuffy or runny nose	N	82	88	78
	Mean	5.86	4.70	6.03
	SD	4.70	3.97	4.33
	Median	4.50	3.25	5.00
	Min	0.50	0.50	0.50
	Max	13.00	13.00	13.00
Fatigue	N	81	83	81
	Mean	3.83	2.81	4.34

Symptom		The placebo group N=88	The low dose group N=89	The high dose group N=87
	SD	4.30	3.89	4.26
	Median	1.50	1.00	2.50
	Min	0.50	0.50	0.50
	Max	13.00	13.00	13.00
Difficulty of breathing	N	37	37	35
	Mean	2.70	3.24	3.29
	SD	3.07	4.30	4.29
	Median	1.50	1.00	1.00
	Min	0.50	0.50	0.50
	Max	13.00	13.00	13.00
Nausea	N	35	53	54
	Mean	2.61	3.01	2.31
	SD	2.93	3.46	2.64
	Median	1.00	1.50	1.50
	Min	0.50	0.50	0.50
	Max	11.50	13.00	13.00
Vomiting	N	10	13	12
	Mean	1.85	1.88	2.88
	SD	2.90	1.33	3.38
	Median	1.00	1.50	1.75
	Min	0.50	0.50	0.50
	Max	10.00	4.00	12.50
Diarrhea	N	48	68	74
	Mean	4.35	4.31	5.29
	SD	3.30	3.04	3.05
	Median	3.50	4.00	5.00
	Min	0.50	0.50	0.50
	Max	13.00	13.00	13.00

Table 9. Secondary endpoint 2) descriptive statistics (PPS)

Symptom		The placebo group N=77	The low dose group N=70	The high dose group N=80
Fever	N	25	23	26
	Mean	3.08	2.11	3.21
	SD	3.13	1.80	3.96
	Median	1.50	1.50	1.50
	Min	0.50	0.50	0.50

Symptom		The placebo group N=77	The low dose group N=70	The high dose group N=80
	Max	13.00	7.00	13.00
Cough	N	74	67	79
	Mean	5.42	5.09	5.89
	SD	4.89	4.71	4.96
	Median	3.25	3.00	4.00
	Min	0.50	0.50	0.50
	Max	13.00	13.00	13.00
Sore throat	N	73	67	77
	Mean	3.34	2.14	2.19
	SD	3.72	3.05	2.52
	Median	2.00	1.00	1.50
	Min	0.50	0.50	0.50
	Max	13.00	13.00	13.00
Headache	N	70	65	73
	Mean	3.66	2.33	2.65
	SD	4.11	2.92	3.23
	Median	1.50	1.50	1.50
	Min	0.50	0.50	0.50
	Max	13.00	13.00	13.00
Muscle ache	N	70	60	72
	Mean	1.99	1.37	1.90
	SD	2.48	1.75	2.48
	Median	1.00	1.00	1.00
	Min	0.50	0.50	0.50
	Max	13.00	13.00	12.50
Chill	N	61	54	63
	Mean	1.23	1.13	1.43
	SD	1.57	1.04	2.31
	Median	0.50	0.75	0.50
	Min	0.50	0.50	0.50
	Max	10.00	5.00	13.00
Stuffy or runny nose	N	73	69	73
	Mean	5.80	4.72	5.96
	SD	4.89	3.98	4.31
	Median	4.00	3.00	5.00
	Min	0.50	0.50	0.50

Symptom		The placebo group N=77	The low dose group N=70	The high dose group N=80
	Max	13.00	13.00	13.00
Fatigue	N	74	65	74
	Mean	3.89	2.28	4.30
	SD	4.48	3.51	4.42
	Median	1.50	1.00	2.00
	Min	0.50	0.50	0.50
	Max	13.00	13.00	13.00
Difficulty of breathing	N	32	28	32
	Mean	2.45	3.82	3.61
	SD	3.20	4.76	4.62
	Median	1.00	1.25	1.00
	Min	0.50	0.50	0.50
	Max	13.00	13.00	13.00
Nausea	N	31	37	53
	Mean	2.35	2.34	2.13
	SD	2.95	2.97	2.63
	Median	1.00	1.00	1.50
	Min	0.50	0.50	0.50
	Max	11.50	13.00	13.00
Vomiting	N	10	10	11
	Mean	2.80	1.80	2.95
	SD	3.82	1.57	3.53
	Median	1.00	1.25	1.50
	Min	0.50	0.50	0.50
	Max	10.00	5.00	12.50
Diarrhea	N	41	54	68
	Mean	4.43	4.33	5.20
	SD	3.43	2.89	3.01
	Median	3.50	4.25	5.00
	Min	0.50	0.50	0.50
	Max	13.00	13.00	13.00

Table 10. Secondary endpoint 2) ANCOVA results (ITT)

Symptom	The placebo group vs The low dose group			The placebo group vs The high dose group		
	LS Mean [95% CI]		p-value	LS Mean [95% CI]		p-value
	The placebo	The Low dose		The placebo	The high dose	
Fever	34	40		34	34	
	3.1429 [2.0867, 4.1990]	2.9536 [1.9801, 3.9271]	0.7937	3.1565 [1.9181, 4.3949]	3.4170 [2.1786, 4.6554]	0.7675
Cough	94	94		94	95	
	5.2764 [4.2937, 6.2592]	5.8299 [4.8472, 6.8127]	0.4332	5.3134 [4.3253, 6.3015]	6.0899 [5.1070, 7.0728]	0.2738
Sore throat	93	93		93	93	
	3.4675 [2.7457, 4.1892]	2.6454 [1.9237, 3.3672]	0.1143	3.4477 [2.7863, 4.1090]	2.4287 [1.7673, 3.0900]	0.0331*
Headache	89	93		89	88	
	3.7346 [2.9571, 4.5121]	3.0228 [2.2623, 3.7834]	0.1985	3.6986 [2.8966, 4.5006]	3.1344 [2.3278, 3.9409]	0.3296
Muscle ache	88	89		88	83	
	1.9298 [1.4636, 2.3961]	1.6930 [1.2294, 2.1566]	0.4785	1.9371 [1.4336, 2.4406]	1.9282 [1.4096, 2.4467]	0.9807
Chill	75	77		75	69	
	1.2952 [0.8868, 1.7036]	1.5177 [1.1147, 1.9207]	0.4453	1.2893 [0.8258, 1.7528]	1.5551[1.0717, 2.0385]	0.4360
Stuffy or runny nose	92	98		92	87	
	6.0169 [5.0557, 6.9781]	5.9943 [5.0630, 6.9257]	0.9735	6.0461 [5.0935, 6.9988]	6.1294 [5.1495, 7.1092]	0.9048
Fatigue	89	92		89	89	
	4.0423 [3.1582, 4.9265]	3.2579 [2.3884, 4.1275]	0.2137	4.0398 [3.1306, 4.9489]	4.3816 [3.4725, 5.2907]	0.6007
Difficulty of breathing	39	38		39	39	
	2.5637 [1.3456, 3.7818]	3.6715 [2.4374, 4.9055]	0.2074	2.5321 [1.2740, 3.7902]	3.6858 [2.4277, 4.9439]	0.2065
Nausea	38	59		38	61	
	2.8590 [1.7240, 3.9941]	3.3789 [2.4683, 4.2895]	0.4801	2.7657 [1.8324, 3.6990]	2.3099 [1.5745, 3.0453]	0.4497
Vomiting	11	16		11	13	
	3.0223 [1.4395, 4.6050]	2.7347 [1.4255, 4.0438]	0.7760	2.8424 [0.5548, 5.1300]	2.5179 [0.4257, 4.6101]	0.8349
Diarrhea	52	75		52	81	
	4.5566 [3.6849, 5.4283]	4.9407 [4.2156, 5.6659]	0.5048	4.5380 [3.6802, 5.3959]	5.3583 [4.6722, 6.0444]	0.1435

* Statistically significant difference was observed at the 5% significance level

Table 11. Secondary endpoint 2) ANCOVA results (mITT1)

Symptom	The placebo group vs The low dose group			The placebo group vs The high dose group		
	LS Mean [95% CI]		p-value	LS Mean [95% CI]		p-value
	The placebo	The low dose		The placebo	The high dose	
Fever	32	35		32	32	
	3.3347 [2.3370, 4.3323]	2.3512 [1.3973, 3.3050]	0.1597	3.2442 [2.0136, 4.4749]	3.3808 [2.1501, 4.6114]	0.8763
Cough	84	84		84	86	
	5.3688 [4.3733, 6.3642]	4.8931 [3.8977, 5.8886]	0.5059	5.3918 [4.3583, 6.4252]	5.9022 [4.8809, 6.9235]	0.4893
Sore throat	83	84		83	85	
	3.4647 [2.7718, 4.1577]	2.1420 [1.4532, 2.8308]	0.0084*	3.4474 [2.7801, 4.1147]	2.3279 [1.6685, 2.9872]	0.0198*
Headache	80	84		80	81	
	3.4845 [2.7044, 4.2647]	2.6754 [1.9141, 3.4367]	0.1451	3.4443 [2.6245, 4.2642]	2.9500 [2.1353, 3.7648]	0.4000
Muscle ache	80	80		80	75	
	1.8267 [1.4016, 2.2518]	1.4733 [1.0482, 1.8984]	0.2482	1.8093 [1.2993, 2.3193]	2.0701 [1.5432, 2.5969]	0.4850
Chill	67	72		67	64	
	1.3788 [0.9967, 1.7610]	1.2864 [0.9178, 1.6550]	0.7317	1.3653 [0.8706, 1.8599]	1.5082 [1.0020, 2.0144]	0.6914
Stuffy or runny nose	82	88		82	78	
	5.8816 [4.9310, 6.8322]	4.6785 [3.7609, 5.5960]	0.0743	5.9336 [4.9483, 6.9188]	5.9481 [4.9377, 6.9584]	0.9839
Fatigue	81	83		81	81	
	3.8772 [2.9930, 4.7614]	2.7644 [1.8910, 3.6379]	0.0792	3.8412 [2.9164, 4.7660]	4.3317 [3.4069, 5.2565]	0.4605
Difficulty of breathing	37	37		37	35	
	2.6536 [1.4372, 3.8699]	3.2924 [2.0760, 4.5087]	0.4620	2.6178 [1.3707, 3.8650]	3.3754 [2.0917, 4.6592]	0.4110
Nausea	35	53		35	54	
	2.6214 [1.5284, 3.7144]	3.0047 [2.1165, 3.8929]	0.5898	2.6022 [1.6595, 3.5448]	2.3134 [1.5561, 3.0707]	0.6377
Vomiting	10	13		10	12	
	2.1151 [0.7256, 3.5047]	1.6807 [0.4681, 2.8932]	0.6339	1.9466 [-0.3878, 4.2811]	2.7945 [0.6822, 4.9067]	0.5956
Diarrhea	48	68		48	74	

Symptom	The placebo group vs The low dose group			The placebo group vs The high dose group		
	LS Mean [95% CI]		p-value	LS Mean [95% CI]		p-value
	The placebo	The low dose		The placebo	The high dose	
	4.5123 [3.6336, 5.3911]	4.1972 [3.4604, 4.9340]	0.5893	4.4505 [3.5531, 5.3479]	5.2281 [4.5067 5.9494]	0.1856

* Statistically significant difference was observed at the 5% significance level

Table 12. Secondary endpoint 2) ANCOVA results (PPS)

Symptom	The placebo group vs The low dose group			The placebo group vs The high dose group		
	LS Mean [95% CI]		p-value	LS Mean [95% CI]		p-value
	The placebo	The low dose		The placebo	The high dose	
Fever	25	23		25	26	
	3.0996 [2.0396, 4.1596]	2.0874 [0.9821, 3.1927]	0.1905	3.0775 [1.6117, 4.5432]	3.2139 [1.7767, 4.6512]	0.8942
Cough	74	67		74	79	
	5.3958 [4.2978, 6.4937]	5.1151 [3.9611, 6.2691]	0.7282	5.4364 [4.2984, 6.5744]	5.8760 [4.7746, 6.9774]	0.5845
Sore throat	73	67		73	77	
	3.3687 [2.5825, 4.1549]	2.1132 [1.2925, 2.9340]	0.0308*	3.3485 [2.6147, 4.0824]	2.1890 [1.4746, 2.9035]	0.0269*
Headache	70	65		70	73	
	3.6468 [2.8053, 4.4883]	2.3419 [1.4686, 3.2152]	0.0353*	3.6659 [2.7947, 4.5370]	2.6423 [1.7893, 3.4954]	0.0993
Muscle ache	70	60		70	72	
	1.9980 [1.4842, 2.5118]	1.3523 [0.7972, 1.9074]	0.0940	1.9964 [1.4084, 2.5845]	1.8924 [1.3126, 2.4722]	0.8038
Chill	61	54		61	63	
	1.2403 [0.8996, 1.5810]	1.1174 [0.7552, 1.4797]	0.6263	1.2312 [0.7251, 1.7374]	1.4269 [0.9289, 1.9249]	0.5867
Stuffy or runny nose	73	69		73	73	
	5.8291 [4.7960, 6.8623]	4.6953 [3.6325, 5.7580]	0.1331	5.8477 [4.7997, 6.8956]	5.9126 [4.8647, 6.9605]	0.9312
Fatigue	74	65		74	74	
	3.9163 [2.9905, 4.8421]	2.2491 [1.2612, 3.2371]	0.0163*	3.9609 [2.9613, 4.9605]	4.2283 [3.2287, 5.2279]	0.7094
Difficulty of breathing	32	28		32	32	
	2.4899 [1.0772, 3.9025]	3.7794 [2.2692, 5.2897]	0.2170	2.4090 [0.9672, 3.8507]	3.6535 [2.2118, 5.0953]	0.2326
Nausea	31	37		31	53	

Symptom	The placebo group vs The low dose group			The placebo group vs The high dose group		
	LS Mean [95% CI]		p-value	LS Mean [95% CI]		p-value
	The placebo	The low dose		The placebo	The high dose	
	2.3897 [1.3120, 3.4673]	2.3087 [1.3228, 3.2946]	0.9124	2.3916 [1.3990, 3.3842]	2.1106 [1.3531, 2.8680]	0.6566
Vomiting	10	10		10	11	
	3.2535 [1.8310, 4.6759]	1.3465 [-0.0759, 2.7690]	0.0665	3.1891 [0.5858, 5.7925]	2.6008 [0.1276, 5.0739]	0.7431
Diarrhea	41	54		41	68	
	4.5656 [3.6295, 5.5017]	4.2280 [3.4133, 5.0427]	0.5918	4.5324 [3.5436, 5.5212]	5.1349 [4.3704, 5.8993]	0.3454

* Statistically significant difference was observed at the 5% significance level
† If the lower limit of the 95% CI for the LS mean is negative, it indicates that the estimate value is close to zero (same hereafter).

In the ITT analysis, the ANCOVA results showed a statistically significant difference at the 5% level in the time (days) taken for the improvement of sore throat symptoms between the placebo and the high dose group (p=0.0331). The LSMEAN [95% confidence interval] for this was 3.45 [2.79, 4.11] for the placebo group and 2.43 [1.77, 3.09] for the high dose group, indicating that the latter improved in sore throat symptoms 1.02 days faster than the former. No significant difference was observed between the low and the high dose groups and the placebo one. The LSMEAN [95% confidence interval] for the time taken for the improvement of sore throat symptoms for each group was 3.47 [2.75, 4.19] for the placebo group and 2.65 [1.92, 3.37] for the low dose group.

In the mITT1 analysis, ANCOVA results showed a statistically significant difference at the 5% level in the time taken for the improvement of sore throat symptoms between the placebo group and the low dose one (p=0.0084). The LSMEAN [95% confidence interval] for the time taken for the improvement of sore throat symptoms was 3.46 [2.77, 4.16] for the placebo group and 2.14 [1.45, 2.83] for the low dose one, indicating that the latter improved in sore throat symptoms 1.32 days faster than the former. significant difference was observed at the 5% level in the time taken for the improvement of sore throat symptoms between the placebo group and the high dose one (p=0.0198). The LSMEAN [95% confidence interval] for sore throat was 3.45 [2.78, 4.11] for the former and 2.33 [1.67, 2.99] for the latter, indicating that the high dose group improved in sore throat symptoms 1.12 days faster than the placebo one.

In the PPS analysis, the ANCOVA analysis showed a significant difference at the 5% significance level for symptoms of sore throat, headache, and fatigue between the placebo group and the low dose one. The latter improved symptoms faster than the former by 1.26 days for sore throat, 1.31 days for headache, and 1.67 days for fatigue. The LSMEAN [95% confidence interval] for the time taken for symptom improvement after sustaining for more than 48 hours for each symptom showing a significant difference between the placebo group and the low dose one were as follows:

Sore throat (p=0.0308): The placebo group 3.37 [2.58, 4.15], the low dose group 2.11 [1.29, 2.93]
Headache (p=0.0353): The placebo group 3.65 [2.81, 4.49], the low dose group 2.34 [1.47, 3.22]
Fatigue (p=0.0163): The placebo group 3.92 [2.99, 4.84], the low dose group 2.25 [1.26, 3.24]

Significant difference at the 5% significance level was showed in the time taken for sore throat symptoms to improve after sustaining for more than 48 hours between the placebo group and the high dose one (p=0.0269). The LSMEAN [95% confidence interval] for sore throat was 3.35 [2.61, 4.08] for the placebo group and 2.19 [1.47, 2.90] for the high dose one, indicating that the latter improved sore throat symptoms 1.16 days faster than the former.

3) Time (days) taken for each COVID-19 symptom score to improve by more than 1 point by Day 14:
Descriptive statistics for the time taken by Day 14 for each COVID-19 symptom score to decrease by more than one point for the placebo, the low dose, and the high dose groups are presented by symptom in Tables 13–15.
Participants not improved symptoms by the evening of Day 14.5 were censored to have a maximum improvement time as 13.00 days (maximum value 13 days = 14.5 days - (first day of administration + 0.5 day)).
ANCOVA analyses were conducted for each symptom, using age and severity as covariates, and the results are presented in Tables 16–18.

Table 13. Secondary endpoint 3) descriptive statistics (ITT)

Symptom		The placebo group N=98	The low dose group N=99	The high dose group N=96
Fever	N	9	12	7
	Mean	2.72	2.00	2.36
	SD	4.18	3.51	4.48
	Median	1.00	1.00	0.50
	Min	0.50	0.50	0.50
	Max	13.00	13.00	12.50
Cough	N	87	82	82
	Mean	4.67	5.20	5.21
	SD	4.70	4.93	4.83
	Median	2.00	3.25	3.00
	Min	0.50	-0.50*	0.50
	Max	13.00	13.00	13.00
Sore throat	N	86	88	85
	Mean	3.16	2.27	1.75
	SD	3.67	3.12	2.28
	Median	1.50	1.00	0.50
	Min	0.50	0.50	0.50
	Max	13.00	13.00	13.00
Headache	N	69	70	63
	Mean	2.28	1.96	2.21
	SD	3.09	2.57	3.22
	Median	1.00	1.00	0.50
	Min	0.50	0.50	0.50
	Max	13.00	13.00	13.00
Muscle ache	N	80	84	73
	Mean	1.78	1.38	1.46
	SD	2.39	2.03	2.19
	Median	0.50	0.50	0.50

Symptom		The placebo group N=98	The low dose group N=99	The high dose group N=96
	Min	0.50	-0.50*	0.50
	Max	13.00	13.00	12.50
Chill	N	63	66	57
	Mean	0.94	1.17	1.46
	SD	1.36	2.01	2.54
	Median	0.50	0.50	0.50
	Min	0.50	0.50	0.50
	Max	10.00	13.00	13.00
Stuffy or runny nose	N	66	70	64
	Mean	4.60	4.84	4.73
	SD	4.78	4.45	4.69
	Median	2.00	3.25	2.75
	Min	0.50	0.50	0.50
	Max	13.00	13.00	13.00
Fatigue	N	70	75	68
	Mean	2.69	1.87	2.91
	SD	3.50	3.18	3.86
	Median	1.00	0.50	1.00
	Min	0.50	0.50	0.50
	Max	13.00	13.00	13.00
Difficulty of breathing	N	21	23	24
	Mean	1.45	1.63	3.40
	SD	1.92	2.72	4.96
	Median	0.50	0.50	0.50
	Min	0.50	0.50	0.50
	Max	7.50	13.00	13.00
Nausea	N	21	27	37
	Mean	1.29	1.02	1.53
	SD	1.60	1.58	2.53
	Median	0.50	0.50	0.50
	Min	0.50	0.50	0.50
	Max	6.50	8.50	13.00
Vomiting	N	6	7	6
	Mean	0.75	1.57	0.92
	SD	0.27	1.69	0.58
	Median	0.75	0.50	0.75

Symptom		The placebo group N=98	The low dose group N=99	The high dose group N=96
	Min	0.50	0.50	0.50
	Max	1.00	5.00	2.00
Diarrhea	N	18	22	20
	Mean	2.89	3.27	4.33
	SD	3.08	3.11	3.02
	Median	0.75	2.75	4.50
	Min	0.50	0.50	0.50
	Max	9.50	13.00	13.00

* In low dose group, one participant (S02008) experienced an improvement of more than one point in cough and muscle ache in the morning of Day 2. However, as the first IP administration did not occur until the noon of Day 2 (resulting in a missed Day 1 night and Day 2 morning dose), the formula for calculating the time (days) required for symptom improvement resulted in a negative number. The calculation was based on the formula: (Day of symptom improvement observation - (First day of administration + 0.5 days)) = 2 (morning of Day 2) - (2 (Day 2 of first administration) + 0.5) = -0.5.

Table 14. Secondary endpoint 3) descriptive statistics (mITT1)

Symptom		The placebo group N=88	The low dose group N=89	The high dose group N=87
Fever	N	7	11	7
	Mean	3.29	1.05	2.36
	SD	4.64	0.57	4.48
	Median	1.00	1.00	0.50
	Min	0.50	0.50	0.50
	Max	13.00	2.50	12.50
Cough	N	77	74	74
	Mean	4.69	4.37	5.16
	SD	4.70	4.52	4.86
	Median	2.50	2.50	3.00
	Min	0.50	0.50	0.50
	Max	13.00	13.00	13.00
Sore throat	N	76	79	79
	Mean	3.16	1.90	1.64
	SD	3.60	2.59	1.96
	Median	1.50	1.00	0.50
	Min	0.50	0.50	0.50
	Max	13.00	13.00	13.00
Headache	N	65	63	60
	Mean	2.14	2.00	2.06

Symptom		The placebo group N=88	The low dose group N=89	The high dose group N=87
	SD	2.87	2.67	2.98
	Median	1.00	1.00	0.50
	Min	0.50	0.50	0.50
	Max	12.50	13.00	13.00
Muscle ache	N	72	76	66
	Mean	1.65	1.29	1.53
	SD	2.07	1.90	2.29
	Median	0.50	0.50	0.50
	Min	0.50	0.50	0.50
	Max	9.00	13.00	12.50
Chill	N	56	63	54
	Mean	0.98	1.03	1.47
	SD	1.43	1.44	2.52
	Median	0.50	0.50	0.50
	Min	0.50	0.50	0.50
	Max	10.00	10.50	13.00
Stuffy or runny nose	N	58	63	59
	Mean	4.45	3.86	4.70
	SD	4.71	3.75	4.73
	Median	2.00	2.50	2.50
	Min	0.50	0.50	0.50
	Max	13.00	13.00	13.00
Fatigue	N	64	69	62
	Mean	2.52	1.66	3.06
	SD	3.32	2.86	4.01
	Median	1.00	0.50	1.00
	Min	0.50	0.50	0.50
	Max	13.00	13.00	13.00
Difficulty of breathing	N	19	23	22
	Mean	1.55	1.63	2.64
	SD	1.99	2.72	4.42
	Median	0.50	0.50	0.50
	Min	0.50	0.50	0.50
	Max	7.50	13.00	13.00
Nausea	N	18	24	34
	Mean	1.17	1.00	1.59

Symptom		The placebo group N=88	The low dose group N=89	The high dose group N=87
	SD	1.46	1.66	2.61
	Median	0.50	0.50	0.50
	Min	0.50	0.50	0.50
	Max	6.50	8.50	13.00
Vomiting	N	6	4	6
	Mean	0.75	1.25	0.92
	SD	0.27	0.96	0.58
	Median	0.75	1.00	0.75
	Min	0.50	0.50	0.50
	Max	1.00	2.50	2.00
Diarrhea	N	17	21	18
	Mean	3.03	3.10	4.39
	SD	3.12	3.07	3.17
	Median	1.00	2.50	4.75
	Min	0.50	0.50	0.50
	Max	9.50	13.00	13.00

Table 15. Secondary endpoint 3) descriptive statistics (PPS)

Symptom		The placebo group N=77	The low dose group N=70	The high dose group N=80
Fever	N	5	9	7
	Mean	3.10	1.06	2.36
	SD	5.54	0.63	4.48
	Median	0.50	1.00	0.50
	Min	0.50	0.50	0.50
	Max	13.00	2.50	12.50
Cough	N	69	61	72
	Mean	4.89	4.37	5.16
	SD	4.91	4.44	4.93
	Median	2.50	2.50	2.75
	Min	0.50	0.50	0.50
	Max	13.00	13.00	13.00
Sore throat	N	68	66	73
	Mean	3.09	1.86	1.60
	SD	3.77	2.78	1.97
	Median	1.50	0.50	0.50
	Min	0.50	0.50	0.50

Symptom		The placebo group N=77	The low dose group N=70	The high dose group N=80
	Max	13.00	13.00	13.00
Headache	N	59	52	55
	Mean	2.35	1.87	1.82
	SD	3.16	2.69	2.74
	Median	1.00	1.00	0.50
	Min	0.50	0.50	0.50
	Max	13.00	13.00	13.00
Muscle ache	N	64	57	64
	Mean	1.90	1.15	1.38
	SD	2.58	1.79	2.13
	Median	0.50	0.50	0.50
	Min	0.50	0.50	0.50
	Max	13.00	13.00	12.50
Chill	N	55	48	53
	Mean	0.96	0.84	1.34
	SD	1.44	0.71	2.46
	Median	0.50	0.50	0.50
	Min	0.50	0.50	0.50
	Max	10.00	4.00	13.00
Stuffy or runny nose	N	54	57	57
	Mean	4.30	4.23	4.82
	SD	4.75	4.03	4.65
	Median	1.75	3.00	3.00
	Min	0.50	0.50	0.50
	Max	13.00	13.00	13.00
Fatigue	N	60	60	60
	Mean	2.69	1.48	3.08
	SD	3.65	2.67	4.02
	Median	1.00	0.50	1.00
	Min	0.50	0.50	0.50
	Max	13.00	13.00	13.00
Difficulty of breathing	N	19	20	22
	Mean	1.53	1.78	3.09
	SD	2.00	2.89	4.70
	Median	0.50	0.50	0.50
	Min	0.50	0.50	0.50

Symptom		The placebo group N=77	The low dose group N=70	The high dose group N=80
	Max	7.50	13.00	13.00
Nausea	N	17	22	35
	Mean	0.79	0.68	1.51
	SD	0.59	0.39	2.58
	Median	0.50	0.50	0.50
	Min	0.50	0.50	0.50
	Max	2.50	2.00	13.00
Vomiting	N	5	6	5
	Mean	0.80	1.75	0.70
	SD	0.27	1.78	0.27
	Median	1.00	1.00	0.50
	Min	0.50	0.50	0.50
	Max	1.00	5.00	1.00
Diarrhea	N	16	21	20
	Mean	3.19	3.40	4.33
	SD	3.15	3.12	3.02
	Median	1.75	3.00	4.50
	Min	0.50	0.50	0.50
	Max	9.50	13.00	13.00

Table 16. Secondary endpoint 3) ANCOVA results (ITT)

Symptom	The placebo group vs The low dose group			The placebo group vs The high dose group		
	LS Mean [95% CI]		p-value	LS Mean [95% CI]		p-value
	The placebo	The low dose		The placebo	The high dose	
Fever	9	12		9	7	
	2.8042 [-0.0832, 5.6917]	1.9385 [-0.5435, 4.4205]	0.6458	3.1307 [-0.3622, 6.6237]	1.8319 [-2.2070, 5.8708]	0.6307
Cough	87	82		87	82	
	4.6847 [3.6697, 5.6997]	5.1881 [4.1426, 6.2337]	0.4964	4.6754 [3.6607, 5.6901]	5.2042 [4.1589, 6.2494]	0.4748
Sore throat	86	88		86	85	
	3.1813 [2.4544, 3.9082]	2.2489 [1.5304, 2.9675]	0.0740	3.1569 [2.5024, 3.8114]	1.7471 [1.0888, 2.4055]	0.0032*
Headache	69	70		69	63	
	2.2903 [1.6240, 2.9566]	1.9496 [1.2881, 2.6111]	0.4751	2.2877 [1.5377, 3.0376]	2.2088 [1.4239, 2.9936]	0.8859
Muscle ache	80	84		80	73	

Symptom	The placebo group vs The low dose group			The placebo group vs The high dose group		
	LS Mean [95% CI]		p-value	LS Mean [95% CI]		p-value
	The placebo	The low dose		The placebo	The high dose	
	1.8351 [1.3526, 2.3177]	1.3296 [0.8588, 1.8005]	0.1420	1.8422 [1.3399, 2.3444]	1.3921 [0.8661, 1.9182]	0.2259
Chill	63	66		63	57	
	0.9713 [0.5611, 1.3815]	1.1334 [0.7327, 1.5342]	0.5773	0.9441 [0.4396, 1.4485]	1.4478 [0.9174, 1.9782]	0.1763
Stuffy or runny nose	66	70		66	64	
	4.6199 [3.4890, 5.7508]	4.8227 [3.7248, 5.9205]	0.8003	4.6651 [3.5032, 5.8269]	4.6657 [3.4857, 5.8458]	0.9994
Fatigue	70	75		70	68	
	2.7518 [1.9811, 3.5225]	1.8050 [1.0606, 2.5495]	0.0834	2.7056 [1.8382, 3.5730]	2.8913 [2.0112, 3.7713]	0.7670
Difficulty of breathing	21	23		21	24	
	1.5455 [0.6318, 2.4593]	1.5454 [0.6725, 2.4183]	0.9998	1.3062 [-0.4486, 3.0610]	3.5237 [1.8861, 5.1613]	0.0740
Nausea	21	27		21	37	
	1.3012 [0.5810, 2.0214]	1.0065 [0.3738, 1.6391]	0.5448	1.3071 [0.2985, 2.3156]	1.5149 [0.7586, 2.2712]	0.7442
Vomiting	6	7		6	6	
	0.9233 [-0.0294, 1.8760]	1.4229 [0.5425, 2.3033]	0.4162	0.7710 [0.4766, 1.0653]	0.8957 [0.6013, 1.1900]	0.5608
Diarrhea	18	22		18	20	
	2.9046 [1.4354, 4.3738]	3.2599 [1.9328, 4.5869]	0.7199	2.9042 [1.4238, 4.3846]	4.3112 [2.9090, 5.7134]	0.1761

* Statistically significant difference was observed at the 5% significance level

Table 17. Secondary endpoint 1) ANCOVA results (mITT1)

Symptom	The placebo group vs The low dose group			The placebo group vs The high dose group		
	LS Mean [95% CI]		p-value	LS Mean [95% CI]		p-value
	The placebo	The low dose		The placebo	The high dose	
Fever	7	11		7	7	
	3.1928 [0.7771, 5.6086]	1.1046 [-0.8073, 3.0164]	0.1747	3.6646 [-0.7352, 8.0644]	1.9782 [-2.4216, 6.3780]	0.5866
Cough	77	74		77	74	
	4.7157 [3.6883, 5.7431]	4.3431 [3.2950, 5.3912]	0.6170	4.6909 [3.6076, 5.7742]	5.1527 [4.0477, 6.2578]	0.5563
Sore throat	76	79		76	79	

Symptom	The placebo group vs The low dose group			The placebo group vs The high dose group		
	LS Mean [95% CI]		p-value	LS Mean [95% CI]		p-value
	The placebo	The low dose		The placebo	The high dose	
	3.1901 [2.4831, 3.8972]	1.8677 [1.1742, 2.5612]	0.0093*	3.1672 [2.5095, 3.8249]	1.6303 [0.9852, 2.2754]	0.0012*
Headache	65	63		65	60	
	2.1609 [1.4884, 2.8335]	1.9768 [1.2936, 2.6600]	0.7050	2.1433 [1.4289, 2.8578]	2.0531 [1.3094, 2.7967]	0.8627
Muscle ache	72	76		72	66	
	1.6977 [1.2378, 2.1576]	1.2403 [0.7928, 1.6879]	0.1631	1.6813 [1.1761, 2.1865]	1.4916 [0.9636, 2.0196]	0.6107
Chill	56	63		56	54	
	1.0100 [0.6359, 1.3841]	1.0070 [0.6544, 1.3596]	0.9908	0.9946 [0.4494, 1.5399]	1.4593 [0.9039, 2.0146]	0.2403
Stuffy or runny nose	58	63		58	59	
	4.4843 [3.3730, 5.5957]	3.8239 [2.7580, 4.8899]	0.3995	4.5557 [3.3198, 5.7915]	4.5978 [3.3727, 5.8230]	0.9621
Fatigue	64	69		64	62	
	2.5812 [1.8228, 3.3395]	1.6059 [0.8757, 2.3361]	0.0700	2.5352 [1.6270, 3.4435]	3.0443 [2.1215, 3.9671]	0.4388
Difficulty of breathing	19	23		19	22	
	1.6106 [0.6266, 2.5946]	1.5825 [0.6883, 2.4768]	0.9662	1.3904 [-0.2857, 3.0666]	2.7764 [1.2223, 4.3306]	0.2339
Nausea	18	24		18	34	
	1.1687 [0.3787, 1.9587]	0.9985 [0.3191, 1.6778]	0.7484	1.2549 [0.1338, 2.3761]	1.5415 [0.7338, 2.3493]	0.6829
Vomiting	6	4		6	6	
	0.7502 [0.1047, 1.3956]	1.2498 [0.4590, 2.0405]	0.2855	0.7710 [0.4766, 1.0653]	0.8957 [0.6013, 1.1900]	0.5608
Diarrhea	17	21		17	18	
	3.0486 [1.5341, 4.5632]	3.0797 [1.7199, 4.4395]	0.9757	3.1046 [1.5247, 4.6844]	4.3179 [2.7844, 5.8515]	0.2788

* Statistically significant difference was observed at the 5% significance level

Table 18. Secondary endpoint 1) ANCOVA results (PPS)

Symptom	The placebo group vs The low dose group			The placebo group vs The high dose group		
	LS Mean [95% CI]		p-value	LS Mean [95% CI]		p-value
	The placebo	The low dose		The placebo	The high dose	
Fever	5	9		5	7	

Symptom	The placebo group vs The low dose group			The placebo group vs The high dose group		
	LS Mean [95% CI]		p-value	LS Mean [95% CI]		p-value
	The placebo	The low dose		The placebo	The high dose	
	3.3615 [0.0059, 6.7170]	0.9103 [-1.5582, 3.3788]	0.2307	3.8793 [-1.7259, 9.4845]	1.8005 [-2.8183, 6.4193]	0.5604
Cough	69	61		69	72	
	4.8783 [3.7568, 5.9999]	4.3835 [3.1906, 5.5764]	0.5510	4.9011 [3.7211, 6.0810]	5.1504 [3.9953, 6.3054]	0.7660
Sore throat	68	66		68	73	
	3.1212 [2.3269, 3.9154]	1.8297 [1.0234, 2.6360]	0.0259	3.0833 [2.3633, 3.8033]	1.6005 [0.9057, 2.2954]	0.0040
Headache	59	52		59	55	
	2.3447 [1.5955, 3.0940]	1.8685 [1.0704, 2.6666]	0.3905	2.3655 [1.6027, 3.1283]	1.7988 [1.0087, 2.5889]	0.3089
Muscle ache	64	57		64	64	
	1.9290 [1.3783, 2.4797]	1.1148 [0.5310, 1.6985]	0.0475	1.9258 [1.3449, 2.5068]	1.3554 [0.7745, 1.9364]	0.1731
Chill	55	48		55	53	
	0.9659 [0.6524, 1.2795]	0.8411 [0.5054, 1.1768]	0.5918	0.9638 [0.4237, 1.5039]	1.3395 [0.7892, 1.8897]	0.3364
Stuffy or runny nose	54	57		54	57	
	4.3414 [3.1444, 5.5384]	4.1853 [3.0205, 5.3501]	0.8539	4.3569 [3.0935, 5.6202]	4.7672 [3.5378, 5.9966]	0.6469
Fatigue	60	60		60	60	
	2.7467 [1.9309, 3.5625]	1.4199 [0.6041, 2.2358]	0.0251	2.7492 [1.7730, 3.7253]	3.0258 [2.0497, 4.0020]	0.6928
Difficulty of breathing	19	20		19	22	
	1.6339 [0.6085, 2.6593]	1.6728 [0.6735, 2.6721]	0.9564	1.3550 [-0.4231, 3.1330]	3.2389 [1.5907, 4.8871]	0.1301
Nausea	17	22		17	35	
	0.7914 [0.5422, 1.0407]	0.6839 [0.4659, 0.9019]	0.5219	0.8091 [-0.2821, 1.9002]	1.5070 [0.7536, 2.2604]	0.3007
Vomiting	5	6		5	5	
	1.0059 [-0.2005, 2.2122]	1.5784 [0.4805, 2.6764]	0.4488	0.6991 [0.4099, 0.9883]	0.8009 [0.5117, 1.0901]	0.6097
Diarrhea	16	21		16	20	
	3.4706 [2.0569, 4.8842]	3.1891 [1.9597, 4.4184]	0.7648	3.0623 [1.4397, 4.6850]	4.4251 [2.9820, 5.8683]	0.2213

* Statistically significant difference was observed at the 5% significance level

In the ITT analysis, the ANCOVA analysis indicated that sore throat showed the most significant difference between the groups at a 5% significance level (p=0.0740). The LSMEAN [95% confidence interval] for the time (days) taken for a decrease of more than 1 point in sore throat symptoms was 3.18 [2.45, 3.91] days for the placebo group and 2.25 [1.53, 2.97] days for the low dose group. Significant differences were observed at the 5% significance level in the time (days) taken for a decrease of more than 1 point in sore throat symptoms between the placebo and The high dose group (p=0.0032), with LSMEANs of 3.16 [2.50, 3.81] days for The placebo group and 1.75 [1.09, 2.41] days for the high dose group, indicating a 1.41 days faster improvement in the high dose group.

For mITT1, a significant difference at the 5% significance level was observed in sore throat symptom between the placebo and the low dose group (p=0.0093). The time (days) for a symptom decrease of more than 1 point in sore throat was significantly shorter in low dose group, with LSMEANs of 3.19 [2.48, 3.90] for The placebo group and 1.87 [1.17, 2.56] for the low dose group, indicating 1.32 days faster decrease. A significant difference was found between the placebo and The high dose group (p=0.0012), with LSMEANs of 3.17 [2.51, 3.82] for the placebo group and 1.63 [0.99, 2.28] for The high dose group, showing a 1.54 days faster improvement in sore throat symptoms for The high dose group.

For PPS analysis, ANCOVA results showed statistically significant differences at the 5% level for symptoms of sore throat, muscle ache, and fatigue between the placebo and the low dose group, which showed faster symptom improvement than The placebo group by 1.29 days for sore throat, 0.82 days for muscle pain, and 1.33 days for fatigue. The LSMEAN [95% confidence intervals] for the time (days) taken for a decrease of more than one point in these symptoms were as follows:

Sore throat (p=0.0259): The placebo group 3.12 [2.33, 3.92], The low dose group 1.83 [1.02, 2.64]

Muscle ache (p=0.0475): The placebo group 1.93 [1.38, 2.48], The low dose group 1.11 [0.53, 1.70]

Fatigue (p=0.0251): The placebo group 2.75 [1.93, 3.56], The low dose group 1.42 [0.60, 2.24]

In the placebo and the high dose group, the time taken for the sore throat symptom to decrease by more than 1 point showed a significant difference at the 5% significance level (p=0.0040). The LSMEAN [95% CI] for the sore throat symptom improvement was 3.08 [2.36, 3.80] for the placebo group and 1.60 [0.91, 2.30] for the high dose group, indicating that the latter experienced a sore throat symptom improvement 1.48 days faster than the former.

4) Total and average all COVID-19 symptom scores change by Day 14

For the placebo, the low dose, and the high dose groups, descriptive statistics for the total and average changes in COVID-19 symptom scores by Day 14 are presented in Tables 19–22. Changes were new as the difference from each participant's baseline values, excluding symptoms that were not present.

Total scores and averages were analyzed for each day, using baseline values, age, and severity as covariates in an ANCOVA analysis. The results for the total scores and averages by day are presented in Tables 23–26.

In the ITT analysis, both the total and average scores for the placebo and the low dose group consistently decreased, with significant differences observed at certain points for total scores. The ANCOVA analysis for total score changes showed a significant difference at Day 4 at the 5% significance level (p=0.0293), with the LSMEAN [95% CI] for the placebo and the low dose group showing 4.01 [3.32, 4.71] and 5.11 [4.42, 5.80], respectively. No significant differences were observed between the placebo and the low dose group across all days for average score changes. The lowest p-value for differences was observed on Day 4 (p=0.0523), with the LSMEAN [95% CI] being 1.10 [1.01, 1.19] for the placebo and 1.22 [1.13, 1.30] for the low dose group.

In the placebo and the high dose group, both total scores and averages consistently decreased, and significant differences between the two groups were observed at certain points. The ANCOVA analysis for the change in total scores showed significant differences between the groups at Days 2.5, 3.5, 4, and 4.5 with a significance level of 5%, however, no statistically significant differences were observed on other days. The LSMEANs [95% confidence intervals] for the days where significant differences were as follows:

Day 2.5 (p=0.0497): The placebo group 7.32 [6.47, 8.17], The high dose group 8.53 [7.67, 9.39]

Day 3.5 (p=0.0476): The placebo group 5.10 [4.44, 5.76], The high dose group 6.05 [5.38, 6.71]

Day 4 (p=0.0182): The placebo group 4.02 [3.48, 4.56], The high dose group 4.95 [4.40, 5.49]

Day 4.5 (p=0.0150): The placebo group 3.55 [2.99, 4.12], The high dose group 4.55 [3.98, 5.11]
Significant differences for average score changes at the 5% significance level were observed on Days 4, 4.5, and 6.5 between the placebo and The high dose group. Significant differences and their LSMEAN [95% CI] are as follows:
Day 4 (p=0.0500): The placebo group 1.10 [1.03, 1.17], The high dose group 1.20 [1.13, 1.28]
Day 4.5 (p=0.0079): The placebo group 1.04 [0.97, 1.12], The high dose group 1.19 [1.12, 1.27]
Day 6.5 (p=0.0408): The placebo group 0.92 [0.82, 1.01], The high dose group 1.06 [0.96, 1.15]

In the PPS analysis, total and average scores for the placebo and the low dose group also consistently decreased, with significant differences observed at certain points for average scores. No significant differences were found for total score changes at the 5% significance level. The lowest p-value for differences was observed on Day 7 (p=0.0764), with the LSMEAN [95% CI] for the placebo and the low dose group showing 2.55 [2.07, 3.03] and 1.91 [1.41, 2.42], respectively. Significant differences for average score changes at the 5% significance level were observed on Days 7, 7.5, 9, 9.5, 10, 10.5, and 11 between the placebo and the low dose group. Significant differences and their LSMEAN [95% CI] are as follows:
Day 7 (p=0.0212): The placebo group 0.94 [0.84, 1.05], The low dose group 0.76 [0.65, 0.87]
Day 7.5 (p=0.0096): The placebo group 0.88 [0.76, 0.99], The low dose group 0.66 [0.54, 0.78]
Day 9 (p=0.0199): The placebo group 0.83 [0.71, 0.96], The low dose group 0.62 [0.50, 0.75]
Day 9.5 (p=0.0127): The placebo group 0.83 [0.71, 0.95], The low dose group 0.60 [0.48, 0.73]
Day 10 (p=0.0073): The placebo group 0.80 [0.69, 0.92], The low dose group 0.57 [0.44, 0.69]
Day 10.5 (p=0.0033): The placebo group 0.80 [0.68, 0.92], The low dose group 0.53 [0.41, 0.66]
Day 11 (p=0.0471): The placebo group 0.76 [0.64, 0.88], The low dose group 0.59 [0.46, 0.71]
In the placebo and the high dose group, both total scores and averages were observed to decrease consistently, and significant differences between the two groups were identified at certain points. The ANCOVA analysis for the change in total scores revealed a significant difference between the groups on Day 4.5 (p=0.0227), with LSMEANs [95% confidence intervals] of 3.36 [2.74, 3.97] for the placebo group and 4.36 [3.76, 4.96] for the high dose group. The analysis for the change in averages showed significant differences between the placebo and the high dose group on Days 4, 4.5, 5, and 6.5, with a 5% significance level. The LSMEANs [95% confidence intervals] for the days where significant differences were observed are as follows:
Day 4 (p=0.0401): The placebo group 1.09 [1.00, 1.17], The high dose group 1.21 [1.13, 1.29]
Day 4.5 (p=0.0096): The placebo group 1.01 [0.92, 1.10], The high dose group 1.17 [1.09, 1.26]
Day 5 (p=0.0268): The placebo group 0.98 [0.88, 1.08], The high dose group 1.13 [1.04, 1.23]
Day 6.5 (p=0.0310): The placebo group 0.88 [0.78, 0.98], The high dose group 1.04 [0.94, 1.14]

Table 19. Secondary endpoint 4) descriptive statistics for the change in total symptom score (ITT)

Category		The placebo group<br>N=98		The low dose group<br>N=99		The high dose group<br>N=96	
		Values<br>(Day#)	Change<br>(Day# - baseline)	Values<br>(Day#)	Change<br>(Day# - baseline)	Values<br>(Day#)	Change<br>(Day# - baseline)
Baseline	N	98	NA	99	NA	96	NA
	Mean±SD	11.39±4.93	NA	11.96±5.54	NA	11.58±5.33	NA
	Median<br>[Min, Max]	10.50<br>[4.00, 25.00]	NA	11.00<br>[4.00, 26.00]	NA	11.00<br>[4.00, 24.00]	NA
Day 2	N	98	98	99	99	96	96
	Mean±SD	9.66±4.74	-1.72±5.10	9.54±4.42	-2.42±6.15	9.90±4.88	-1.69±5.96
	Median<br>[Min, Max]	9.00<br>[1.00, 22.00]	-2.00<br>[-15.00, 13.00]	9.00<br>[1.00, 24.00]	-3.00<br>[-21.00, 17.00]	9.00<br>[2.00, 34.00]	-2.00<br>[-13.00, 29.00]
Day 2.5	N	98	98	99	99	96	96
	Mean±SD	7.28±4.07	-4.11±5.20	8.15±4.13	-3.81±6.02	8.57±4.90	-3.01±5.98
	Median<br>[Min, Max]	6.50<br>[0.00, 20.00]	-4.00<br>[-16.00, 13.00]	8.00<br>[1.00, 21.00]	-4.00<br>[-18.00, 14.00]	7.50<br>[2.00, 28.00]	-3.00<br>[-17.00, 23.00]
Day 3	N	98	98	99	99	96	96
	Mean±SD	6.10±3.62	-5.29±5.09	6.76±4.16	-5.20±6.60	6.72±3.80	-4.86±5.50
	Median<br>[Min, Max]	5.00<br>[0.00, 19.00]	-5.00<br>[-17.00, 12.00]	6.00<br>[1.00, 22.00]	-5.00<br>[-19.00, 14.00]	6.00<br>[1.00, 19.00]	-5.00<br>[-19.00, 14.00]
Day 3.5	N	98	98	99	99	96	96
	Mean±SD	5.06±3.59	-6.33±5.17	5.79±4.18	-6.17±6.74	6.08±3.44	-5.50±5.17
	Median<br>[Min, Max]	4.00<br>[0.00, 18.00]	-6.50<br>[-20.00, 11.00]	5.00<br>[0.00, 24.00]	-6.00<br>[-20.00, 16.00]	5.00<br>[0.00, 16.00]	-5.50<br>[-18.00, 9.00]
Day 4	N	98	98	99	99	96	96
	Mean±SD	4.00±2.69	-7.39±5.22	5.12±4.11	-6.84±6.94	4.97±2.99	-6.61±4.96
	Median	3.00	-7.00	4.00	-7.00	4.00	-7.00

Category		The placebo group N=98		The low dose group N=99		The high dose group N=96	
		Values (Day#)	Change (Day# - baseline)	Values (Day#)	Change (Day# - baseline)	Values (Day#)	Change (Day# - baseline)
	[Min, Max]	[0.00, 11.00]	[-25.00, 4.00]	[0.00, 25.00]	[-20.00, 17.00]	[0.00, 14.00]	[-19.00, 7.00]
Day 4.5	N	98	98	99	99	96	96
	Mean±SD	3.53±2.80	-7.86±5.35	4.26±3.57	-7.70±6.81	4.57±2.94	-7.01±5.45
	Median [Min, Max]	3.00 [0.00, 13.00]	-8.00 [-22.00, 4.00]	4.00 [0.00, 17.00]	-7.00 [-23.00, 9.00]	4.00 [0.00, 13.00]	-7.00 [-20.00, 7.00]
Day 5	N	98	98	99	99	96	96
	Mean±SD	3.07±2.61	-8.32±5.25	3.74±3.32	-8.22±6.61	3.82±2.71	-7.76±5.55
	Median [Min, Max]	2.00 [0.00, 13.00]	-8.00 [-25.00, 2.00]	3.00 [0.00, 16.00]	-8.00 [-23.00, 10.00]	3.00 [0.00, 13.00]	-8.00 [-20.00, 5.00]
Day 5.5	N	98	98	99	99	96	96
	Mean±SD	2.76±2.68	-8.63±5.26	3.07±3.03	-8.89±6.38	3.51±2.78	-8.07±5.36
	Median [Min, Max]	2.00 [0.00, 13.00]	-8.00 [-23.00, 2.00]	2.00 [0.00, 16.00]	-9.00 [-24.00, 9.00]	3.00 [0.00, 16.00]	-8.00 [-21.00, 3.00]
Day 6	N	98	98	99	99	96	96
	Mean±SD	2.36±2.23	-9.03±5.39	2.90±3.56	-9.06±6.80	2.89±2.67	-8.70±5.28
	Median [Min, Max]	2.00 [0.00, 10.00]	-9.00 [-25.00, 1.00]	2.00 [0.00, 22.00]	-9.00 [-24.00, 15.00]	2.00 [0.00, 14.00]	-8.50 [-21.00, 3.00]
Day 6.5	N	98	98	99	99	96	96
	Mean±SD	2.65±2.34	-8.73±5.48	2.98±3.47	-8.98±6.85	3.26±2.71	-8.32±5.21
	Median [Min, Max]	2.00 [0.00, 10.00]	-8.00 [-23.00, 2.00]	2.00 [0.00, 20.00]	-8.00 [-24.00, 13.00]	3.00 [0.00, 13.00]	-8.00 [-21.00, 3.00]
Day 7	N	98	98	99	99	96	96
	Mean±SD	2.63±2.10	-8.76±5.41	2.54±2.76	-9.42±6.43	2.63±2.14	-8.96±5.04
	Median	2.50	-8.00	2.00	-9.00	2.00	-9.00

Category		The placebo group N=98		The low dose group N=99		The high dose group N=96	
		Values (Day#)	Change (Day# - baseline)	Values (Day#)	Change (Day# - baseline)	Values (Day#)	Change (Day# - baseline)
	[Min, Max]	[0.00, 11.00]	[-23.00, 0.00]	[0.00, 16.00]	[-24.00, 9.00]	[0.00, 11.00]	[-21.00, 2.00]
Day 7.5	N	98	98	99	99	96	96
	Mean±SD	2.44±2.17	-8.95±5.44	2.31±2.78	-9.65±6.43	2.57±2.18	-9.01±5.18
	Median [Min, Max]	2.00 [0.00, 10.00]	-8.00 [-23.00, 4.00]	2.00 [0.00, 16.00]	-9.00 [-25.00, 9.00]	2.00 [0.00, 13.00]	-8.50 [-20.00, 1.00]
Day 8	N	98	98	99	99	96	96
	Mean±SD	2.12±2.01	-9.27±5.32	2.25±2.63	-9.71±6.40	2.14±1.91	-9.45±5.15
	Median [Min, Max]	2.00 [0.00, 8.00]	-8.50 [-23.00, 2.00]	1.00 [0.00, 15.00]	-9.00 [-25.00, 9.00]	2.00 [0.00, 10.00]	-9.00 [-21.00, -1.00]
Day 8.5	N	98	98	99	99	96	96
	Mean±SD	1.98±2.00	-9.41±5.35	2.07±2.66	-9.89±6.50	2.17±1.87	-9.42±5.06
	Median [Min, Max]	1.00 [0.00, 8.00]	-9.00 [-23.00, 1.00]	1.00 [0.00, 15.00]	-10.00 [-25.00, 10.00]	2.00 [0.00, 9.00]	-9.00 [-20.00, -1.00]
Day 9	N	98	98	99	99	96	96
	Mean±SD	2.06±2.02	-9.33±5.15	2.05±2.67	-9.91±6.48	1.94±1.95	-9.65±5.16
	Median [Min, Max]	2.00 [0.00, 10.00]	-9.00 [-23.00, 0.00]	1.00 [0.00, 15.00]	-9.00 [-25.00, 10.00]	2.00 [0.00, 11.00]	-9.00 [-20.00, -1.00]
Day 9.5	N	98	98	99	99	96	96
	Mean±SD	1.96±1.96	-9.43±5.14	2.02±2.71	-9.94±6.49	1.98±1.83	-9.60±5.13
	Median [Min, Max]	1.00 [0.00, 10.00]	-9.00 [-23.00, -1.00]	1.00 [0.00, 15.00]	-9.00 [-25.00, 8.00]	2.00 [0.00, 8.00]	-9.00 [-21.00, -1.00]
Day 10	N	98	98	99	99	96	96
	Mean±SD	1.85±1.81	-9.54±4.97	1.80±2.67	-10.16±6.61	1.83±1.86	-9.75±5.13
	Median	2.00	-9.00	1.00	-9.00	1.00	-9.50

Category		The placebo group N=98		The low dose group N=99		The high dose group N=96	
		Values (Day#)	Change (Day# - baseline)	Values (Day#)	Change (Day# - baseline)	Values (Day#)	Change (Day# - baseline)
	[Min, Max]	[0.00, 11.00]	[-23.00, -2.00]	[0.00, 15.00]	[-26.00, 10.00]	[0.00, 9.00]	[-21.00, -1.00]
Day 10.5	N	98	98	99	99	96	96
	Mean±SD	1.88±1.87	-9.51±4.99	1.78±2.68	-10.18±6.64	1.82±1.90	-9.76±5.21
	Median [Min, Max]	1.00 [0.00, 10.00]	-9.00 [-23.00, 0.00]	1.00 [0.00, 15.00]	-9.00 [-26.00, 9.00]	1.00 [0.00, 11.00]	-9.00 [-21.00, -1.00]
Day 11	N	98	98	99	99	96	96
	Mean±SD	1.70±1.87	-9.68±5.07	1.85±2.67	-10.11±6.62	1.60±1.78	-9.98±5.17
	Median [Min, Max]	1.00 [0.00, 9.00]	-9.00 [-23.00, -1.00]	1.00 [0.00, 16.00]	-9.00 [-26.00, 9.00]	1.00 [0.00, 11.00]	-10.00 [-21.00, -1.00]
Day 11.5	N	98	98	99	99	96	96
	Mean±SD	1.63±1.76	-9.76±5.04	1.76±2.62	-10.20±6.59	1.64±1.65	-9.95±5.12
	Median [Min, Max]	1.00 [0.00, 8.00]	-9.00 [-23.00, -1.00]	1.00 [0.00, 17.00]	-10.00 [-26.00, 10.00]	1.00 [0.00, 8.00]	-10.00 [-21.00, -1.00]
Day 12	N	98	98	99	99	96	96
	Mean±SD	1.47±1.52	-9.92±5.21	1.73±2.68	-10.23±6.64	1.55±1.76	-10.03±5.08
	Median [Min, Max]	1.00 [0.00, 7.00]	-9.00 [-25.00, -1.00]	1.00 [0.00, 15.00]	-10.00 [-26.00, 11.00]	1.00 [0.00, 11.00]	-10.00 [-20.00, -1.00]
Day 12.5	N	98	98	99	99	96	96
	Mean±SD	1.54±1.82	-9.85±5.01	1.71±2.48	-10.25±6.49	1.52±1.60	-10.06±5.24
	Median [Min, Max]	1.00 [0.00, 10.00]	-9.00 [-23.00, -1.00]	1.00 [0.00, 14.00]	-9.00 [-26.00, 7.00]	1.00 [0.00, 8.00]	-9.50 [-22.00, -1.00]
Day 13	N	98	98	99	99	96	96
	Mean±SD	1.50±1.84	-9.89±4.95	1.54±2.46	-10.42±6.45	1.48±1.92	-10.10±5.17
	Median	1.00	-9.00	1.00	-10.00	1.00	-10.00

Category		The placebo group N=98		The low dose group N=99		The high dose group N=96	
		Values (Day#)	Change (Day# - baseline)	Values (Day#)	Change (Day# - baseline)	Values (Day#)	Change (Day# - baseline)
	[Min, Max]	[0.00, 10.00]	[-23.00, -1.00]	[0.00, 14.00]	[-26.00, 7.00]	[0.00, 14.00]	[-21.00, -1.00]
Day 13.5	N	98	98	99	99	96	96
	Mean±SD	1.39±1.90	-10.00±4.95	1.53±2.46	-10.43±6.37	1.63±2.58	-9.96±5.49
	Median [Min, Max]	1.00 [0.00, 11.00]	-9.00 [-23.00, -1.00]	1.00 [0.00, 14.00]	-10.00 [-26.00, 7.00]	1.00 [0.00, 22.00]	-10.00 [-22.00, 7.00]
Day 14	N	98	98	99	99	96	96
	Mean±SD	1.28±1.88	-10.11±4.86	1.52±2.43	-10.44±6.43	1.42±2.05	-10.17±5.08
	Median [Min, Max]	1.00 [0.00, 12.00]	-9.00 [-23.00, -2.00]	1.00 [0.00, 14.00]	-10.00 [-26.00, 7.00]	1.00[0.00, 12.00]	-10.00 [-21.00, -1.00]
Day 14.5	N	98	98	99	99	96	96
	Mean±SD	1.40±1.92	-9.99±4.86	1.47±2.48	-10.48±6.46	1.44±1.97	-10.15±5.18
	Median [Min, Max]	1.00 [0.00, 11.00]	-9.00 [-23.00, -1.00]	1.00 [0.00, 15.00]	-10.00 [-26.00, 8.00]	1.00 [0.00, 12.00]	-10.00 [-21.00, -1.00]

Table 20. Secondary endpoint 4) descriptive statistics for the change in total symptom score (PPS)

Category		The placebo group N=77		The low dose group N=70		The high dose group N=80	
		Values (Day#)	Change (Day# - baseline)	Values (Day#)	Change (Day# - baseline)	Values (Day#)	Change (Day# - baseline)
Baseline	N	77	NA	70	NA	80	NA
	Mean±SD	11.75±4.67	NA	13.10±5.73	NA	12.40±5.24	NA
	Median [Min, Max]	11.00 [5.00, 25.00]	NA	13.00 [4.00, 26.00]	NA	12.50 [4.00, 24.00]	NA
Day 2	N	77	77	70	70	80	80
	Mean±SD	9.64±4.41	-2.12±4.93	9.21±3.78	-3.89±5.37	9.68±4.21	-2.73±4.54

Category		The placebo group N=77		The low dose group N=70		The high dose group N=80	
		Values (Day#)	Change (Day# - baseline)	Values (Day#)	Change (Day# - baseline)	Values (Day#)	Change (Day# - baseline)
	Median [Min, Max]	9.00 [3.00, 22.00]	-2.00 [-15.00, 11.00]	9.00 [2.00, 20.00]	-3.00 [-21.00, 9.00]	9.00 [2.00, 20.00]	-3.00 [-13.00, 7.00]
Day 2.5	N	77	77	70	70	80	80
	Mean±SD	7.10±3.74	-4.65±5.12	7.61±3.73	-5.49±4.98	8.48±4.64	-3.93±5.18
	Median [Min, Max]	7.00 [0.00, 19.00]	-5.00 [-16.00, 8.00]	8.00 [1.00, 19.00]	-5.00 [-18.00, 8.00]	7.50 [2.00, 25.00]	-4.00 [-17.00, 11.00]
Day 3	N	77	77	70	70	80	80
	Mean±SD	5.91±3.42	-5.84±4.87	6.03±3.44	-7.07±5.55	6.41±3.67	-5.99±4.91
	Median [Min, Max]	5.00 [0.00, 16.00]	-6.00 [-17.00, 7.00]	6.00 [1.00, 17.00]	-7.00 [-19.00, 7.00]	6.00 [1.00, 16.00]	-5.00 [-19.00, 3.00]
Day 3.5	N	77	77	70	70	80	80
	Mean±SD	4.96±3.50	-6.79±4.96	5.03±3.35	-8.07±5.74	5.99±3.55	-6.41±4.90
	Median [Min, Max]	4.00 [0.00, 16.00]	-7.00 [-20.00, 3.00]	5.00 [0.00, 14.00]	-7.50 [-20.00, 10.00]	5.00 [0.00, 16.00]	-6.00 [-18.00, 8.00]
Day 4	N	77	77	70	70	80	80
	Mean±SD	3.87±2.68	-7.88±5.13	4.29±3.28	-8.81±5.99	4.79±3.05	-7.61±4.56
	Median [Min, Max]	3.00 [0.00, 11.00]	-7.00 [-25.00, 4.00]	4.00 [0.00, 14.00]	-8.00 [-20.00, 9.00]	4.00 [0.00, 14.00]	-8.00 [-19.00, 4.00]
Day 4.5	N	77	77	70	70	80	80
	Mean±SD	3.29±2.65	-8.47±5.09	3.49±2.71	-9.61±6.13	4.43±2.95	-7.98±5.05
	Median [Min, Max]	3.00 [0.00, 9.00]	-8.00 [-22.00, 3.00]	3.00 [0.00, 13.00]	-9.00 [-23.00, 9.00]	4.00 [0.00, 13.00]	-8.00 [-20.00, 7.00]
Day 5	N	77	77	70	70	80	80
	Mean±SD	2.87±2.44	-8.88±5.12	3.09±2.74	-10.01±6.12	3.76±2.76	-8.64±5.29

Category		The placebo group<br>N=77		The low dose group<br>N=70		The high dose group<br>N=80	
		Values<br>(Day#)	Change<br>(Day# - baseline)	Values<br>(Day#)	Change<br>(Day# - baseline)	Values<br>(Day#)	Change<br>(Day# - baseline)
	Median<br>[Min, Max]	2.00<br>[0.00, 10.00]	-9.00<br>[-25.00, 2.00]	3.00<br>[0.00, 14.00]	-10.00<br>[-23.00, 10.00]	3.00<br>[0.00, 13.00]	-9.00<br>[-20.00, 5.00]
Day 5.5	N	77	77	70	70	80	80
	Mean±SD	2.55±2.46	-9.21±5.13	2.46±2.42	-10.64±5.92	3.43±2.89	-8.98±5.10
	Median<br>[Min, Max]	2.00<br>[0.00, 11.00]	-9.00<br>[-23.00, 2.00]	2.00<br>[0.00, 13.00]	-10.00<br>[-24.00, 9.00]	3.00<br>[0.00, 16.00]	-9.50<br>[-21.00, 3.00]
Day 6	N	77	77	70	70	80	80
	Mean±SD	2.12±2.05	-9.64±5.13	2.36±2.77	-10.74±6.21	2.89±2.71	-9.51±5.09
	Median<br>[Min, Max]	2.00<br>[0.00, 8.00]	-9.00<br>[-25.00, 0.00]	2.00<br>[0.00, 16.00]	-11.00<br>[-24.00, 12.00]	2.00<br>[0.00, 14.00]	-10.00<br>[-21.00, 3.00]
Day 6.5	N	77	77	70	70	80	80
	Mean±SD	2.47±2.16	-9.29±5.11	2.13±2.52	-10.97±6.23	3.10±2.68	-9.30±4.83
	Median<br>[Min, Max]	2.00<br>[0.00, 8.00]	-9.00<br>[-23.00, 1.00]	1.50<br>[0.00, 15.00]	-10.50<br>[-24.00, 11.00]	2.00<br>[0.00, 13.00]	-9.00<br>[-21.00, 0.00]
Day 7	N	77	77	70	70	80	80
	Mean±SD	2.55±1.96	-9.21±5.16	1.91±2.24	-11.19±6.09	2.61±2.22	-9.79±4.88
	Median<br>[Min, Max]	3.00<br>[0.00, 8.00]	-8.00<br>[-23.00, 0.00]	1.00<br>[0.00, 12.00]	-10.50<br>[-24.00, 8.00]	2.00<br>[0.00, 11.00]	-10.00<br>[-21.00, 0.00]
Day 7.5	N	77	77	70	70	80	80
	Mean±SD	2.31±2.00	-9.44±5.06	1.81±2.39	-11.29±6.18	2.49±2.27	-9.91±4.95
	Median<br>[Min, Max]	2.00<br>[0.00, 8.00]	-8.00<br>[-23.00, 1.00]	1.00<br>[0.00, 13.00]	-11.00<br>[-25.00, 9.00]	2.00<br>[0.00, 13.00]	-10.00<br>[-20.00, 0.00]
Day 8	N	77	77	70	70	80	80
	Mean±SD	2.16±1.98	-9.60±5.06	1.74±2.21	-11.36±6.21	2.09±2.03	-10.31±4.95

Category		The placebo group N=77		The low dose group N=70		The high dose group N=80	
		Values (Day#)	Change (Day# - baseline)	Values (Day#)	Change (Day# - baseline)	Values (Day#)	Change (Day# - baseline)
	Median [Min, Max]	2.00 [0.00, 7.00]	-9.00 [-23.00, 0.00]	1.00 [0.00, 13.00]	-10.00 [-25.00, 9.00]	2.00 [0.00, 10.00]	-10.00 [-21.00, -2.00]
Day 8.5	N	77	77	70	70	80	80
	Mean±SD	2.01±1.98	-9.74±5.14	1.59±2.16	-11.51±6.31	2.15±1.98	-10.25±4.86
	Median [Min, Max]	1.00 [0.00, 7.00]	-9.00 [-23.00, 1.00]	1.00 [0.00, 14.00]	-11.00 [-25.00, 10.00]	2.00 [0.00, 9.00]	-10.00 [-20.00, -1.00]
Day 9	N	77	77	70	70	80	80
	Mean±SD	2.18±2.08	-9.57±4.92	1.56±2.27	-11.54±6.31	1.91±2.06	-10.49±4.99
	Median [Min, Max]	2.00 [0.00, 10.00]	-9.00 [-23.00, 0.00]	1.00 [0.00, 14.00]	-11.00 [-25.00, 10.00]	1.50 [0.00, 11.00]	-10.00 [-20.00, -2.00]
Day 9.5	N	77	77	70	70	80	80
	Mean±SD	2.04±2.02	-9.71±4.92	1.49±2.15	-11.61±6.25	1.99±1.91	-10.41±4.97
	Median [Min, Max]	1.00 [0.00, 10.00]	-9.00 [-23.00, -1.00]	1.00 [0.00, 12.00]	-11.00 [-25.00, 8.00]	2.00 [0.00, 8.00]	-10.00 [-21.00, -1.00]
Day 10	N	77	77	70	70	80	80
	Mean±SD	1.90±1.86	-9.86±4.67	1.33±2.17	-11.77±6.43	1.81±1.91	-10.59±4.96
	Median [Min, Max]	2.00 [0.00, 11.00]	-9.00 [-23.00, -2.00]	1.00 [0.00, 14.00]	-11.00 [-26.00, 10.00]	1.00 [0.00, 9.00]	-10.00 [-21.00, -2.00]
Day 10.5	N	77	77	70	70	80	80
	Mean±SD	1.94±1.93	-9.82±4.76	1.30±2.23	-11.80±6.46	1.83±1.96	-10.58±5.10
	Median [Min, Max]	2.00 [0.00, 10.00]	-9.00 [-23.00, 0.00]	0.50 [0.00, 13.00]	-11.00 [-26.00, 9.00]	1.00 [0.00, 11.00]	-10.00 [-21.00, -2.00]
Day 11	N	77	77	70	70	80	80
	Mean±SD	1.79±1.93	-9.96±4.79	1.37±2.19	-11.73±6.45	1.63±1.88	-10.78±5.06

Category		The placebo group N=77		The low dose group N=70		The high dose group N=80	
		Values (Day#)	Change (Day# - baseline)	Values (Day#)	Change (Day# - baseline)	Values (Day#)	Change (Day# - baseline)
	Median [Min, Max]	1.00 [0.00, 9.00]	-9.00 [-23.00, -1.00]	1.00 [0.00, 12.00]	-11.00 [-26.00, 8.00]	1.00 [0.00, 11.00]	-10.00 [-21.00, -2.00]
Day 11.5	N	77	77	70	70	80	80
	Mean±SD	1.66±1.81	-10.09±4.73	1.36±2.15	-11.74±6.46	1.64±1.68	-10.76±4.97
	Median [Min, Max]	1.00 [0.00, 8.00]	-9.00 [-23.00, -1.00]	1.00 [0.00, 12.00]	-11.00 [-26.00, 8.00]	1.00 [0.00, 8.00]	-10.50 [-21.00, -2.00]
Day 12	N	77	77	70	70	80	80
	Mean±SD	1.48±1.52	-10.27±4.90	1.39±2.42	-11.71±6.66	1.54±1.80	-10.86±4.91
	Median [Min, Max]	1.00 [0.00, 7.00]	-9.00 [-25.00, -2.00]	1.00 [0.00, 15.00]	-11.00 [-26.00, 11.00]	1.00 [0.00, 11.00]	-10.50 [-20.00, -2.00]
Day 12.5	N	77	77	70	70	80	80
	Mean±SD	1.60±1.86	-10.16±4.65	1.36±2.16	-11.74±6.48	1.50±1.62	-10.90±5.09
	Median [Min, Max]	1.00 [0.00, 10.00]	-9.00 [-23.00, -2.00]	1.00 [0.00, 11.00]	-11.50 [-26.00, 7.00]	1.00 [0.00, 8.00]	-10.50 [-22.00, -3.00]
Day 13	N	77	77	70	70	80	80
	Mean±SD	1.55±1.90	-10.21±4.55	1.24±2.00	-11.86±6.43	1.48±2.02	-10.93±5.04
	Median [Min, Max]	1.00 [0.00, 10.00]	-9.00 [-23.00, -2.00]	1.00 [0.00, 11.00]	-11.00 [-26.00, 7.00]	1.00 [0.00, 14.00]	-10.50 [-21.00, -1.00]
Day 13.5	N	77	77	70	70	80	80
	Mean±SD	1.51±1.94	-10.25±4.53	1.23±2.01	-11.87±6.31	1.64±2.74	-10.76±5.45
	Median [Min, Max]	1.00 [0.00, 11.00]	-9.00 [-23.00, -2.00]	1.00 [0.00, 10.00]	-11.50 [-26.00, 6.00]	1.00 [0.00, 22.00]	-10.50 [-22.00, 7.00]
Day 14	N	77	77	70	70	80	80
	Mean±SD	1.40±1.99	-10.35±4.46	1.14±1.89	-11.96±6.34	1.46±2.15	-10.94±4.99

Category		The placebo group N=77		The low dose group N=70		The high dose group N=80	
		Values (Day#)	Change (Day# - baseline)	Values (Day#)	Change (Day# - baseline)	Values (Day#)	Change (Day# - baseline)
	Median [Min, Max]	1.00 [0.00, 12.00]	-9.00 [-23.00, -2.00]	0.50 [0.00, 9.00]	-11.50 [-26.00, 5.00]	1.00 [0.00, 12.00]	-10.50 [-21.00, -3.00]
Day 14.5	N	77	77	70	70	80	80
	Mean±SD	1.48±1.98	-10.27±4.42	1.14±2.09	-11.96±6.43	1.46±2.01	-10.94±5.08
	Median [Min, Max]	1.00 [0.00, 11.00]	-9.00 [-23.00, -3.00]	0.00 [0.00, 12.00]	-11.50 [-26.00, 8.00]	1.00 [0.00, 12.00]	-10.50 [-21.00, -2.00]

Table 21. Secondary endpoint 4) descriptive statistics for the change in average symptom score (ITT)

Category		The placebo group N=98		The low dose group N=99		The high dose group N=96	
		Values (Day#)	Change (Day# - baseline)	Values (Day#)	Change (Day# - baseline)	Values (Day#)	Change (Day# - baseline)
Baseline	N	98	NA	99	NA	96	NA
	Mean±SD	1.88±0.38	NA	1.88±0.34	NA	1.90±0.36	NA
	Median [Min, Max]	1.83 [1.29, 3.00]	NA	1.86 [1.29, 3.00]	NA	1.95 [1.29, 3.00]	NA
Day 2	N	98	98	99	99	96	96
	Mean±SD	1.52±0.37	-0.36±0.43	1.47±0.34	-0.41±0.45	1.50±0.37	-0.40±0.43
	Median [Min, Max]	1.50 [1.00, 2.50]	-0.33 [-1.50, 0.51]	1.43 [1.00, 2.38]	-0.42 [-1.67, 0.67]	1.50 [1.00, 2.83]	-0.39 [-1.33, 1.17]
Day 2.5	N	98	98	99	99	96	96
	Mean±SD	1.36±0.39	-0.52±0.46	1.37±0.31	-0.51±0.39	1.42±0.39	-0.48±0.42
	Median [Min, Max]	1.33 [0.00, 2.38]	-0.50 [-1.75, 0.60]	1.33 [1.00, 2.50]	-0.50 [-1.43, 0.50]	1.33[1.00, 2.50]	-0.52 [-1.50, 0.67]
Day 3	N	98	98	99	99	96	96

Category		The placebo group<br>N=98		The low dose group<br>N=99		The high dose group<br>N=96	
		Values<br>(Day#)	Change<br>(Day# - baseline)	Values<br>(Day#)	Change<br>(Day# - baseline)	Values<br>(Day#)	Change<br>(Day# - baseline)
	Mean±SD	1.30±0.39	-0.58±0.50	1.31±0.33	-0.57±0.43	1.29±0.30	-0.60±0.41
	Median<br>[Min, Max]	1.25<br>[0.00, 3.00]	-0.57<br>[-2.29, 0.75]	1.25<br>[1.00, 2.44]	-0.56<br>[-1.56, 0.67]	1.25<br>[1.00, 2.33]	-0.54<br>[-1.75, 0.19]
Day 3.5	N	98	98	99	99	96	96
	Mean±SD	1.23±0.43	-0.65±0.56	1.23±0.38	-0.65±0.49	1.25±0.29	-0.65±0.45
	Median<br>[Min, Max]	1.00<br>[0.00, 3.00]	-0.60<br>[-2.33, 1.29]	1.17<br>[0.00, 2.40]	-0.63<br>[-2.00, 0.50]	1.27<br>[0.00, 2.00]	-0.57<br>[-2.00, 0.40]
Day 4	N	98	98	99	99	96	96
	Mean±SD	1.10±0.41	-0.78±0.53	1.22±0.45	-0.66±0.53	1.21±0.31	-0.69±0.42
	Median<br>[Min, Max]	1.00<br>[0.00, 2.33]	-0.71<br>[-2.50, 0.33]	1.17<br>[0.00, 2.60]	-0.63<br>[-2.00, 0.60]	1.00<br>[0.00, 2.00]	-0.65<br>[-2.00, 0.13]
Day 4.5	N	98	98	99	99	96	96
	Mean±SD	1.04±0.45	-0.84±0.58	1.13±0.46	-0.75±0.54	1.19±0.31	-0.70±0.44
	Median<br>[Min, Max]	1.00<br>[0.00, 2.00]	-0.74<br>[-2.80, 0.20]	1.00<br>[0.00, 2.60]	-0.75<br>[-2.00, 0.60]	1.00<br>[0.00, 2.17]	-0.67<br>[-2.00, 0.21]
Day 5	N	98	98	99	99	96	96
	Mean±SD	1.01±0.49	-0.87±0.63	1.01±0.49	-0.87±0.56	1.13±0.34	-0.76±0.48
	Median<br>[Min, Max]	1.00<br>[0.00, 2.33]	-0.71<br>[-2.80, 0.60]	1.00<br>[0.00, 2.33]	-0.86<br>[-2.14, 0.42]	1.00<br>[0.00, 2.00]	-0.67<br>[-2.29, 0.22]
Day 5.5	N	98	98	99	99	96	96
	Mean±SD	1.01±0.53	-0.87±0.67	0.98±0.52	-0.90±0.57	1.08±0.38	-0.82±0.52
	Median<br>[Min, Max]	1.00<br>[0.00, 2.33]	-0.73<br>[-2.80, 0.60]	1.00<br>[0.00, 2.60]	-0.83<br>[-2.14, 0.67]	1.00<br>[0.00, 2.00]	-0.67<br>[-2.63, 0.10]
Day 6	N	98	98	99	99	96	96

Category		The placebo group N=98		The low dose group N=99		The high dose group N=96	
		Values (Day#)	Change (Day# - baseline)	Values (Day#)	Change (Day# - baseline)	Values (Day#)	Change (Day# - baseline)
	Mean±SD	0.91±0.55	-0.97±0.69	0.91±0.53	-0.97±0.59	0.94±0.54	-0.96±0.66
	Median [Min, Max]	1.00[0.00, 2.00]	-0.92 [-2.80, 0.60]	1.00 [0.00, 2.29]	-0.98 [-2.14, 0.67]	1.00 [0.00, 2.00]	-0.81 [-3.00, 0.60]
Day 6.5	N	98	98	99	99	96	96
	Mean±SD	0.91±0.48	-0.97±0.63	0.90±0.55	-0.98±0.62	1.06±0.48	-0.84±0.56
	Median [Min, Max]	1.00[0.00, 2.67]	-0.89 [-2.88, 0.92]	1.00 [0.00, 2.17]	-1.00 [-2.14, 0.25]	1.00 [0.00, 2.50]	-0.70 [-2.63, 0.50]
Day 7	N	98	98	99	99	96	96
	Mean±SD	0.95±0.44	-0.93±0.61	0.88±0.52	-1.00±0.61	0.96±0.47	-0.93±0.56
	Median [Min, Max]	1.00 [0.00, 2.50]	-0.75 [-2.88, 0.75]	1.00 [0.00, 2.00]	-1.00 [-2.14, 0.67]	1.00 [0.00, 2.00]	-0.87 [-3.00, 0.00]
Day 7.5	N	98	98	99	99	96	96
	Mean±SD	0.90±0.47	-0.98±0.63	0.77±0.57	-1.11±0.65	0.97±0.47	-0.93±0.56
	Median [Min, Max]	1.00 [0.00, 2.50]	-0.85 [-2.88, 0.75]	1.00 [0.00, 2.17]	-1.00 [-2.56, 0.50]	1.00 [0.00, 2.00]	-0.85 [-3.00, 0.00]
Day 8	N	98	98	99	99	96	96
	Mean±SD	0.83±0.54	-1.05±0.69	0.79±0.57	-1.09±0.65	0.89±0.50	-1.01±0.56
	Median [Min, Max]	1.00 [0.00, 3.00]	-1.00 [-2.88, 1.50]	1.00 [0.00, 2.60]	-1.00 [-2.56, 0.60]	1.00 [0.00, 2.00]	-1.00 [-3.00, 0.40]
Day 8.5	N	98	98	99	99	96	96
	Mean±SD	0.79±0.52	-1.09±0.69	0.75±0.57	-1.13±0.66	0.94±0.55	-0.95±0.61
	Median [Min, Max]	1.00 [0.00, 2.00]	-1.00 [-2.88, 0.50]	1.00 [0.00, 2.25]	-1.00 [-2.56, 0.50]	1.00 [0.00, 3.00]	-0.89 [-3.00, 0.67]
Day 9	N	98	98	99	99	96	96

Category		The placebo group N=98		The low dose group N=99		The high dose group N=96	
		Values (Day#)	Change (Day# - baseline)	Values (Day#)	Change (Day# - baseline)	Values (Day#)	Change (Day# - baseline)
	Mean±SD	0.82±0.55	-1.06±0.69	0.73±0.58	-1.15±0.67	0.84±0.57	-1.06±0.63
	Median [Min, Max]	1.00 [0.00, 3.00]	-1.00 [-2.88, 0.71]	1.00 [0.00, 2.00]	-1.00 [-2.56, 0.50]	1.00 [0.00, 3.00]	-1.00 [-3.00, 0.67]
Day 9.5	N	98	98	99	99	96	96
	Mean±SD	0.80±0.52	-1.08±0.67	0.70±0.57	-1.18±0.65	0.89±0.56	-1.01±0.64
	Median [Min, Max]	1.00 [0.00, 2.00]	-1.00 [-2.88, 0.50]	1.00 [0.00, 2.25]	-1.14 [-2.56, 0.50]	1.00 [0.00, 3.00]	-1.00 [-3.00, 0.67]
Day 10	N	98	98	99	99	96	96
	Mean±SD	0.79±0.52	-1.09±0.66	0.66±0.58	-1.22±0.65	0.79±0.55	-1.11±0.62
	Median [Min, Max]	1.00 [0.00, 2.20]	-1.00 [-2.88, 0.50]	1.00 [0.00, 2.25]	-1.25 [-2.60, 0.50]	1.00 [0.00, 2.00]	-1.00 [-3.00, 0.00]
Day 10.5	N	98	98	99	99	96	96
	Mean±SD	0.79±0.52	-1.09±0.65	0.64±0.59	-1.24±0.68	0.81±0.54	-1.09±0.65
	Median [Min, Max]	1.00 [0.00, 2.00]	-1.00 [-2.88, 0.25]	1.00 [0.00, 2.17]	-1.25 [-2.60, 0.50]	1.00 [0.00, 2.00]	-1.00 [-3.00, 0.00]
Day 11	N	98	98	99	99	96	96
	Mean±SD	0.73±0.52	-1.15±0.67	0.68±0.59	-1.20±0.68	0.74±0.54	-1.15±0.64
	Median [Min, Max]	1.00 [0.00, 2.00]	-1.00 [-2.88, 0.25]	1.00 [0.00, 2.67]	-1.20 [-2.60, 0.67]	1.00 [0.00, 2.00]	-1.00 [-3.00, 0.00]
Day 11.5	N	98	98	99	99	96	96
	Mean±SD	0.74±0.56	-1.14±0.70	0.68±0.60	-1.20±0.69	0.76±0.58	-1.14±0.68
	Median [Min, Max]	1.00 [0.00, 2.00]	-1.00 [-2.88, 0.38]	1.00 [0.00, 2.67]	-1.20 [-2.60, 0.67]	1.00 [0.00, 3.00]	-1.00 [-3.00, 1.00]
Day 12	N	98	98	99	99	96	96

Category		The placebo group N=98		The low dose group N=99		The high dose group N=96	
		Values (Day#)	Change (Day# - baseline)	Values (Day#)	Change (Day# - baseline)	Values (Day#)	Change (Day# - baseline)
	Mean±SD	0.70±0.55	-1.18±0.71	0.65±0.59	-1.23±0.68	0.73±0.54	-1.16±0.63
	Median [Min, Max]	1.00 [0.00, 2.00]	-1.06 [-2.88, 0.38]	1.00 [0.00, 2.67]	-1.22 [-2.60, 0.67]	1.00 [0.00, 2.00]	-1.00 [-3.00, 0.00]
Day 12.5	N	98	98	99	99	96	96
	Mean±SD	0.67±0.55	-1.21±0.67	0.69±0.65	-1.19±0.72	0.76±0.59	-1.13±0.71
	Median [Min, Max]	1.00 [0.00, 2.00]	-1.17 [-2.88, 0.25]	1.00 [0.00, 2.75]	-1.22 [-2.60, 0.75]	1.00 [0.00, 3.00]	-1.00 [-3.00, 1.00]
Day 13	N	98	98	99	99	96	96
	Mean±SD	0.66±0.56	-1.22±0.64	0.62±0.60	-1.26±0.70	0.69±0.59	-1.20±0.67
	Median [Min, Max]	1.00 [0.00, 2.00]	-1.25 [-2.88, 0.25]	1.00 [0.00, 2.75]	-1.25 [-2.60, 0.75]	1.00 [0.00, 2.00]	-1.19 [-3.00, 0.14]
Day 13.5	N	98	98	99	99	96	96
	Mean±SD	0.60±0.60	-1.28±0.72	0.62±0.60	-1.26±0.70	0.71±0.57	-1.19±0.66
	Median [Min, Max]	1.00 [0.00, 2.20]	-1.33 [-2.88, 0.50]	1.00 [0.00, 2.25]	-1.25 [-2.67, 0.50]	1.00 [0.00, 2.44]	-1.10 [-3.00, 0.57]
Day 14	N	98	98	99	99	96	96
	Mean±SD	0.59±0.59	-1.29±0.68	0.62±0.58	-1.26±0.67	0.65±0.57	-1.25±0.65
	Median [Min, Max]	1.00 [0.00, 2.33]	-1.33 [-2.88, 0.52]	1.00 [0.00, 2.25]	-1.25 [-2.60, 0.50]	1.00 [0.00, 2.00]	-1.25 [-3.00, 0.00]
Day 14.5	N	98	98	99	99	96	96
	Mean±SD	0.64±0.62	-1.24±0.70	0.61±0.59	-1.27±0.70	0.69±0.62	-1.21±0.69
	Median [Min, Max]	1.00 [0.00, 2.33]	-1.31 [-2.88, 0.52]	1.00 [0.00, 2.00]	-1.25 [-2.67, 0.50]	1.00 [0.00, 3.00]	-1.20 [-3.00, 1.00]

Table 22. Secondary endpoint 4) descriptive statistics for the change in average symptom score (PPS)

Category		The placebo group N=77		The low dose group N=70		The high dose group N=80	
		Values (Day#)	Change (Day# - baseline)	Values (Day#)		Values (Day#)	Change (Day# - baseline)
Baseline	N	77	NA	70	NA	80	NA
	Mean±SD	1.86±0.40	NA	1.89±0.34	NA	1.90±0.38	NA
	Median [Min, Max]	1.80 [1.29, 3.00]	NA	1.87 [1.33, 2.67]	NA	1.88 [1.29, 3.00]	NA
Day 2	N	77	77	70	70	80	80
	Mean±SD	1.47±0.34	-0.38±0.45	1.45±0.33	-0.44±0.43	1.48±0.35	-0.42±0.40
	Median [Min, Max]	1.50 [1.00, 2.29]	-0.34 [-1.50, 0.51]	1.44 [1.00, 2.25]	-0.47 [-1.67, 0.67]	1.50 [1.00, 2.60]	-0.39 [-1.33, 1.03]
Day 2.5	N	77	77	70	70	80	80
	Mean±SD	1.32±0.37	-0.54±0.49	1.31±0.28	-0.58±0.36	1.41±0.38	-0.48±0.40
	Median [Min, Max]	1.29 [0.00, 2.33]	-0.50 [-1.75, 0.60]	1.29 [1.00, 2.00]	-0.58 [-1.43, 0.30]	1.33 [1.00, 2.50]	-0.52 [-1.50, 0.52]
Day 3	N	77	77	70	70	80	80
	Mean±SD	1.28±0.40	-0.57±0.51	1.25±0.29	-0.64±0.39	1.29±0.30	-0.61±0.41
	Median [Min, Max]	1.20 [0.00, 3.00]	-0.55 [-2.29, 0.75]	1.17 [1.00, 2.00]	-0.63 [-1.56, 0.67]	1.25 [1.00, 2.33]	-0.53 [-1.75, 0.19]
Day 3.5	N	77	77	70	70	80	80
	Mean±SD	1.22±0.44	-0.64±0.57	1.17±0.38	-0.72±0.47	1.26±0.31	-0.64±0.47
	Median [Min, Max]	1.00 [0.00, 3.00]	-0.57 [-2.33, 1.29]	1.06 [0.00, 2.33]	-0.67 [-2.00, 0.33]	1.33 [0.00, 2.00]	-0.57 [-2.00, 0.40]
Day 4	N	77	77	70	70	80	80
	Mean±SD	1.08±0.41	-0.77±0.55	1.16±0.47	-0.73±0.54	1.21±0.32	-0.68±0.43
	Median [Min, Max]	1.00 [0.00, 2.33]	-0.71 [-2.50, 0.29]	1.00 [0.00, 2.60]	-0.75 [-2.00, 0.60]	1.00 [0.00, 2.00]	-0.63 [-2.00, 0.13]

Category		The placebo group N=77		The low dose group N=70		The high dose group N=80	
		Values (Day#)	Change (Day# - baseline)	Values (Day#)		Values (Day#)	Change (Day# - baseline)
Day 4.5	N	77	77	70	70	80	80
	Mean±SD	1.00±0.46	-0.85±0.60	1.07±0.50	-0.82±0.57	1.18±0.30	-0.72±0.45
	Median [Min, Max]	1.00 [0.00, 2.00]	-0.75 [-2.80, 0.20]	1.00 [0.00, 2.60]	-0.85 [-2.00, 0.60]	1.00 [0.00, 2.17]	-0.67 [-2.00, 0.17]
Day 5	N	77	77	70	70	80	80
	Mean±SD	0.97±0.50	-0.88±0.67	0.97±0.53	-0.92±0.60	1.14±0.36	-0.76±0.50
	Median [Min, Max]	1.00 [0.00, 2.33]	-0.75 [-2.80, 0.60]	1.00 [0.00, 2.33]	-0.87 [-2.14, 0.42]	1.00 [0.00, 2.00]	-0.67 [-2.29, 0.22]
Day 5.5	N	77	77	70	70	80	80
	Mean±SD	0.98±0.54	-0.88±0.70	0.92±0.54	-0.98±0.59	1.06±0.40	-0.83±0.54
	Median [Min, Max]	1.00 [0.00, 2.33]	-0.75 [-2.80, 0.60]	1.00 [0.00, 2.60]	-0.86 [-2.14, 0.67]	1.00 [0.00, 2.00]	-0.67 [-2.63, 0.10]
Day 6	N	77	77	70	70	80	80
	Mean±SD	0.86±0.54	-0.99±0.70	0.84±0.56	-1.05±0.60	0.93±0.54	-0.97±0.68
	Median [Min, Max]	1.00 [0.00, 2.00]	-1.00 [-2.80, 0.60]	1.00 [0.00, 2.29]	-1.00 [-2.14, 0.67]	1.00 [0.00, 2.00]	-0.79 [-3.00, 0.60]
Day 6.5	N	77	77	70	70	80	80
	Mean±SD	0.87±0.43	-0.99±0.61	0.78±0.53	-1.11±0.60	1.04±0.49	-0.85±0.59
	Median [Min, Max]	1.00 [0.00, 1.67]	-0.83 [-2.88, -0.13]	1.00 [0.00, 2.14]	-1.00 [-2.14, 0.14]	1.00 [0.00, 2.50]	-0.70 [-2.63, 0.50]
Day 7	N	77	77	70	70	80	80
	Mean±SD	0.94±0.41	-0.92±0.60	0.76±0.52	-1.13±0.58	0.93±0.50	-0.96±0.60
	Median [Min, Max]	1.00 [0.00, 2.00]	-0.75 [-2.88, -0.07]	1.00 [0.00, 2.00]	-1.00 [-2.14, 0.67]	1.00 [0.00, 2.00]	-0.83 [-3.00, 0.00]

Category		The placebo group<br>N=77		The low dose group<br>N=70		The high dose group<br>N=80	
		Values<br>(Day#)	Change<br>(Day# - baseline)	Values<br>(Day#)		Values<br>(Day#)	Change<br>(Day# - baseline)
Day 7.5	N	77	77	70	70	80	80
	Mean±SD	0.87±0.44	-0.98±0.63	0.66±0.55	-1.23±0.61	0.95±0.49	-0.95±0.58
	Median<br>[Min, Max]	1.00<br>[0.00, 1.50]	-0.83<br>[-2.88, 0.00]	1.00<br>[0.00, 2.17]	-1.25<br>[-2.56, 0.17]	1.00<br>[0.00, 2.00]	-0.83<br>[-3.00, 0.00]
Day 8	N	77	77	70	70	80	80
	Mean±SD	0.84±0.53	-1.02±0.71	0.70±0.57	-1.19±0.64	0.85±0.54	-1.04±0.59
	Median<br>[Min, Max]	1.00<br>[0.00, 3.00]	-0.95<br>[-2.88, 1.50]	1.00<br>[0.00, 2.60]	-1.22<br>[-2.56, 0.60]	1.00<br>[0.00, 2.00]	-1.00<br>[-3.00, 0.40]
Day 8.5	N	77	77	70	70	80	80
	Mean±SD	0.79±0.52	-1.07±0.71	0.68±0.53	-1.21±0.61	0.92±0.58	-0.97±0.63
	Median<br>[Min, Max]	1.00<br>[0.00, 2.00]	-1.00<br>[-2.88, 0.50]	1.00<br>[0.00, 2.00]	-1.21<br>[-2.56, 0.00]	1.00<br>[0.00, 3.00]	-0.87<br>[-3.00, 0.67]
Day 9	N	77	77	70	70	80	80
	Mean±SD	0.84±0.50	-1.02±0.69	0.62±0.57	-1.27±0.64	0.80±0.60	-1.09±0.65
	Median<br>[Min, Max]	1.00<br>[0.00, 2.00]	-0.83<br>[-2.88, 0.50]	1.00<br>[0.00, 2.00]	-1.29<br>[-2.56, 0.00]	1.00<br>[0.00, 3.00]	-1.00<br>[-3.00, 0.67]
Day 9.5	N	77	77	70	70	80	80
	Mean±SD	0.83±0.51	-1.03±0.69	0.60±0.54	-1.29±0.60	0.86±0.58	-1.03±0.66
	Median<br>[Min, Max]	1.00<br>[0.00, 2.00]	-0.82<br>[-2.88, 0.50]	1.00<br>[0.00, 2.00]	-1.33<br>[-2.56, 0.00]	1.00<br>[0.00, 3.00]	-0.95<br>[-3.00, 0.67]
Day 10	N	77	77	70	70	80	80
	Mean±SD	0.81±0.51	-1.05±0.67	0.57±0.53	-1.33±0.59	0.77±0.59	-1.13±0.66
	Median<br>[Min, Max]	1.00<br>[0.00, 2.20]	-1.00<br>[-2.88, 0.50]	1.00<br>[0.00, 1.75]	-1.33<br>[-2.60, -0.25]	1.00<br>[0.00, 2.00]	-1.00<br>[-3.00, 0.00]

Category		The placebo group N=77		The low dose group N=70		The high dose group N=80	
		Values (Day#)	Change (Day# - baseline)	Values (Day#)		Values (Day#)	Change (Day# - baseline)
Day 10.5	N	77	77	70	70	80	80
	Mean±SD	0.80±0.52	-1.05±0.67	0.53±0.56	-1.36±0.63	0.80±0.56	-1.09±0.68
	Median [Min, Max]	1.00 [0.00, 2.00]	-1.00 [-2.88, 0.25]	0.50 [0.00, 2.17]	-1.41 [-2.60, 0.17]	1.00 [0.00, 2.00]	-1.00 [-3.00, 0.00]
Day 11	N	77	77	70	70	80	80
	Mean±SD	0.76±0.51	-1.09±0.67	0.58±0.54	-1.31±0.61	0.72±0.57	-1.18±0.68
	Median [Min, Max]	1.00 [0.00, 2.00]	-1.00 [-2.88, 0.25]	1.00 [0.00, 1.71]	-1.33 [-2.60, -0.29]	1.00 [0.00, 2.00]	-1.14 [-3.00, 0.00]
Day 11.5	N	77	77	70	70	80	80
	Mean±SD	0.75±0.56	-1.11±0.70	0.58±0.56	-1.31±0.64	0.75±0.59	-1.15±0.70
	Median [Min, Max]	1.00 [0.00, 2.00]	-1.00 [-2.88, 0.38]	1.00 [0.00, 2.00]	-1.33 [-2.60, 0.25]	1.00 [0.00, 3.00]	-1.07 [-3.00, 1.00]
Day 12	N	77	77	70	70	80	80
	Mean±SD	0.70±0.55	-1.16±0.70	0.54±0.54	-1.35±0.62	0.70±0.56	-1.20±0.67
	Median [Min, Max]	1.00 [0.00, 2.00]	-1.00 [-2.88, 0.38]	1.00 [0.00, 1.88]	-1.37 [-2.60, -0.13]	1.00 [0.00, 2.00]	-1.24 [-3.00, 0.00]
Day 12.5	N	77	77	70	70	80	80
	Mean±SD	0.70±0.56	-1.16±0.68	0.60±0.63	-1.29±0.68	0.74±0.62	-1.15±0.75
	Median [Min, Max]	1.00 [0.00, 2.00]	-1.00 [-2.88, 0.25]	1.00 [0.00, 2.75]	-1.33 [-2.60, 0.75]	1.00 [0.00, 3.00]	-1.07 [-3.00, 1.00]
Day 13	N	77	77	70	70	80	80
	Mean±SD	0.67±0.57	-1.19±0.66	0.54±0.55	-1.35±0.64	0.66±0.61	-1.23±0.71
	Median [Min, Max]	1.00 [0.00, 2.00]	-1.14 [-2.88, 0.25]	1.00 [0.00, 2.20]	-1.37 [-2.60, 0.20]	1.00 [0.00, 2.00]	-1.33 [-3.00, 0.14]

Category		The placebo group N=77		The low dose group N=70		The high dose group N=80	
		Values (Day#)	Change (Day# - baseline)	Values (Day#)		Values (Day#)	Change (Day# - baseline)
Day 13.5	N	77	77	70	70	80	80
	Mean±SD	0.66±0.61	-1.20±0.71	0.56±0.58	-1.33±0.68	0.70±0.59	-1.20±0.70
	Median [Min, Max]	1.00 [0.00, 2.20]	-1.20 [-2.88, 0.50]	1.00 [0.00, 2.00]	-1.33 [-2.67, 0.25]	1.00 [0.00, 2.44]	-1.19 [-3.00, 0.57]
Day 14	N	77	77	70	70	80	80
	Mean±SD	0.65±0.61	-1.20±0.67	0.53±0.54	-1.36±0.62	0.65±0.58	-1.25±0.67
	Median [Min, Max]	1.00 [0.00, 2.33]	-1.25 [-2.88, 0.52]	0.50 [0.00, 1.80]	-1.41 [-2.60, 0.00]	1.00 [0.00, 2.00]	-1.33 [-3.00, 0.00]
Day 14.5	N	77	77	70	70	80	80
	Mean±SD	0.67±0.63	-1.19±0.69	0.50±0.54	-1.39±0.65	0.70±0.64	-1.19±0.72
	Median [Min, Max]	1.00 [0.00, 2.33]	-1.25 [-2.88, 0.52]	0.00 [0.00, 1.60]	-1.46 [-2.67, -0.15]	1.00 [0.00, 3.00]	-1.24 [-3.00, 1.00]

Table 23. Secondary endpoint 4) ANCOVA results for the change in total symptom score (ITT)

Day #	The placebo group vs The low dose group			The placebo group vs The high dose group		
	LS Mean [95% CI]		p-value	LS Mean [95% CI]		p-value
	The placebo	The low dose		The placebo	The high dose	
Day 2	9.7887 [8.9324, 10.6451]	9.4112 [8.5592, 10.2632]	0.5390	9.6942 [8.8088, 10.5797]	9.8642 [8.9696, 10.7589]	0.7906
Day 2.5	7.3553 [6.5691, 8.1415]	8.0725 [7.2903, 8.8548]	0.2045	7.3188 [6.4703, 8.1673]	8.5287 [7.6714, 9.3860]	0.0497
Day 3	6.1623 [5.3992, 6.9255]	6.6979 [5.9386, 7.4571]	0.3286	6.1241 [5.4267, 6.8216]	6.6962 [5.9915, 7.4009]	0.2573
Day 3.5	5.1119 [4.3426, 5.8813]	5.7377 [4.9722, 6.5031]	0.2577	5.0986 [4.4411, 5.7560]	6.0452 [5.3809, 6.7095]	0.0476
Day 4	4.0134 [3.3176, 4.7092]	5.1079 [4.4156, 5.8002]	0.0293	4.0196 [3.4796, 4.5597]	4.9487 [4.4030, 5.4944]	0.0182
Day 4.5	3.5464 [2.9039, 4.1889]	4.2470 [3.6078, 4.8862]	0.1297	3.5546 [2.9941, 4.1152]	4.5484 [3.9820, 5.1148]	0.0150
Day 5	3.0817 [2.4830, 3.6804]	3.7273 [3.1316, 4.3229]	0.1339	3.0810 [2.5531, 3.6089]	3.8131 [3.2797, 4.3465]	0.0563
Day 5.5	2.7718 [2.1987, 3.3449]	3.0542 [2.4840, 3.6244]	0.4924	2.7716 [2.2365, 3.3067]	3.4936 [2.9529, 4.0343]	0.0632
Day 6	2.3617 [1.7651, 2.9584]	2.8944 [2.3008, 3.4881]	0.2142	2.3698 [1.8814, 2.8582]	2.8725 [2.3790, 3.3660]	0.1557
Day 6.5	2.6534 [2.0606, 3.2462]	2.9795 [2.3897, 3.5693]	0.4435	2.6831 [2.1862, 3.1800]	3.2297 [2.7277, 3.7318]	0.1293
Day 7	2.6318 [2.1405, 3.1232]	2.5362 [2.0473, 3.0250]	0.7861	2.6606 [2.2447, 3.0764]	2.5965 [2.1763, 3.0167]	0.8312
Day 7.5	2.4310 [1.9307, 2.9313]	2.3208 [1.8230, 2.8186]	0.7588	2.4460 [2.0152, 2.8768]	2.5656 [2.1303, 3.0009]	0.7011
Day 8	2.1132 [1.6433, 2.5831]	2.2617 [1.7942, 2.7292]	0.6597	2.1380 [1.7490, 2.5271]	2.1195 [1.7264, 2.5126]	0.9474
Day 8.5	1.9720 [1.5010, 2.4430]	2.0782 [1.6096, 2.5468]	0.7534	2.0011 [1.6184, 2.3838]	2.1447 [1.7580, 2.5313]	0.6040
Day 9	2.0601 [1.5842, 2.5360]	2.0516 [1.5782, 2.5251]	0.9802	2.0856 [1.6952, 2.4760]	1.9126 [1.5181, 2.3071]	0.5402
Day 9.5	1.9654 [1.4924, 2.4383]	2.0141 [1.5435, 2.4846]	0.8859	1.9867 [1.6151, 2.3583]	1.9511 [1.5756, 2.3266]	0.8945

Day #	The placebo group vs The low dose group			The placebo group vs The high dose group		
	LS Mean [95% CI]		p-value	LS Mean [95% CI]		p-value
	The placebo	The low dose		The placebo	The high dose	
Day 10	1.8460 [1.3878, 2.3042]	1.7989 [1.3431, 2.2548]	0.8862	1.8689 [1.5114, 2.2264]	1.8109 [1.4497, 2.1722]	0.8227
Day 10.5	1.8744 [1.4116, 2.3372]	1.7809 [1.3204, 2.2413]	0.7782	1.9024 [1.5337, 2.2711]	1.7976 [1.4251, 2.1701]	0.6943
Day 11	1.6984 [1.2363, 2.1605]	1.8541 [1.3943, 2.3138]	0.6387	1.7216 [1.3613, 2.0819]	1.5863 [1.2222, 1.9503]	0.6036
Day 11.5	1.6223 [1.1743, 2.0704]	1.7678 [1.3220, 2.2136]	0.6510	1.6504 [1.3152, 1.9856]	1.6173 [1.2786, 1.9560]	0.8914
Day 12	1.4426 [1.0101, 1.8751]	1.7538 [1.3235, 2.1841]	0.3165	1.4783 [1.1513, 1.8054]	1.5429 [1.2124, 1.8734]	0.7848
Day 12.5	1.5330 [1.0965, 1.9694]	1.7148 [1.2806, 2.1490]	0.5614	1.5564 [1.2180, 1.8948]	1.5049 [1.1630, 1.8468]	0.8331
Day 13	1.4946 [1.0577, 1.9314]	1.5407 [1.1061, 1.9753]	0.8829	1.5155 [1.1475, 1.8834]	1.4634 [1.0916, 1.8351]	0.8448
Day 13.5	1.3823 [0.9407, 1.8239]	1.5307 [1.0913, 1.9700]	0.6396	1.4054 [0.9586, 1.8522]	1.6070 [1.1556, 2.0584]	0.5327
Day 14	1.2703 [0.8326, 1.7081]	1.5203 [1.0847, 1.9558]	0.4265	1.2929 [0.9127, 1.6730]	1.3989 [1.0148, 1.7830]	0.6996
Day 14.5	1.3886 [0.9428, 1.8344]	1.4840 [1.0404, 1.9275]	0.7657	1.4131 [1.0348, 1.7913]	1.4221 [1.0399, 1.8043]	0.9737

Table 24. Secondary endpoint 4) ANCOVA results for the change in total symptom score (PPS)

Day #	The placebo group vs The low dose group			The placebo group vs The high dose group		
	LS Mean [95% CI]		p-value	LS Mean [95% CI]		p-value
	The placebo	The low dose		The placebo	The high dose	
Day 2	9.8599 [9.0002, 10.7195]	8.9684 [8.0663, 9.8706]	0.1622	9.7664 [8.9156, 10.6172]	9.5498 [8.7152, 10.3844]	0.7207
Day 2.5	7.2968 [6.5132, 8.0805]	7.4021 [6.5797, 8.2245]	0.8559	7.2195 [6.3419, 8.0971]	8.3637 [7.5028, 9.2246]	0.0686
Day 3	6.0613 [5.3206, 6.8021]	5.8611 [5.0837, 6.6385]	0.7148	6.0091 [5.2651, 6.7530]	6.3163 [5.5865, 7.0460]	0.5621
Day 3.5	5.0786 [4.3276, 5.8297]	4.8992 [4.1111, 5.6874]	0.7467	5.0732 [4.3359, 5.8105]	5.8795 [5.1562, 6.6028]	0.1260

Day #	The placebo group vs The low dose group			The placebo group vs The high dose group		
	LS Mean [95% CI]		p-value	LS Mean [95% CI]		p-value
	The placebo	The low dose		The placebo	The high dose	
Day 4	3.9337 [3.2657, 4.6017]	4.2158 [3.5147, 4.9168]	0.5682	3.9585 [3.3472, 4.5698]	4.7024 [4.1028, 5.3021]	0.0889
Day 4.5	3.3413 [2.7360, 3.9466]	3.4246 [2.7894, 4.0598]	0.8523	3.3565 [2.7449, 3.9681]	4.3569 [3.7569, 4.9568]	0.0227
Day 5	2.9126 [2.3297, 3.4954]	3.0390 [2.4273, 3.6507]	0.7693	2.9077 [2.3231, 3.4923]	3.7264 [3.1529, 4.2999]	0.0506
Day 5.5	2.5937 [2.0473, 3.1401]	2.4041 [1.8307, 2.9775]	0.6389	2.6040 [2.0097, 3.1983]	3.3686 [2.7856, 3.9516]	0.0723
Day 6	2.1376 [1.5888, 2.6863]	2.3344 [1.7585, 2.9103]	0.6277	2.1439 [1.6044, 2.6834]	2.8615 [2.3323, 3.3907]	0.0632
Day 6.5	2.4802 [1.9471, 3.0134]	2.1146 [1.5551, 2.6741]	0.3544	2.5251 [1.9913, 3.0588]	3.0446 [2.5211, 3.5682]	0.1729
Day 7	2.5459 [2.0660, 3.0259]	1.9137 [1.4101, 2.4174]	0.0764	2.5887 [2.1247, 3.0528]	2.5709 [2.1156, 3.0261]	0.9569
Day 7.5	2.3141 [1.8140, 2.8141]	1.8117 [1.2869, 2.3364]	0.1756	2.3503 [1.8743, 2.8263]	2.4503 [1.9834, 2.9173]	0.7679
Day 8	2.1501 [1.6709, 2.6294]	1.7492 [1.2462, 2.2521]	0.2589	2.1877 [1.7400, 2.6355]	2.0568 [1.6176, 2.4961]	0.6814
Day 8.5	1.9955 [1.5239, 2.4670]	1.6050 [1.1101, 2.0998]	0.2637	2.0478 [1.6068, 2.4887]	2.1165 [1.6840, 2.5491]	0.8266
Day 9	2.1789 [1.6823, 2.6756]	1.5603 [1.0391, 2.0815]	0.0937	2.2225 [1.7652, 2.6798]	1.8733 [1.4247, 2.3220]	0.2845
Day 9.5	2.0453 [1.5699, 2.5208]	1.4787 [0.9798, 1.9777]	0.1086	2.0776 [1.6441, 2.5110]	1.9503 [1.5251, 2.3756]	0.6803
Day 10	1.8924 [1.4310, 2.3538]	1.3326 [0.8484, 1.8168]	0.1024	1.9403 [1.5285, 2.3522]	1.7699 [1.3659, 2.1740]	0.5615
Day 10.5	1.9257 [1.4507, 2.4006]	1.3103 [0.8119, 1.8088]	0.0814	1.9704 [1.5396, 2.4011]	1.7910 [1.3684, 2.2136]	0.5588
Day 11	1.7823 [1.3116, 2.2529]	1.3823 [0.8884, 1.8763]	0.2515	1.8213 [1.3988, 2.2438]	1.5970 [1.1825, 2.0114]	0.4562
Day 11.5	1.6482 [1.1956, 2.1007]	1.3727 [0.8978, 1.8476]	0.4109	1.6964 [1.3125, 2.0803]	1.6047 [1.2282, 1.9813]	0.7375

Day #	The placebo group vs The low dose group			The placebo group vs The high dose group		
	LS Mean [95% CI]		p-value	LS Mean [95% CI]		p-value
	The placebo	The low dose		The placebo	The high dose	
Day 12	1.4294 [0.9779, 1.8810]	1.4419 [0.9680, 1.9158]	0.9701	1.5020 [1.1305, 1.8735]	1.5168 [1.1523, 1.8812]	0.9556
Day 12.5	1.5821 [1.1237, 2.0404]	1.3740 [0.8930, 1.8551]	0.5395	1.6344 [1.2495, 2.0193]	1.4644 [1.0868, 1.8419]	0.5352
Day 13	1.5413 [1.0965, 1.9861]	1.2474 [0.7806, 1.7142]	0.3722	1.5936 [1.1668, 2.0203]	1.4287 [1.0101, 1.8473]	0.5876
Day 13.5	1.5125 [1.0623, 1.9627]	1.2220 [0.7495, 1.6944]	0.3833	1.5522 [1.0234, 2.0810]	1.5935 [1.0748, 2.1122]	0.9127
Day 14	1.4100 [0.9664, 1.8536]	1.1347 [0.6692, 1.6003]	0.4020	1.4577 [1.0115, 1.9039]	1.4095 [0.9717, 1.8472]	0.8793
Day 14.5	1.4817 [1.0201, 1.9434]	1.1415 [0.6571, 1.6260]	0.3197	1.5298 [1.0969, 1.9627]	1.4151 [0.9904, 1.8398]	0.7098

Table 25. Secondary endpoint 4) ANCOVA results for the change in average symptom score (ITT)

Day #	The placebo group vs The low dose group			The placebo group vs The high dose group		
	LS Mean [95% CI]		p-value	LS Mean [95% CI]		p-value
	The placebo	The low dose		The placebo	The high dose	
Day 2	1.5181 [1.4499, 1.5863]	1.4657 [1.3978, 1.5335]	0.2844	1.5195 [1.4500, 1.5891]	1.4929 [1.4227, 1.5632]	0.5964
Day 2.5	1.3568 [1.2896, 1.4239]	1.3729 [1.3060, 1.4397]	0.7381	1.3614 [1.2880, 1.4349]	1.4167 [1.3425, 1.4909]	0.2989
Day 3	1.3046 [1.2344, 1.3749]	1.3094 [1.2395, 1.3793]	0.9251	1.3046 [1.2363, 1.3730]	1.2938 [1.2248, 1.3629]	0.8267
Day 3.5	1.2320 [1.1510, 1.3130]	1.2244 [1.1438, 1.3051]	0.8965	1.2335 [1.1595, 1.3076]	1.2454 [1.1705, 1.3202]	0.8248
Day 4	1.0995 [1.0137, 1.1854]	1.2195 [1.1341, 1.3049]	0.0523	1.1008 [1.0285, 1.1732]	1.2039 [1.1308, 1.2770]	0.0500
Day 4.5	1.0434 [0.9527, 1.1340]	1.1266 [1.0363, 1.2168]	0.2015	1.0441 [0.9667, 1.1215]	1.1941 [1.1159, 1.2723]	0.0079
Day 5	1.0144 [0.9169, 1.1119]	1.0063 [0.9093, 1.1033]	0.9075	1.0132 [0.9289, 1.0975]	1.1329 [1.0477, 1.2181]	0.0508
Day 5.5	1.0171 [0.9140,	0.9711 [0.8685,	0.5338	1.0147 [0.9238,	1.0789 [0.9870,	0.3294

Day #	The placebo group vs The low dose group			The placebo group vs The high dose group		
	LS Mean [95% CI]		p-value	LS Mean [95% CI]		p-value
	The placebo	The low dose		The placebo	The high dose	
	1.1201]	1.0736]		1.1057]	1.1708]	
Day 6	0.9138 [0.8069, 1.0206]	0.9078 [0.8014, 1.0141]	0.9379	0.9088 [0.8002, 1.0175]	0.9434 [0.8336, 1.0532]	0.6599
Day 6.5	0.9149 [0.8124, 1.0174]	0.8962 [0.7942, 0.9982]	0.7992	0.9175 [0.8237, 1.0114]	1.0571 [0.9623, 1.1519]	0.0408
Day 7	0.9564 [0.8592, 1.0536]	0.8747 [0.7780, 0.9714]	0.2419	0.9588 [0.8690, 1.0487]	0.9588 [0.8681, 1.0496]	1.0000
Day 7.5	0.9051 [0.8010, 1.0092]	0.7731 [0.6695, 0.8767]	0.0783	0.9091 [0.8165, 1.0016]	0.9610 [0.8675, 1.0545]	0.4383
Day 8	0.8329 [0.7220, 0.9438]	0.7867 [0.6764, 0.8971]	0.5614	0.8369 [0.7332, 0.9406]	0.8840 [0.7792, 0.9888]	0.5304
Day 8.5	0.7879 [0.6782, 0.8976]	0.7533 [0.6442, 0.8625]	0.6603	0.7924 [0.6851, 0.8997]	0.9378 [0.8294, 1.0462]	0.0620
Day 9	0.8222 [0.7088, 0.9356]	0.7263 [0.6135, 0.8392]	0.2392	0.8285 [0.7168, 0.9402]	0.8338 [0.7210, 0.9467]	0.9477
Day 9.5	0.8034 [0.6940, 0.9128]	0.7037 [0.5949, 0.8126]	0.2048	0.8082 [0.7000, 0.9164]	0.8835 [0.7742, 0.9928]	0.3360
Day 10	0.7937 [0.6832, 0.9042]	0.6572 [0.5472, 0.7672]	0.0861	0.8009 [0.6939, 0.9079]	0.7816 [0.6735, 0.8897]	0.8031
Day 10.5	0.7856 [0.6743, 0.8969]	0.6427 [0.5319, 0.7534]	0.0745	0.7917 [0.6856, 0.8978]	0.8052 [0.6980, 0.9124]	0.8605
Day 11	0.7291 [0.6177, 0.8405]	0.6836 [0.5728, 0.7945]	0.5694	0.7331 [0.6268, 0.8394]	0.7409 [0.6335, 0.8483]	0.9189
Day 11.5	0.7438 [0.6272, 0.8604]	0.6807 [0.5647, 0.7967]	0.4504	0.7482 [0.6339, 0.8625]	0.7568 [0.6413, 0.8723]	0.9178
Day 12	0.6944 [0.5800, 0.8087]	0.6500 [0.5362, 0.7638]	0.5885	0.6972 [0.5870, 0.8074]	0.7324 [0.6211, 0.8437]	0.6585
Day 12.5	0.6717 [0.5509, 0.7925]	0.6946 [0.5744, 0.8147]	0.7918	0.6773 [0.5625, 0.7921]	0.7588 [0.6428, 0.8748]	0.3269
Day 13	0.6610 [0.5451, 0.7769]	0.6238 [0.5085, 0.7391]	0.6546	0.6641 [0.5497, 0.7784]	0.6891 [0.5736, 0.8046]	0.7621
Day 13.5	0.6008 [0.4812,	0.6223 [0.5033,	0.8015	0.6071 [0.4898,	0.7037 [0.5852,	0.2554

Day #	The placebo group vs The low dose group			The placebo group vs The high dose group		
	LS Mean [95% CI]		p-value	LS Mean [95% CI]		p-value
	The placebo	The low dose		The placebo	The high dose	
	0.7204]	0.7413]		0.7244]	0.8222]	
Day 14	0.5934 [0.4760, 0.7108]	0.6194 [0.5026, 0.7362]	0.7577	0.5982 [0.4822, 0.7141]	0.6423 [0.5252, 0.7594]	0.5984
Day 14.5	0.6323 [0.5117, 0.7529]	0.6113 [0.4913, 0.7313]	0.8078	0.6402 [0.5171, 0.7632]	0.6866 [0.5622, 0.8109]	0.6020

Table 26. Secondary endpoint 4) ANCOVA results for the change in average symptom score (PPS)

Day #	The placebo group vs The low dose group			The placebo group vs The high dose group		
	LS Mean [95% CI]		p-value	LS Mean [95% CI]		p-value
	The placebo	The low dose		The placebo	The high dose	
Day 2	1.4805 [1.4071, 1.5538]	1.4435 [1.3665, 1.5204]	0.4932	1.4806 [1.4071, 1.5542]	1.4713 [1.3992, 1.5434]	0.8586
Day 2.5	1.3249 [1.2522, 1.3976]	1.3061 [1.2299, 1.3824]	0.7265	1.3269 [1.2466, 1.4071]	1.4072 [1.3285, 1.4860]	0.1606
Day 3	1.2896 [1.2123, 1.3669]	1.2450 [1.1639, 1.3261]	0.4334	1.2884 [1.2111, 1.3656]	1.2844 [1.2087, 1.3602]	0.9430
Day 3.5	1.2196 [1.1270, 1.3121]	1.1667 [1.0696, 1.2638]	0.4382	1.2192 [1.1346, 1.3038]	1.2546 [1.1716, 1.3377]	0.5566
Day 4	1.0862 [0.9867, 1.1857]	1.1599 [1.0555, 1.2643]	0.3151	1.0860 [1.0032, 1.1687]	1.2076 [1.1265, 1.2888]	0.0401
Day 4.5	1.0075 [0.8982, 1.1168]	1.0654 [0.9507, 1.1800]	0.4722	1.0082 [0.9199, 1.0965]	1.1727 [1.0862, 1.2593]	0.0096
Day 5	0.9770 [0.8605, 1.0936]	0.9646 [0.8423, 1.0868]	0.8843	0.9768 [0.8775, 1.0760]	1.1344 [1.0370, 1.2318]	0.0268
Day 5.5	0.9865 [0.8658, 1.1073]	0.9090 [0.7823, 1.0357]	0.3836	0.9852 [0.8796, 1.0908]	1.0565 [0.9529, 1.1601]	0.3433
Day 6	0.8694 [0.7446, 0.9942]	0.8384 [0.7074, 0.9693]	0.7356	0.8641 [0.7418, 0.9864]	0.9288 [0.8088, 1.0487]	0.4582
Day 6.5	0.8758 [0.7667, 0.9848]	0.7734 [0.6590, 0.8878]	0.2036	0.8787 [0.7760, 0.9813]	1.0374 [0.9367, 1.1381]	0.0310
Day 7	0.9418 [0.8357, 1.0479]	0.7601 [0.6489, 0.8714]	0.0212	0.9450 [0.8427, 1.0473]	0.9268 [0.8264, 1.0272]	0.8027
Day 7.5	0.8759	0.6582	0.0096	0.8800	0.9435	0.3884

Day #	The placebo group vs The low dose group			The placebo group vs The high dose group		
	LS Mean [95% CI]		p-value	LS Mean [95% CI]		p-value
	The placebo	The low dose		The placebo	The high dose	
	[0.7630, 0.9889]	[0.5397, 0.7767]		[0.7765, 0.9834]	[0.8420, 1.0450]	
Day 8	0.8368 [0.7117, 0.9619]	0.7045 [0.5733, 0.8357]	0.1522	0.8422 [0.7219, 0.9625]	0.8504 [0.7324, 0.9684]	0.9234
Day 8.5	0.7891 [0.6692, 0.9091]	0.6798 [0.5540, 0.8057]	0.2170	0.7928 [0.6683, 0.9174]	0.9184 [0.7962, 1.0405]	0.1580
Day 9	0.8337 [0.7119, 0.9556]	0.6229 [0.4951, 0.7507]	0.0199	0.8397 [0.7144, 0.9651]	0.8017 [0.6787, 0.9246]	0.6694
Day 9.5	0.8255 [0.7059, 0.9451]	0.6036 [0.4781, 0.7291]	0.0127	0.8279 [0.7035, 0.9523]	0.8633 [0.7413, 0.9854]	0.6890
Day 10	0.8047 [0.6867, 0.9227]	0.5686 [0.4448, 0.6924]	0.0073	0.8111 [0.6871, 0.9351]	0.7674 [0.6458, 0.8891]	0.6211
Day 10.5	0.8013 [0.6795, 0.9230]	0.5341 [0.4064, 0.6618]	0.0033	0.8055 [0.6829, 0.9282]	0.8013 [0.6810, 0.9217]	0.9618
Day 11	0.7617 [0.6425, 0.8809]	0.5863 [0.4613, 0.7113]	0.0471	0.7642 [0.6407, 0.8877]	0.7193 [0.5981, 0.8405]	0.6097
Day 11.5	0.7429 [0.6153, 0.8705]	0.5837 [0.4499, 0.7176]	0.0917	0.7461 [0.6157, 0.8765]	0.7461 [0.6182, 0.8741]	0.9999
Day 12	0.6981 [0.5741, 0.8222]	0.5431 [0.4130, 0.6733]	0.0911	0.7010 [0.5742, 0.8278]	0.6989 [0.5745, 0.8233]	0.9820
Day 12.5	0.6958 [0.5611, 0.8304]	0.5999 [0.4586, 0.7412]	0.3342	0.6996 [0.5650, 0.8343]	0.7418 [0.6097, 0.8739]	0.6599
Day 13	0.6697 [0.5422, 0.7972]	0.5409 [0.4072, 0.6747]	0.1716	0.6735 [0.5392, 0.8078]	0.6599 [0.5281, 0.7916]	0.8865
Day 13.5	0.6576 [0.5252, 0.7901]	0.5630 [0.4241, 0.7020]	0.3325	0.6653 [0.5293, 0.8014]	0.6904 [0.5569, 0.8238]	0.7959
Day 14	0.6549 [0.5241, 0.7856]	0.5242 [0.3870, 0.6614]	0.1759	0.6568 [0.5229, 0.7908]	0.6421 [0.5107, 0.7736]	0.8775
Day 14.5	0.6690 [0.5359, 0.8020]	0.4979 [0.3583, 0.6376]	0.0824	0.6752 [0.5321, 0.8183]	0.6974 [0.5570, 0.8378]	0.8275

5) Time (days) taken for the total score of all COVID-19 symptoms to decrease by 25% or more and by 50% or more by Day 14

For the placebo, Low dose, and The high dose group, descriptive statistics for the time taken time (days)

taken for the total score of all COVID-19 symptoms to decrease by 25% or more and by 50% or more by Day 14 are presented. The time taken for a decrease of more than 25% is shown in Tables 27–28, and for a decrease of more than 50% in Tables 29–30. Participants whose total symptom score did not decrease by at least 25% or 50% by the evening of Day 14 (= Day 14.5) were censored to have the maximum possible value as 13.00 days (maximum value of 13 days = 14.5 days - (first day of dosing + 0.5 day))

Table 27. Secondary endpoint 5) descriptive statistics - time (days) taken for the total symptom score to decrease by 25% or more (ITT)

Total score		The placebo group N=98	The low dose group N=99	The high dose group N=96
Time (days) taken for the total score of all COVID-19 symptoms to decrease by 25% or more	N	98	99	96
	Mean	2.06	2.56	2.20
	SD	2.54	3.38	2.56
	Median	1.00	1.00	1.25
	Min	0.50	0.50	0.50
	Max	13.00	13.00	13.00

Table 28. Secondary endpoint 5) descriptive statistics - time (days) taken for the total symptom score to decrease by 25% or more (PPS)

Total score		The placebo group N=77	The low dose group N=70	The high dose group N=80
Time (days) taken for the total score of all COVID-19 symptoms to decrease by 25% or more	N	77	70	80
	Mean	1.68	1.69	1.83
	SD	1.95	2.57	2.27
	Median	1.00	1.00	1.00
	Min	0.50	0.50	0.50
	Max	10.50	13.00	13.00

Table 29. Secondary endpoint 5) descriptive statistics - time (days) taken for the total symptom score to decrease by 50% or more (ITT)

Total score		The placebo group N=98	The low dose group N=99	The high dose group N=96
Time (days) taken for the total score of all COVID-19 symptoms to decrease by 50% or more	N	98	99	96
	Mean	3.39	3.93	3.46
	SD	3.56	4.06	3.28
	Median	2.00	2.00	2.50
	Min	0.50	0.50	0.50
	Max	13.00	13.00	13.00

Table 30. Secondary endpoint 5) descriptive statistics - time (days) taken for the total symptom score to decrease by 50% or more (PPS)

Total score		The placebo group N=77	The low dose group N=70	The high dose group N=80
Time (days) taken for the total score of all COVID-19 symptoms to decrease by 50% or more	N	77	70	80
	Mean	3.06	3.04	3.03
	SD	3.34	3.59	3.14
	Median	1.50	1.50	2.00
	Min	0.50	0.50	0.50
	Max	13.00	13.00	13.00

For the ITT analysis, the ANCOVA results indicated that at a 5% significance level, there was no statistically significant difference between the placebo group, the low dose group, and the high dose group in the time taken for a 25% or more, or 50% or more reduction in total symptom score.
Similarly, in the PPS analysis, no statistically significant difference was observed between the placebo group and both, the low dose group, and the high dose group in the time taken for a 25% or more, or 50% or more reduction in total symptom score.

6) Presence and number of COVID-19 symptoms newly developed by Day 14, excluding baseline symptoms
The presence and number of new COVID-19 symptoms by Day 14 other than pre-assessed symptoms at baseline for the placebo, the low dose, and the high dose group are presented in Tables 31–32 and the number of new symptoms for each is presented in Tables 33–34. Symptoms that were present at baseline and symptoms that did not appear throughout the study were not assessed.
In ITT, the number of participants who developed new symptoms was 75 (198 cases) in the placebo group, 80 (238 cases) in the low dose group, and 88 (246 cases) in the high dose group.
In PPS, the number of participants who developed new symptoms was 55 (143 cases) in the placebo group, 52 (120 cases) in the low dose group, and 72 (178 cases) in the high dose group.
Descriptive statistics for the number of new symptoms by Day 14 per participant across all groups are presented in Tables 35–36, followed by an ANCOVA analysis with age and severity as covariates, the results of which are presented in Tables 37–38.

Table 31. Secondary endpoint 6) number of new symptoms by Day (ITT)

New symptoms presence	The placebo group N=98 case (%)		The low dose group N=99 case (%)		The high dose group N=96 case (%)	
	Number of newly reported symptoms	Cumulative number of newly reported symptoms	Number of newly reported symptoms	Cumulative number of newly reported symptoms	Number of newly reported symptoms	Cumulative number of newly reported symptoms
Day 2	123(62.1)	123(62.1)	143(60.1)	143(60.1)	153(62.2)	153(62.2)
Day 2.5	24(12.1)	147(74.2)	33(13.9)	176(73.9)	32(13.0)	185(75.2)
Day 3	11(5.6)	158(79.8)	16(6.7)	192(80.7)	17(6.9)	202(82.1)
Day 3.5	6(3.0)	164(82.8)	9(3.8)	201(84.5)	8(3.3)	210(85.4)
Day 4	2(1.0)	166(83.8)	8(3.4)	209(87.8)	1(0.4)	211(85.8)
Day 4.5	4(2.0)	170(85.9)	7(2.9)	216(90.8)	9(3.7)	220(89.4)
Day 5	5(2.5)	175(88.4)	6(2.5)	222(93.3)	6(2.4)	226(91.9)

New symptoms presence	The placebo group N=98 case (%)		The low dose group N=99 case (%)		The high dose group N=96 case (%)	
	Number of newly reported symptoms	Cumulative number of newly reported symptoms	Number of newly reported symptoms	Cumulative number of newly reported symptoms	Number of newly reported symptoms	Cumulative number of newly reported symptoms
Day 5.5	1(0.5)	176(88.9)	1(0.4)	223(93.7)	2(0.8)	228(92.7)
Day 6	4(2.0)	180(90.9)	4(1.7)	227(95.4)	4(1.6)	232(94.3)
Day 6.5	3(1.5)	183(92.4)	5(2.1)	232(97.5)	4(1.6)	236(95.9)
Day 7	3(1.5)	186(93.9)	0(0.0)	232(97.5)	2(0.8)	238(96.7)
Day 7.5	2(1.0)	188(94.9)	2(0.8)	234(98.3)	1(0.4)	239(97.2)
Day 8	0(0.0)	188(94.9)	0(0.0)	234(98.3)	0(0.0)	239(97.2)
Day 8.5	0(0.0)	188(94.9)	0(0.0)	234(98.3)	2(0.8)	241(98.0)
Day 9	0(0.0)	188(94.9)	1(0.4)	235(98.7)	0(0.0)	241(98.0)
Day 9.5	0(0.0)	188(94.9)	0(0.0)	235(98.7)	0(0.0)	241(98.0)
Day 10	0(0.0)	188(94.9)	1(0.4)	236(99.2)	0(0.0)	241(98.0)
Day 10.5	2(1.0)	190(96.0)	1(0.4)	237(99.6)	0(0.0)	241(98.0)
Day 11	3(1.5)	193(97.5)	0(0.0)	237(99.6)	1(0.4)	242(98.4)
Day 11.5	2(1.0)	195(98.5)	0(0.0)	237(99.6)	0(0.0)	242(98.4)
Day 12	0(0.0)	195(98.5)	1(0.4)	238(100.0)	0(0.0)	242(98.4)
Day 12.5	1(0.5)	196(99.0)	0(0.0)	238(100.0)	0(0.0)	242(98.4)
Day 13	0(0.0)	196(99.0)	0(0.0)	238(100.0)	1(0.4)	243(98.8)
Day 13.5	0(0.0)	196(99.0)	0(0.0)	238(100.0)	2(0.8)	245(99.6)
Day 14	1(0.5)	197(99.5)	0(0.0)	238(100.0)	1(0.4)	246(100.0)
Day 14.5	1(0.5)	198(100.0)	0(0.0)	238(100.0)	0(0.0)	246(100.0)

Table 32. Secondary endpoint 6) number of new symptoms by Day (PPS)

New symptoms presence	The placebo group N=77 case (%)		The low dose group N=70 case (%)		The high dose group N=80 case (%)	
	Number of newly reported symptoms	Cumulative number of newly reported symptoms	Number of newly reported symptoms	Cumulative number of newly reported symptoms	Number of newly reported symptoms	Cumulative number of newly reported symptoms
Day 2	90(62.9)	90(62.9)	66(55.0)	66(55.0)	103(57.9)	103(57.9)
Day 2.5	19(13.3)	109(76.2)	21(17.5)	87(72.5)	29(16.3)	132(74.2)
Day 3	8(5.6)	117(81.8)	11(9.2)	98(81.7)	10(5.6)	142(79.8)
Day 3.5	4(2.8)	121(84.6)	4(3.3)	102(85.0)	7(3.9)	149(83.7)
Day 4	2(1.4)	123(86.0)	4(3.3)	106(88.3)	0(0.0)	149(83.7)
Day 4.5	3(2.1)	126(88.1)	5(4.2)	111(92.5)	8(4.5)	157(88.2)

New symptoms presence	The placebo group N=77 case (%)		The low dose group N=70 case (%)		The high dose group N=80 case (%)	
	Number of newly reported symptoms	Cumulative number of newly reported symptoms	Number of newly reported symptoms	Cumulative number of newly reported symptoms	Number of newly reported symptoms	Cumulative number of newly reported symptoms
Day 5	2(1.4)	128(89.5)	1(0.8)	112(93.3)	6(3.4)	163(91.6)
Day 5.5	0(0.0)	128(89.5)	1(0.8)	113(94.2)	1(0.6)	164(92.1)
Day 6	4(2.8)	132(92.3)	3(2.5)	116(96.7)	4(2.2)	168(94.4)
Day 6.5	1(0.7)	133(93.0)	1(0.8)	117(97.5)	2(1.1)	170(95.5)
Day 7	3(2.1)	136(95.1)	0(0.0)	117(97.5)	1(0.6)	171(96.1)
Day 7.5	0(0.0)	136(95.1)	1(0.8)	118(98.3)	1(0.6)	172(96.6)
Day 8	0(0.0)	136(95.1)	0(0.0)	118(98.3)	0(0.0)	172(96.6)
Day 8.5	0(0.0)	136(95.1)	0(0.0)	118(98.3)	2(1.1)	174(97.8)
Day 9	0(0.0)	136(95.1)	0(0.0)	118(98.3)	0(0.0)	174(97.8)
Day 9.5	0(0.0)	136(95.1)	0(0.0)	118(98.3)	0(0.0)	174(97.8)
Day 10	0(0.0)	136(95.1)	1(0.8)	119(99.2)	0(0.0)	174(97.8)
Day 10.5	1(0.7)	137(95.8)	0(0.0)	119(99.2)	0(0.0)	174(97.8)
Day 11	3(2.1)	140(97.9)	0(0.0)	119(99.2)	1(0.6)	175(98.3)
Day 11.5	1(0.7)	141(98.6)	0(0.0)	119(99.2)	0(0.0)	175(98.3)
Day 12	0(0.0)	141(98.6)	1(0.8)	120(100.0)	0(0.0)	175(98.3)
Day 12.5	0(0.0)	141(98.6)	0(0.0)	120(100.0)	0(0.0)	175(98.3)
Day 13	0(0.0)	141(98.6)	0(0.0)	120(100.0)	1(0.6)	176(98.9)
Day 13.5	0(0.0)	141(98.6)	0(0.0)	120(100.0)	1(0.6)	177(99.4)
Day 14	1(0.7)	142(99.3)	0(0.0)	120(100.0)	1(0.6)	178(100.0)
Day 14.5	1(0.7)	143(100.0)	0(0.0)	120(100.0)	0(0.0)	178(100.0)

Table 33. Secondary endpoint 6) number of new symptoms (ITT)

Newly reported symptoms after baseline	The placebo case (%)	The low dose case (%)	The high dose case (%)
Total*	198(100.0)	238(100.0)	246(100.0)
Fever	25(12.6)	28(11.8)	27(11.0)
Cough	7(3.5)	12(5.0)	13(5.3)
Sore throat	7(3.5)	5(2.1)	8(3.3)
Headache	20(10.1)	23(9.7)	25(10.2)
Muscle ache	8(4.0)	5(2.1)	10(4.1)
Chill	12(6.1)	11(4.6)	12(4.9)
Stuffy or runny nose	26(13.1)	28(11.8)	23(9.3)
Fatigue	19(9.6)	17(7.1)	21(8.5)
Difficulty of breathing	18(9.1)	15(6.3)	15(6.1)
Nausea	17(8.6)	32(13.4)	24(9.8)

Vomiting	5(2.5)	9(3.8)	7(2.8)
Diarrhea	34(17.2)	53(22.3)	61(24.8)

* Based on the sum of all days up to Day 14

Table 34. Secondary endpoint 6) number of new symptoms (PPS)

Newly reported symptoms after baseline	The placebo group (%)	The low dose group (%)	The high dose group (%)
Total*	143(100.0)	120(100.0)	178(100.0)
Fever	20(14.0)	14(11.7)	19(10.7)
Cough	5(3.5)	6(5.0)	7(3.9)
Sore throat	5(3.5)	1(0.8)	4(2.2)
Headache	11(7.7)	13(10.8)	18(10.1)
Muscle ache	6(4.2)	3(2.5)	8(4.5)
Chill	6(4.2)	6(5.0)	10(5.6)
Stuffy or runny nose	19(13.3)	12(10.0)	16(9.0)
Fatigue	14(9.8)	5(4.2)	14(7.9)
Difficulty of breathing	13(9.1)	8(6.7)	10(5.6)
Nausea	14(9.8)	15(12.5)	18(10.1)
Vomiting	5(3.5)	4(3.3)	6(3.4)
Diarrhea	25(17.5)	33(27.5)	48(27.0)

* Based on the sum of all days up to Day 14

Table 35. Secondary endpoint 6) descriptive statistics for the number of new symptoms (ITT)

Number of new symptoms		The placebo group N=98	The low dose group N=99	The high dose group N=96
Number of new symptoms per participant	N	98	99	96
	Mean	2.02	2.40	2.56
	SD	1.95	2.15	2.05
	Median	1.50	2.00	2.00
	Min	0.00	0.00	0.00
	Max	8.00	9.00	9.00

Table 36. Secondary endpoint 6) descriptive statistics for the number of new symptoms (PPS)

Number of new symptoms		The placebo group N=77	The low dose group N=70	The high dose group N=80
Number of new symptoms per participant	N	77	70	80
	Mean	1.86	1.71	2.23
	SD	1.95	1.72	1.80
	Median	1.00	1.00	2.00
	Min	0.00	0.00	0.00
	Max	8.00	7.00	9.00

Table 37. Secondary endpoint 6) ANCOVA results (ITT)

Category	The placebo group vs The low dose group			The placebo group vs The high dose group		
	LS Mean [95% CI]		p-value	LS Mean [95% CI]		p-value
	Placebo	Low dose		Placebo	High dose	
ITT	2.0248 [1.6338, 2.4158]	2.3997 [2.0106, 2.7887]	0.1821	2.0171 [1.6342, 2.4000]	2.5659 [2.1790, 2.9527]	0.0486

Table 38. Secondary endpoint 6) ANCOVA results (PPS)

Category	The placebo group vs The low dose group			The placebo group vs The high dose group		
	LS Mean [95% CI]		p-value	LS Mean [95% CI]		p-value
	The placebo	The low dose		The placebo	The high dose	
PPS	1.8780 [1.4664, 2.2896]	1.6914 [1.2596, 2.1231]	0.5378	1.8777 [1.4597, 2.2958]	2.2052 [1.7950, 2.6153]	0.2716

In ITT, new symptoms were reported until the Day 14 evening in the placebo group, while no new symptoms appeared after the day 12 morning in the low dose group. However, the ancova analysis did not show a statistically significant difference in the number of newly reported symptoms between the placebo group and the low dose group at the 5% significance level. Between The placebo group and the low dose group, the LSMEAN [95% confidence interval] of the number of newly reported COVID-19 symptoms after baseline was 2.02 [1.63, 2.42] cases for the placebo and 2.40 [2.01, 2.79] cases for the low dose group. New symptoms were reported until the Day 14 evening in the placebo group, while no new symptoms appeared after the Day 14 morning in the high dose group. Between The placebo group and the high dose group, the LSMEAN [95% confidence interval] was 2.02 [1.63, 2.40] cases for the former and 2.57 [2.18, 2.95] cases for the latter. The most frequently reported new COVID-19 symptoms after baseline were diarrhea (34 cases), stuffy or runny nose (26 cases), and fever (25 cases) in The placebo group; diarrhea (53 cases), nausea (32 cases), fever (28 cases), and stuffy or runny nose (28 cases) in the low dose group; and diarrhea (61 cases), fever (27 cases), and headache (25 cases) in the high dose group.

In PPS, new symptoms were reported until the Day 14 evening in the placebo group, while no new symptoms appeared after the Day 12 morning in the low dose group. However, the ANCOVA analysis did not show a statistically significant difference in the number of newly reported symptoms between the placebo group and the low dose group at the 5% significance level. Between the placebo group and the low dose group, the LSMEAN [95% confidence interval] of the number of newly reported COVID-19 symptoms after baseline was 1.88 [1.47, 2.29] cases for the placebo and 1.69 [1.26, 2.12] cases for the low dose group.

New symptoms continued to be reported in the placebo group until the evening of Day 14, whereas no new symptoms were observed in the high-dose group after the morning of Day 14. however, the ancova analysis did not show a statistically significant difference in the number of newly reported symptoms between the placebo group and the high dose group at the 5% significance level. between the placebo group and the high dose group, the LSMEAN [95% confidence interval] was 1.88 [1.46, 2.30] cases for the placebo and 2.21 [1.80, 2.62] cases for the high dose group.

The most frequently reported new COVID-19 symptoms after baseline were diarrhea (25 cases), fever (20 cases), and stuffy or runny nose (19 cases) in the placebo group; diarrhea (33 cases), nausea (15 cases), and fever (14 cases) in the low dose group; and diarrhea (48 cases), fever (19 cases), headache (18 cases) and nausea (18 cases) in the high dose group.

7) Changes in SARS-CoV-2 viral load on Day 2, Day 4, Day 6 and Day 8 compared to Day 0 (baseline, before administration of the investigational product)

Tables 39–40 present the descriptive statistics of the changes in SARS-CoV-2 viral load on Day 2, Day 4, Day 6 and Day 8 compared to Day 0 for the placebo group, the low dose, and the high dose group. ANCOVA analysis of viral load on each day was performed with pre-dose viral load, age and severity as covariates, and the results are presented in Tables 41–42.

Table 39. Secondary endpoint 7) descriptive statistics for viral load (ITT)

Category		The placebo group N=97*		The low dose group N=99		The high dose group N=96	
		Viral load (copy/μL) (Day#)	Changes (copy/μL) (Day# - Baseline)	Viral load (copy/μL) (Day#)	Changes (copy/μL) (Day# - Baseline)	Viral load (copy/μL) (Day#)	Changes (copy/μL) (Day# - Baseline)
Baseline	N	66	NA	68	NA	64	NA
	Mean±SD	255466.78±380003.16	NA	187929.63±359589.40	NA	254543.91±369854.73	NA
	Median [Min, Max]	87751.30 [0.00, 1416730.90]	NA	28551.95 [0.00, 1844137.80]	NA	61727.85 [0.00, 1950068.70]	NA
Day 2	N	66	66	67	67	64	64
	Mean±SD	209184.70±340232.40	-46282.08±413674.57	127748.23±215091.35	-62918.92±399629.87	119432.86±205559.19	-135111.05±407691.30
	Median [Min, Max]	32382.00 [0.00, 1466562.00]	-12041.45 [-1170274.70, 1369531.20]	9863.10 [0.00, 804875.80]	-260.30 [-1834274.70, 720880.40]	30073.25 [0.00, 1016710.20]	-8524.30 [-1906124.90, 852987.00]
Day 4	N	66	66	67	67	64	64
	Mean±SD	7082.25±15756.48	-248384.53±379357.51	7988.30±16908.54	-182678.85±362387.47	4060.35±7902.02	-250483.57±370959.21
	Median [Min, Max]	1187.25 [0.00, 99986.50]	-80133.00 [-1411962.40, 38966.60]	547.20[0.00, 88372.80]	-17899.90 [-1844076.00, 21236.30]	807.70[0.00, 51871.50]	-59792.90 [-1945687.10, 18485.90]
Day 6	N	66	66	67	67	64	64
	Mean±SD	875.72±1793.65	-254591.02±379687.89	612.55±1575.38	-190054.60±361584.99	488.66±1209.27	-254055.25±370017.17
	Median [Min, Max]	113.45 [0.00, 9744.40]	-86834.50 [-1416723.30, 6447.30]	67.50 [0.00, 9982.80]	-30394.70 [-1844115.00, 4468.70]	38.60[0.00, 6552.10]	-61632.00 [-1950011.00, 3364.10]
Day 8	N	66	66	67	67	64	64
	Mean±SD	31.84±70.83	-255434.90±380000.69	24.14±57.87	-190643.01±361569.89	13.46±29.71	-254530.45±369851.19
	Median [Min, Max]	2.50 [0.00, 340.00]	-87748.80 [-1416688.80, 5.50]	2.50[0.00, 339.20]	-30706.70 [-1844020.90, 0.00]	2.50[0.00, 179.10]	-61709.50 [-1950063.30, 0.00]

* Exclude patients with missing viral load data on Day 0 (major protocol deviation)

Table 40. Secondary endpoint 7) descriptive statistics for viral load (PPS)

Category		The placebo group N=77		The low dose group N=70		The high dose group N=80	
		Viral load (copy/μL) (Day#)	Changes (copy/μL) (Day# - Baseline)	Viral load (copy/μL) (Day#)	Changes (copy/μL) (Day# - Baseline)	Viral load (copy/μL) (Day#)	Changes (copy/μL) (Day# - Baseline)
Baseline	N	49	NA	48	NA	53	NA
	Mean±SD	254116.23±375927.57	NA	234127.93±415127.39	NA	286037.29±394243.88	NA
	Median [Min, Max]	89891.50 [2.50, 1416730.90]	NA	37667.70 [2.50, 1844137.80]	NA	73453.60[18.70, 1950068.70]	NA
Day 2	N	49	49	48	48	53	53
	Mean±SD	243691.53±377347.33	-10424.70±433403.82	101505.70±191804.50	-132622.23±427545.74	128115.68±215102.09	-157921.61±435491.05
	Median [Min, Max]	36153.90 [72.30, 1466562.00]	-12281.10 [-1170274.70, 1369531.20]	10820.90 [2.50, 804875.80]	-4494.40 [-1834274.70, 720880.40]	33397.80 [0.00, 1016710.20]	-4965.10 [-1906124.90, 852987.00]
Day 4	N	49	49	48	48	53	53
	Mean±SD	4642.97±8647.13	-249473.26±374727.68	6845.29±13517.93	-227282.64±416103.12	3927.29±8299.42	-282110.00±395380.78
	Median [Min, Max]	1550.50 [0.00, 49907.10]	-85101.40 [-1411962.40, 8005.50]	463.10 [0.00, 72330.10]	-17973.65 [-1844076.00, 21236.30]	803.30 [0.00, 51871.50]	-63664.70 [-1945687.10, 18485.90]
Day 6	N	49	49	48	48	53	53
	Mean±SD	769.42±1717.89	-253346.80±375678.84	449.95±1071.14	-233677.98±415050.06	471.13±1274.37	-285566.16±394404.88
	Median [Min, Max]	111.80 [0.00, 9744.40]	-88076.20 [-1416723.30, 0.00]	59.45 [0.00, 5465.90]	-36883.80 [-1844115.00, 240.80]	23.90 [0.00, 6552.10]	-67834.00 [-1950011.00, 3364.10]
Day 8	N	49	49	48	48	53	53
	Mean±SD	35.34±79.13	-254080.89±375921.77	27.04±65.10	-234100.89±415105.17	12.87±28.18	-286024.42±394239.18
	Median [Min, Max]	2.50 [0.00, 340.00]	-89889.00 [-1416688.80, -2.50]	2.50 [0.00, 339.20]	-37606.05 [-1844020.90, -2.50]	2.50 [0.00, 179.10]	-73401.30 [-1950063.30, -16.20]

Table 41. Secondary endpoint 7) viral load ANCOVA results (ITT)

Category	The placebo group vs The low dose group			The placebo group vs The high dose group		
	LS Mean [95% CI]		p-value	LS Mean [95% CI]		p-value
	The placebo	The low dose		The placebo	The high dose	
Day 2	204612.7098 [137739.4192, 271486.0004]	132251.9799 [65882.4268, 198621.5330]	0.1321	214196.3325 [147322.1807, 281070.4843]	114264.6087 [46343.5992, 182185.6182]	0.0410†
Day 4	6952.4609 [2977.2464, 10927.6753]	8116.1490 [4170.8788, 12061.4193]	0.6825	6863.7298 [3793.4780, 9933.9816]	4285.6927 [1167.3788, 7404.0066]	0.2481
Day 6	851.3469 [442.9413, 1259.7526]	636.5627 [231.2335, 1041.8919]	0.4628	857.9280 [480.5215, 1235.3346]	507.0117 [123.6972, 890.3262]	0.2012
Day 8	31.0691 [15.3874, 46.7508]	24.9021 [9.3385, 40.4656]	0.5828	31.8916 [18.4803, 45.3029]	13.4087 [-0.2126, 27.0299]	0.0591

* Unit: copy/μL
† Observed difference is statistically significant at the 5% level

Table 42. Secondary endpoint 7) viral load ANCOVA results (PPS)

Category	The placebo group vs The low dose group			The placebo group vs The high dose group		
	LS Mean [95% CI]		p-value	LS Mean [95% CI]		p-value
	The placebo	The low dose		The placebo	The high dose	
Day 2	243605.5148 [160956.3339, 326254.6958]	101593.5057 [18086.7575, 185100.2539]	0.0185†	247027.9904 [162014.0310, 332041.9499]	125031.0334 [43310.3119, 206751.7549]	0.0434†
Day 4	4679.1513 [1437.7266, 7920.5759]	6808.3539 [3533.2962, 10083.4116]	0.3615	4678.3489 [2236.6517, 7120.0462]	3894.5793 [1547.4676, 6241.6910]	0.6482
Day 6	769.0482 [364.1986, 1173.8979]	450.3278 [41.2775, 859.3782]	0.2746	741.8508 [313.9466, 1169.7550]	496.6210 [85.2928, 907.9492]	0.4158
Day 8	35.2943 [14.9439, 55.6448]	27.0871 [6.5254, 47.6487]	0.5747	34.9360 [18.1977, 51.6743]	13.2422 [-2.8477, 29.3321]	0.0676

* Unit: copy/μL
† Observed difference is statistically significant at the 5% level

In ITT, the ANCOVA analysis showed that on Day 2 (approximately 16 hours after the first dose), the LSMEAN [95% confidence interval] viral load on Day 2 adjusted on the baseline of the placebo and the low dose groups were 204612.71 [137739.42, 271486.00] and 132251.98 [65882.43, 198621.53], respectively, indicating that the viral load in the low dose group was approximately 64.6% of that in the placebo group. however, the p-value for the between-group difference on day 2 was 0.1321, and there was no statistically significant difference between groups at the 5% significance level on any other day. On Day 2 (approximately 16 hours after the first dose), the LSMEAN [95% confidence interval] viral load on Day 2 adjusted on the baseline of the placebo and the high dose groups were 214196.33 [147322.18, 281070.48] and 114264.61 [46343.60, 182185.62], respectively, indicating that the viral load in the high dose group was approximately 53.3% of that in the placebo group. The value for the

between-group difference on day 2 was 0.0410, which is statistically significant at the 5% significance level, while the other days did not show a statistically significant difference between groups at the 5% significance level.
in pps, the descriptive statistics for change in viral load from day 2 compared to pre-dosing showed a mean decrease of -10424.70 copy/μl for the placebo group and -132622.23 copy/μl for the low dose group, indicating approximately a 12.72-fold decrease. Furthermore, the ANCOVA analysis for the same day (Day 2) also showed statistically significant between-group differences at the 5% significance level (p-value=0.0185). The ANCOVA analysis showed a statistically significant difference between groups on Day 2 (approximately 16 hours after the first dose), with a significance level of 5%. The LSMEAN [95% confidence interval] for viral load on Day 2 was 243605.51 [160956.33, 326254.70] for the placebo group and 101593.51 [18086.76, 185100.25] for the low dose group, indicating that the viral load in the latter was approximately 41.7% of that in the former.
The descriptive statistics for change in viral load from Day 2 compared to pre-dosing showed a mean decrease of -10424.70 copy/μL for the placebo group and -157921.61 copy/μL for the high dose group, indicating approximately a 15.15-fold decrease. Furthermore, the ANCOVA analysis for the same day (Day 2) also showed statistically significant between-group differences at the 5% significance level (p-value=0.0434). The ANCOVA analysis showed a statistically significant difference between groups on Day 2 (approximately 16 hours after the first dose), with a significance level of 5%. The LSMEAN [95% confidence interval] for viral load on Day 2 was 247027.99 [162014.03, 332041.95] for the placebo group and 125031.03 [43310.31, 206751.75] for the high dose group, indicating that the viral load in the latter was approximately 50.6% of that in the former.

8) The dose and dosing frequency of Acetaminophen, Ibuprofen, and antidiarrheal from Day 1 to Day 28
For the placebo, the low dose, and the high dose group, from Day 1 to Day 28, the dosage and number of administrations for rescue medications including Acetaminophen, Ibuprofen, and antidiarrheals were detailed. Specifically, for Acetaminophen and Ibuprofen (for combination drugs, measure only Acetaminophen or Ibuprofen doses only), the number of participants administered (daily N) and dosages are presented in Tables 43–44. Descriptive statistics for the number of administrations of antidiarrheals are provided in Tables 45–46.
Additionally, an ANCOVA analysis, considering age and severity as covariates, was conducted on the total dosages of Acetaminophen and Ibuprofen administered during the period. Due to insufficient data, the number of administrations for antidiarrheals was not presented through ANCOVA analysis. The ANCOVA analysis results showed that there was no statistical significance between the placebo and the CP-COV03 groups on the total amounts of Acetaminophen and Ibuprofen used until Day 28, at the 5% significance level.

Table 43. Secondary endpoint 8) Acetaminophen, Ibuprofen dosages (mg) (ITT)

Acetaminophen, Ibuprofen		The placebo group N=98	The low dose group N=99	The high dose group N=96
Day 1	N	19	21	17
	Mean	580.26	647.62	641.18
	SD	134.00	141.84	36.38
	Median	650.00	650.00	650.00
	Min	200.00	350.00	500.00
	Max	650.00	1000.00	650.00
Day 2	N	9	22	8
	Mean	652.78	681.82	650.00

Acetaminophen, Ibuprofen		The placebo group N=98	The low dose group N=99	The high dose group N=96
	SD	168.84	102.99	0.00
	Median	650.00	650.00	650.00
	Min	325.00	650.00	650.00
	Max	1000.00	1000.00	650.00
Day 3	N	6	16	6
	Mean	595.83	693.75	650.00
	SD	132.68	119.55	0.00
	Median	650.00	650.00	650.00
	Min	325.00	650.00	650.00
	Max	650.00	1000.00	650.00
Day 4	N	5	3	4
	Mean	725.00	766.67	650.00
	SD	283.95	202.07	0.00
	Median	650.00	650.00	650.00
	Min	325.00	650.00	650.00
	Max	1000.00	1000.00	650.00
Day 5	N	6	2	2
	Mean	654.17	825.00	650.00
	SD	213.55	247.49	0.00
	Median	650.00	825.00	650.00
	Min	325.00	650.00	650.00
	Max	1000.00	1000.00	650.00
Day 6	N	2	0	2
	Mean	662.50	-	650.00
	SD	477.30	-	0.00
	Median	662.50	-	650.00
	Min	325.00	-	650.00
	Max	1000.00	-	650.00
Day 7	N	2	0	1
	Mean	662.50	-	400.00
	SD	477.30	-	.
	Median	662.50	-	400.00
	Min	325.00	-	400.00
	Max	1000.00	-	400.00
Day 8	N	2	1	0

Acetaminophen, Ibuprofen		The placebo group N=98	The low dose group N=99	The high dose group N=96
	Mean	662.50	1000.00	-
	SD	477.30	.	-
	Median	662.50	1000.00	-
	Min	325.00	1000.00	-
	Max	1000.00	1000.00	-
Day 9	N	3	0	0
	Mean	641.67	-	-
	SD	339.42	-	-
	Median	600.00	-	-
	Min	325.00	-	-
	Max	1000.00	-	-
Day 10	N	2	1	0
	Mean	662.50	1000.00	-
	SD	477.30	.	-
	Median	662.50	1000.00	-
	Min	325.00	1000.00	-
	Max	1000.00	1000.00	-
Day 11	N	2	0	1
	Mean	662.50	-	300.00
	SD	477.30	-	.
	Median	662.50	-	300.00
	Min	325.00	-	300.00
	Max	1000.00	-	300.00
Day 12	N	1	0	0
	Mean	500.00	-	-
	SD	.	-	-
	Median	500.00	-	-
	Min	500.00	-	-
	Max	500.00	-	-
Day 13	N	1	0	1
	Mean	1000.00	-	300.00
	SD	.	-	.
	Median	1000.00	-	300.00
	Min	1000.00	-	300.00
	Max	1000.00	-	300.00

Acetaminophen, Ibuprofen		The placebo group N=98	The low dose group N=99	The high dose group N=96
Day 14	N	0	1	0
	Mean	-	300.00	-
	SD	-	.	-
	Median	-	300.00	-
	Min	-	300.00	-
	Max	-	300.00	-
Day 15	N	0	0	0
	Mean	-	-	-
	SD	-	-	-
	Median	-	-	-
	Min	-	-	-
	Max	-	-	-
Day 16	N	1	0	0
	Mean	500.00	-	-
	SD	.	-	-
	Median	500.00	-	-
	Min	500.00	-	-
	Max	500.00	-	-
Day 17	N	0	0	0
	Mean	-	-	-
	SD	-	-	-
	Median	-	-	-
	Min	-	-	-
	Max	-	-	-
Day 18	N	0	0	0
	Mean	-	-	-
	SD	-	-	-
	Median	-	-	-
	Min	-	-	-
	Max	-	-	-
Day 19	N	0	0	0
	Mean	-	-	-
	SD	-	-	-
	Median	-	-	-
	Min	-	-	-

Acetaminophen, Ibuprofen		The placebo group N=98	The low dose group N=99	The high dose group N=96
	Max	-	-	-
Day 20	N	0	0	0
	Mean	-	-	-
	SD	-	-	-
	Median	-	-	-
	Min	-	-	-
	Max	-	-	-
Day 21	N	0	0	0
	Mean	-	-	-
	SD	-	-	-
	Median	-	-	-
	Min	-	-	-
	Max	-	-	-
Day 22	N	0	1	0
	Mean	-	1000.00	-
	SD	-	.	-
	Median	-	1000.00	-
	Min	-	1000.00	-
	Max	-	1000.00	-
Day 23	N	0	0	0
	Mean	-	-	-
	SD	-	-	-
	Median	-	-	-
	Min	-	-	-
	Max	-	-	-
Day 24	N	0	0	0
	Mean	-	-	-
	SD	-	-	-
	Median	-	-	-
	Min	-	-	-
	Max	-	-	-
Day 25	N	0	1	0
	Mean	-	1000.00	-
	SD	-	.	-
	Median	-	1000.00	-

Acetaminophen, Ibuprofen		The placebo group N=98	The low dose group N=99	The high dose group N=96
	Min	-	1000.00	-
	Max	-	1000.00	-
Day 26	N	0	1	0
	Mean	-	325.00	-
	SD	-	.	-
	Median	-	325.00	-
	Min	-	325.00	-
	Max	-	325.00	-
Day 27	N	0	0	0
	Mean	-	-	-
	SD	-	-	-
	Median	-	-	-
	Min	-	-	-
	Max	-	-	-
Day 28	N	0	0	0
	Mean	-	-	-
	SD	-	-	-
	Median	-	-	-
	Min	-	-	-
	Max	-	-	-

Table 44. Secondary endpoint 8) Acetaminophen, Ibuprofen dosages (mg) (PPS)

Acetaminophen, Ibuprofen		The placebo group N=77	The low dose group N=70	The high dose group N=80
Day 1	N	13	16	13
	Mean	573.08	646.88	638.46
	SD	139.37	163.78	41.60
	Median	650.00	650.00	650.00
	Min	200.00	350.00	500.00
	Max	650.00	1000.00	650.00
Day 2	N	3	12	6
	Mean	766.67	650.00	650.00
	SD	202.07	0.00	0.00
	Median	650.00	650.00	650.00
	Min	650.00	650.00	650.00
	Max	1000.00	650.00	650.00

Acetaminophen, Ibuprofen		The placebo group N=77	The low dose group N=70	The high dose group N=80
Day 3	N	2	6	4
	Mean	650.00	650.00	650.00
	SD	0.00	0.00	0.00
	Median	650.00	650.00	650.00
	Min	650.00	650.00	650.00
	Max	650.00	650.00	650.00
Day 4	N	0	2	2
	Mean	-	825.00	650.00
	SD	-	247.49	0.00
	Median	-	825.00	650.00
	Min	-	650.00	650.00
	Max	-	1000.00	650.00
Day 5	N	1	1	2
	Mean	650.00	1000.00	650.00
	SD	.	.	0.00
	Median	650.00	1000.00	650.00
	Min	650.00	1000.00	650.00
	Max	650.00	1000.00	650.00
Day 6	N	0	0	2
	Mean	-	-	650.00
	SD	-	-	0.00
	Median	-	-	650.00
	Min	-	-	650.00
	Max	-	-	650.00
Day 7	N	0	0	0
	Mean	-	-	-
	SD	-	-	-
	Median	-	-	-
	Min	-	-	-
	Max	-	-	-
Day 8	N	0	0	0
	Mean	-	-	-
	SD	-	-	-
	Median	-	-	-
	Min	-	-	-

Acetaminophen, Ibuprofen		The placebo group N=77	The low dose group N=70	The high dose group N=80
	Max	-	-	-
Day 9	N	0	0	0
	Mean	-	-	-
	SD	-	-	-
	Median	-	-	-
	Min	-	-	-
	Max	-	-	-
Day 10	N	0	0	0
	Mean	-	-	-
	SD	-	-	-
	Median	-	-	-
	Min	-	-	-
	Max	-	-	-
Day 11	N	0	0	1
	Mean	-	-	300.00
	SD	-	-	.
	Median	-	-	300.00
	Min	-	-	300.00
	Max	-	-	300.00
Day 12	N	0	0	0
	Mean	-	-	-
	SD	-	-	-
	Median	-	-	-
	Min	-	-	-
	Max	-	-	-
Day 13	N	0	0	1
	Mean	-	-	300.00
	SD	-	-	.
	Median	-	-	300.00
	Min	-	-	300.00
	Max	-	-	300.00
Day 14	N	0	1	0
	Mean	-	300.00	-
	SD	-	.	-
	Median	-	300.00	-

Acetaminophen, Ibuprofen		The placebo group N=77	The low dose group N=70	The high dose group N=80
	Min	-	300.00	-
	Max	-	300.00	-
Day 15	N	0	0	0
	Mean	-	-	-
	SD	-	-	-
	Median	-	-	-
	Min	-	-	-
	Max	-	-	-
Day 16	N	1	0	0
	Mean	500.00	-	-
	SD	.	-	-
	Median	500.00	-	-
	Min	500.00	-	-
	Max	500.00	-	-
Day 17	N	0	0	0
	Mean	-	-	-
	SD	-	-	-
	Median	-	-	-
	Min	-	-	-
	Max	-	-	-
Day 18	N	0	0	0
	Mean	-	-	-
	SD	-	-	-
	Median	-	-	-
	Min	-	-	-
	Max	-	-	-
Day 19	N	0	0	0
	Mean	-	-	-
	SD	-	-	-
	Median	-	-	-
	Min	-	-	-
	Max	-	-	-
Day 20	N	0	0	0
	Mean	-	-	-
	SD	-	-	-

Acetaminophen, Ibuprofen		The placebo group N=77	The low dose group N=70	The high dose group N=80
	Median	-	-	-
	Min	-	-	-
	Max	-	-	-
Day 21	N	0	0	0
	Mean	-	-	-
	SD	-	-	-
	Median	-	-	-
	Min	-	-	-
	Max	-	-	-
Day 22	N	0	0	0
	Mean	-	-	-
	SD	-	-	-
	Median	-	-	-
	Min	-	-	-
	Max	-	-	-
Day 23	N	0	0	0
	Mean	-	-	-
	SD	-	-	-
	Median	-	-	-
	Min	-	-	-
	Max	-	-	-
Day 24	N	0	0	0
	Mean	-	-	-
	SD	-	-	-
	Median	-	-	-
	Min	-	-	-
	Max	-	-	-
Day 25	N	0	0	0
	Mean	-	-	-
	SD	-	-	-
	Median	-	-	-
	Min	-	-	-
	Max	-	-	-
Day 26	N	0	0	0
	Mean	-	-	-

Acetaminophen, Ibuprofen		The placebo group N=77	The low dose group N=70	The high dose group N=80
	SD	-	-	-
	Median	-	-	-
	Min	-	-	-
	Max	-	-	-
Day 27	N	0	0	0
	Mean	-	-	-
	SD	-	-	-
	Median	-	-	-
	Min	-	-	-
	Max	-	-	-
Day 28	N	0	0	0
	Mean	-	-	-
	SD	-	-	-
	Median	-	-	-
	Min	-	-	-
	Max	-	-	-

Table 45. Secondary endpoint 8) the number of administrations of antidiarrheals (ITT)

Antidiarrheals		The placebo group N=98	The low dose group N=99	The high dose group N=96
Day 2	N	0	0	1
	Mean	-	-	1.00
	SD	-	-	0.00
	Median	-	-	1.00
	Min	-	-	1.00
	Max	-	-	1.00
Day 3	N	0	1	0
	Mean	-	1.00	-
	SD	-	0.00	-
	Median	-	1.00	-
	Min	-	1.00	-
	Max	-	1.00	-
Day 4	N	0	3	0
	Mean	-	1.00	-

	SD	-	0.00	-
	Median	-	1.00	-
	Min	-	1.00	-
	Max	-	1.00	-
Day 5	N	0	3	1
	Mean	-	1.00	1.00
	SD	-	0.00	0.00
	Median	-	1.00	1.00
	Min	-	1.00	1.00
	Max	-	1.00	1.00

Table 46. Secondary endpoint 8) the number of administrations of antidiarrheals (PPS)

Antidiarrheals		The placebo group N=77	The low dose group N=70	The high dose group N=80
Day 1–3	N	0	0	0
	Mean	-	-	-
	SD	-	-	-
	Median	-	-	-
	Min	-	-	-
	Max	-	-	-
Day 4	N	0	2	0
	Mean	-	1.00	-
	SD	-	0.00	-
	Median	-	1.00	-
	Min	-	1.00	-
	Max	-	1.00	-
Day 5	N	0	3	0
	Mean	-	1.00	-
	SD	-	0.00	-
	Median	-	1.00	-
	Min	-	1.00	-
	Max	-	1.00	-
Day 6–28	N	0	3	0
	Mean	-	1.00	-
	SD	-	0.00	-

	Median	-	1.00	-
	Min	-	1.00	-
	Max	-	1.00	-

9) Proportion of participants with severe COVID-19 progression from Day 1 to Day 28

Over the study period from Day 1 through Day 28, there were no participants with severe COVID-19 progression.

Table 47. Secondary endpoint 9) participants progressed to severe COVID-19 (ITT)

Participants who progressed to severe COVID-19 or not		The placebo group N=98 n (%)	The low dose group N=99 n (%)	The high dose group N=96 n (%)
Day 1-Day 28	Total<br>Progressed to severity<br>Not progressed to severity	98(100.0)<br>0(0.0)<br>98(100.0)	99(100.0)<br>0(0.0)<br>99(100.0)	96(100.0)<br>0(0.0)<br>96(100.0)

10) Pharmacokinetic characteristics of niclosamide and correlation between pharmacokinetic parameters and viral load

Table 48 presents the descriptive statistics of the pharmacokinetic variables. In the high dose group, participant R03031 was dropped out before dosing, and for participant R03060, pharmacokinetic blood sampling was not conducted due to a consent error related to pharmacokinetic blood collection.

The geometric mean of Cmax for the low dose group was 285.25 ng/mL (minimum 139.82, maximum 936.53), while for the high dose group, it was 389.90 ng/mL (minimum 129.39, maximum 1061.79).

Table 48. Secondary endpoint 10) descriptive statistics of pharmacokinetic variables for niclosamide (ITT)

Treatment	Parameters	N	Arithmetic Mean	SD	Geometric Mean	Median	Min	Max
The low dose (N=20)	Cmax (ng/mL)	20	317.78	186.42	285.25	246.39	139.82	936.53
	AUCt (ng·h/mL)	20	11046.21	3451.07	10562.09	9754.47	6479.00	17836.36
The high dose (N=18)	Cmax (ng/mL)	18	460.44	275.45	389.90	391.83	129.39	1061.79
	AUCt (ng·h/mL)	18	13969.54	5880.64	12876.29	12912.31	6809.88	24605.15

Table 49. Mean and confidence interval for niclosamide AUC and Cmax (ITT)

Geometric Mean			LSMean Ratio (T/R)		ANOVA-CV
			Point Estimate	90% CI	
AUCt	The low dose	10562.09	1.2191	[1.0000,1.4862]	36.12
	The high dose	12876.29			

	Geometric Mean		LSMean Ratio (T/R) Point Estimate	90% CI	ANOVA-CV
Cmax	The low dose	285.25	1.3669	[1.0277,1.8180]	51.99
	The high dose	389.90			

- Correlation between niclosamide and viral load

To determine the correlation between niclosamide AUCt and viral load (RdRp gene) measured by qPCR, results using the CORR procedure (correlation analysis) of the SAS® program are presented in Table 50, and scatter plots are shown in Figure 1 (low dose) and Figure 2 (high dose). For the correlation analysis, niclosamide AUCt was calculated for AUCday0, AUCDay2, AUCDay4, and AUCDay6, and correlation analysis was conducted with the viral load figures corresponding to each date.

There was a significant negative correlation between niclosamide AUCt and viral load in both the low dose and the high dose groups. Each correlation coefficient (p-value) was -0.3296 (p=0.0101) for the low dose group and -0.4818 (p=0.0009) for the high dose group, showing that the negative correlation was more significant in higher doses. In other words, it was found that as the administered dose increased, the viral load decreased more significantly.

Table 50. Secondary endpoint 10) correlation between niclosamide and viral load (ITT)

Niclosamide AUCt vs Viral load			Niclosamide AUCt	Viral load
The low dose	Pearson correlation coefficient	Niclosamide AUCt	1.0000	-0.3296
		viral load	-0.3296	1.0000
	Significance probability	Niclosamide AUCt	-	0.0101
		viral load	0.0101	-
The high dose	Pearson correlation coefficient	Niclosamide AUCt	1.0000	-0.4818
		viral load	-0.4818	1.0000
	Significance probability	Niclosamide AUCt		0.0009
		viral load	0.0009	

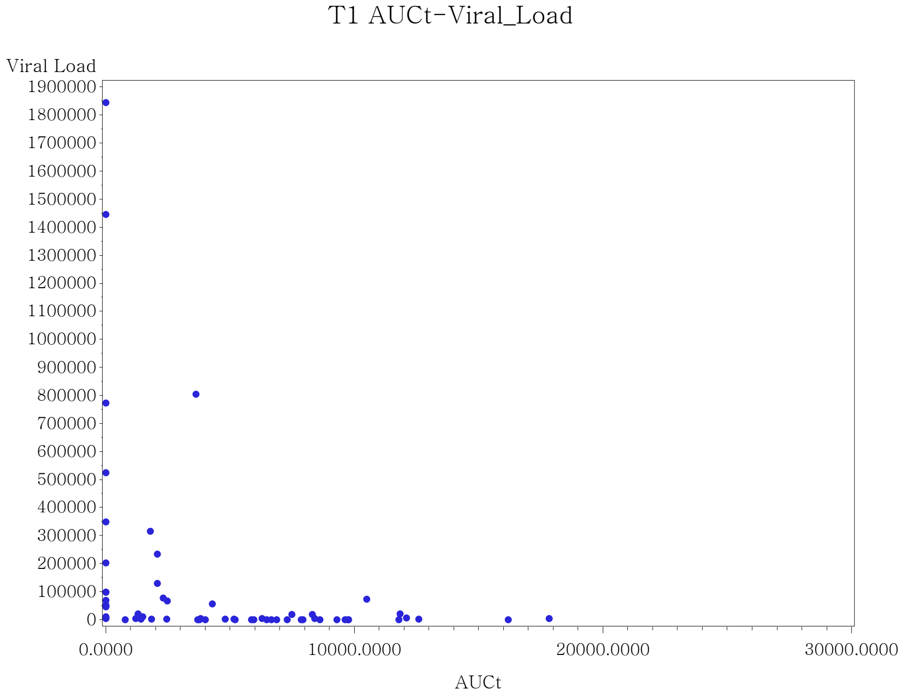

<Figure 1. Scatter plot of niclosamide AUCt and viral load (qPCR-RdRp gene) for the low dose group>

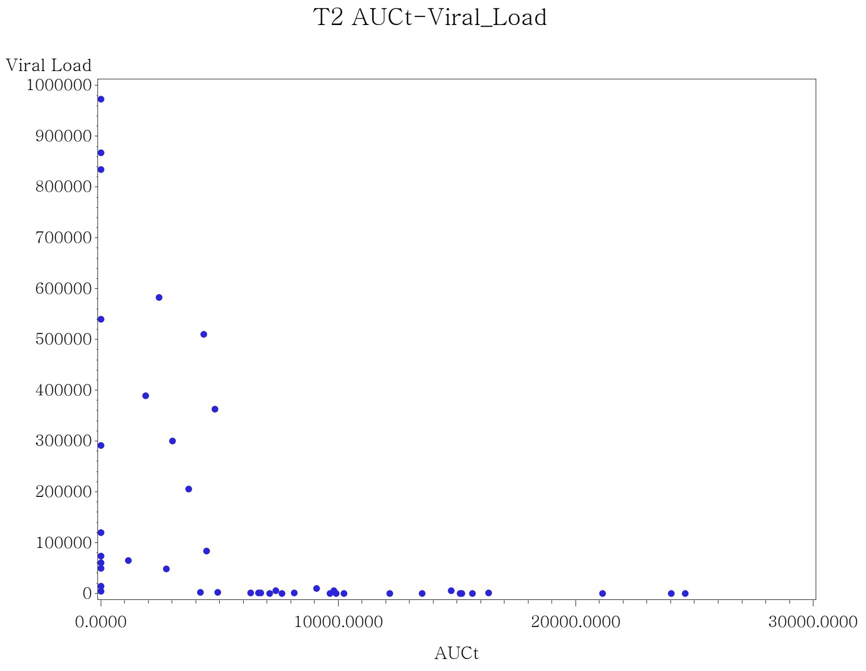

<Figure 2. Scatter plot of niclosamide AUCt and viral load (qPCR-RdRp gene) for the high dose group>

Table 51. 28 Days full data related to recovery (ITT, PPS, mITT-1 and mITT-2)

Time (days) for sustained improvement of all symptoms (ITT, n=293)	The placebo group (N=98)	The low dose group (N=96)	The high dose group (N=99)
Median to Improvement [95% CI]	13.50 [11.00, 17.00]	10.50 [9.00, 18.00]	14.50 [12.50, 18.50]
Difference vs. placebo	-	3.00	-1.00
p-value	-	0.9305	0.2949

Time (days) for sustained improvement of all symptoms (PPS, n=227)	The placebo group (N=77)	The low dose group (N=70)	The high dose group (N=80)
Median to Improvement [95% CI]	15.00 [11.50, 19.00]	9.25 [7.50, 12.50]	13.25 [11.50, 18.50]
Difference vs. placebo	-	5.75	1.75
p-value	-	0.0275	0.8310

Time (days) for sustained improvement of all symptoms (mITT1, n=264)	The placebo group (N=88)	The low dose group (N=89)	The high dose group (N=87)
Median to Improvement [95% CI]	14.00 [11.00, 18.50]	10.00 [7.50, 14.50]	14.50 [12.00, 18.50]
Difference vs. placebo	-	4.00	-0.50
p-value	-	0.0898	0.9411

Time (days) for sustained improvement of all symptoms (mITT2, n=253)	The placebo group (n=85)	The low dose group (n=86)	The high dose group (n=82)
Median to Improvement [95% CI]	14.00 [11.00, 19.00]	10.50 [8.00, 15.00]	15.00 [12.00, 18.50]
Difference vs. placebo	-	3.50	-1.00
p-value	-	0.1009	0.8412

Supplementary References

1. Treanor JJ, Hayden FG, Vrooman PS, Barbarash R, Bettis R, Riff D, Singh S, Kinnersley N, Ward P, Mills RG. Efficacy and safety of the oral neuraminidase inhibitor oseltamivir in treating acute influenza: a randomized controlled trial. US Oral Neuraminidase Study Group. JAMA. 2000 Feb 23;283(8):1016-24. doi: 10.1001/jama.283.8.1016. PMID: 10697061.

2. Hammond, J., et al. Oral Nirmatrelvir for High-Risk, Nonhospitalized Adults with Covid-19. *N. Engl. J. Med*. **386**, 1397–1408 (2022).

3. Choi, G., et al. The Next Generation COVID-19 Antiviral; Niclosamide-Based Inorganic Nanohybrid System Kills SARS-CoV-2. *Small* e2305148 (2023).

4. Brown, E.G., Wood, L., Wood, S. The medical dictionary for regulatory activities (MedDRA). Drug Saf **20**, 109–117 (1999).

5. Lórenz-Fonfría, V.A., Muders, V., Schlesinger, R., Heberle, J. Changes in the hydrogen-bonding strength of internal water molecules and cysteine residues in the conductive state of channelrhodopsin-1. *J. Chem. Phys.* **141**, 22D507 (2014).

6. Yu, S., et al. Niclosamide–clay intercalate coated with nonionic polymer for enhanced bioavailability toward covid-19 treatment. *Polymers* **13**, 1044 (2021).

7.. Choi, G., et al. Hydrotalcite–niclosamide nanohybrid as oral formulation towards SARS-CoV-2 viral infections. *Pharmaceuticals* **14**, 486 (2021).

8. Al-Hadiya, B.M. Niclosamide: comprehensive profile. *Profiles Drug Subst. Excip. Relat. Method* **32**, 67–96

(2005).

9. Zapata, F., et al. Introducing ATR-FTIR spectroscopy through analysis of acetaminophen drugs: Practical lessons for interdisciplinary and progressive learning for undergraduate students. *J. Chem. Educ.* **98**, 2675–2686 (2021).

10. Liu, H., Wang, Y., Bowman, J.M. Quantum Local Monomer IR Spectrum of Liquid D2O at 300 K from 0 to 4000 cm–1 Is in Near-Quantitative Agreement with Experiment. *J. Phys. Chem. B* **120**, 2824–2828 (2016).

11. Belhadj, H., Hakki, A., Robertson, P.K., Bahnemann, D.W. In situ ATR-FTIR study of H_2O and D_2O adsorption on TiO_2 under UV irradiation. *Phys. Chem. Chem. Phys.* **17**, 22940–22946 (2015).

12. Gardner EA, Nevarez A, Garbalena M, Herndon WC. Infrared spectra and conformational analysis of anti and gauche deuterated ethanol isotopomers. *J. Mol. Struct.* **784**, 249–253 (2006).

13. Keresztury G, Billes F, Kubinyi M, Sundius T. A density functional, infrared linear dichroism, and normal coordinate study of phenol and its deuterated derivatives: revised interpretation of the vibrational spectra. *J. Phys. Chem. A* **102**, 1371–1380 (1998).

14. Zheng, J., et al. Ultrafast dynamics of solute-solvent complexation observed at thermal equilibrium in real time. *Science* **309**, 1338–1343 (2005).

15. Jesionek, P., et al. The impact of H/D exchange on the thermal and structural properties as well as high-pressure relaxation dynamics of melatonin. *Sci. Rep.* **12**, 14324 (2022).

16. Patel, M.K., et al. A label-free photoluminescence genosensor using nanostructured magnesium oxide for cholera detection. *Sci. Rep.* **5**, 17384 (2015).

17. Kooi, B.J., Palasantzas, G., De Hosson, J.T.M. Gas-phase synthesis of magnesium nanoparticles: A high-resolution transmission electron microscopy study. *Appl. Phys. Lett.* **89**, 161914 (2006).

18. Montero, J.M., Isaacs, M.A., Lee, A.F., Lynam, J.M., Wilson, K. The surface chemistry of nanocrystalline MgO catalysts for FAME production: An in situ XPS study of H_2O, CH_3OH and CH_3OAc adsorption. *Surf. Sci.* **646**, 170–178 (2016).
